# The effects of SCFAs on glycemic control in humans: a systematic review and meta-analysis

**DOI:** 10.1093/ajcn/nqac085

**Published:** 2022-04-07

**Authors:** Anna Cherta-Murillo, Jennifer E Pugh, Sumayya Alaraj-Alshehhi, Dana Hajjar, Edward S Chambers, Gary S Frost

**Affiliations:** Section for Nutrition Research, Department of Metabolism, Digestion and Reproduction, Faculty of Medicine, Imperial College London, Hammersmith Hospital, London, United Kingdom; Section for Nutrition Research, Department of Metabolism, Digestion and Reproduction, Faculty of Medicine, Imperial College London, Hammersmith Hospital, London, United Kingdom; Section for Nutrition Research, Department of Metabolism, Digestion and Reproduction, Faculty of Medicine, Imperial College London, Hammersmith Hospital, London, United Kingdom; Section for Nutrition Research, Department of Metabolism, Digestion and Reproduction, Faculty of Medicine, Imperial College London, Hammersmith Hospital, London, United Kingdom; Section for Nutrition Research, Department of Metabolism, Digestion and Reproduction, Faculty of Medicine, Imperial College London, Hammersmith Hospital, London, United Kingdom; Section for Nutrition Research, Department of Metabolism, Digestion and Reproduction, Faculty of Medicine, Imperial College London, Hammersmith Hospital, London, United Kingdom

**Keywords:** short-chain fatty acids, acetate, propionate, butyrate, glycemic control, systematic review, meta-analysis, insulin

## Abstract

**Background:**

Noncommunicable disease development is related to impairments in glycemic and insulinemic responses, which can be modulated by fiber intake. Fiber's beneficial effects upon metabolic health can be partially attributed to the production of SCFAs via microbial fermentation of fiber in the gastrointestinal tract.

**Objectives:**

We aimed to determine the effects of SCFAs, acetate, propionate, and butyrate on glycemic control in humans.

**Methods:**

The CENTRAL, Embase, PubMed, Scopus, and Web of Science databases were searched from inception to 7 December 2021. Papers were included if they reported a randomized controlled trial measuring glucose and/or insulin compared to a placebo in adults. Studies were categorized by the type of SCFA and intervention duration. Random-effects meta-analyses were performed for glucose and insulin for those subject categories with ≥3 studies, or a narrative review was performed.

**Results:**

We identified 43 eligible papers, with 46 studies within those records (*n* = 913), and 44 studies were included in the meta-analysis. Vinegar intake decreased the acute glucose response [standard mean difference (SMD), −0.53; 95% CI, −0.92 to −0.14; *n* = 67] in individuals with impaired glucose tolerance or type 2 diabetes and in healthy volunteers (SMD, −0.27; 95% CI, −0.54 to 0.00; *n* = 186). The meta-analyses for acute acetate, as well as acute and chronic propionate studies, showed no significant effect.

**Conclusions:**

Vinegar decreased the glucose response acutely in healthy and metabolically unhealthy individuals. Acetate, propionate, butyrate, and mixed SCFAs had no effect on blood glucose and insulin in humans. Significant heterogeneity, risks of bias, and publication biases were identified in several study categories, including the acute vinegar glucose response. As evidence was very uncertain, caution is urged when interpreting these results. Further high-quality research is required to determine the effects of SCFAs on glycemic control.

## Introduction

Noncommunicable diseases, such as type 2 diabetes (T2D) and cardiovascular disease, accounted for 44% of global deaths in 2019 (1). T2D diagnoses have quadrupled globally, from 108 million to 422 million, in the last 40 years (2). Elevations in blood glucose and insulin play a significant role in noncommunicable disease development, specifically of T2D (3–6). Improving glycemic control can reduce the risk of complications associated with T2D (7).

Diet is a primary risk factor for the development of noncommunicable diseases. Western diets are often nutrient deficient, energy dense, and low in fiber (8), and populations following Western dietary patterns have high incidences of chronic disease (9, 10). Fiber intake plays a determining role in the noncommunicable disease risk and is a strong indicator of the all-cause chronic disease mortality risk (11). Previous human nutrition studies have shown that dietary fibers have a beneficial effect on glycemia (12).

Dietary fiber passes through the upper gastrointestinal tract undigested and can act as a substrate for bacterial fermentation throughout the gut (13). After undergoing fermentation in the gastrointestinal tract, 10 g of fiber yields approximately 100 mmol/L of SCFAs. Acetate, butyrate, and propionate are produced in the largest quantities at a molar ratio of 3:1:1, respectively (14). SCFAs activate G-protein-coupled receptors, known as free-fatty-acid receptors (FFAR) 2 and 3, which are expressed in the gut and in metabolically active tissues, such as in the liver, adipocytes, myocytes, and pancreas (15). In vitro and animal studies have shown SCFAs influence the glucose metabolism in glucose-disposal tissues, such as hepatocytes (16), adipocytes (17), and myocytes (18). These SCFAs have been shown to directly stimulate the release of anorectic hormones, such as glucagon-like peptide 1 (GLP-1) and peptide YY, in colonic enteroendocrine cells (19–21). However, increasing fiber intake at the population level has proven challenging. Hence, providing a similar metabolic benefit via alternative methods is the aim of much current research.

Overall, evidence suggests that SCFAs may influence human glucose homeostasis. Compiling the studies exploring the impacts of SCFAs on glycemic control may help to elucidate the therapeutic potential of SCFAs within the systemic circulation and gut when administered at concentrations at or above that produced when the recommended fiber intake (30 g/d) is consumed. Here, we aim to investigate the effects of SCFA administration on glycemic control.

## Methods

We performed a systematic review of peer-reviewed literature published since inception. The systematic review was conducted in adherence with the Preferred Reporting Items for Systematic Reviews and Meta-Analyses (PRISMA) guidelines (22). The formal screening of papers began on 15 November 2020, and the registration of the protocol for this review to PROSPERO was submitted on 14 January 2021 with the reference CRD42021231115.

### Eligibility criteria

The PICOS (patients, intervention, comparator, outcomes, and study design) criteria were used to establish study eligibility (**Table 1**).

**TABLE 1:** PICOS criteria for study eligibility^1^

	Inclusion	Exclusion
Participants	Humans who are healthy, overweight, or obese and have metabolic syndrome or type 2 diabetes	Other type of diseased humans and animals. Humans undergoing clamps, such as a hypoglycemic or hyperinsulinemic euglycemic clamp, which do not represent a real physiological setting
Intervention	Acetate, propionate, butyrate alone or mixed, and vinegar administration. Both acute (for 24-hour) and chronic (over 24-hour) administrations	SCFA conjugated with drugs or hormones
Comparator	Placebo	Against diseased humans, between different doses of SCFA
Outcome	Quantifiable measures of glycemic control as the main or secondary outcome, such as fasting glucose or insulin, postprandial glucose or insulin (i.e., AUC), HbA1c, insulin sensitivity indexes (i.e., HOMA-IR, clamps)	Studies which do not include a quantifiable measure of the outcomes of interest
Study design	Study designs that generate empirical data from interventional studies that are randomized controlled trials. Only results analyzed statistically will be included	Reviews, conference abstracts, dissertation abstracts, lectures, information pieces, study registers and *corrigendums* were not included. Studies were limited to those in the English language published from 1980 onwards

1 Abbreviations: HbA1c, glycated hemoglobin A1c; PICOS, populations, intervention, comparison, outcomes, and study design.

### Search strategy

The online databases PubMed (Medline), Cochrane CENTRAL, EMBASE, Web of Science, and Scopus were used to identify records published from inception to 7 December 2021. The search algorithm used for each database is described in **Supplemental Table 1**. In addition, a manual search of reference lists of reviews on the topic was performed, to identify additional relevant articles. When necessary, the authors were contacted to obtain data of interest. Studies were excluded if authors did not respond.

### Study selection

The study selection was performed using the online software Covidence systematic review software (Veritas Health Innovation; www.covidence.org). All articles identified by the search strategy were screened by title and abstract by 2 reviewers independently (SA-A and JEP). After screening, full texts deemed to be potentially relevant were assessed for eligibility against the defined inclusion and exclusion criteria independently (SA-A and JEP; Table 1). During the study screening and assessment, any discrepancies in the eligibility of papers were resolved by consulting a third party (AC-M). Excluded studies and reasons for exclusion can be found in **Supplemental Table 2**.

### Data extraction and quantification

Data were extracted by 4 reviewers independently (SA-A, AC-M, DH, and JEP). Articles deemed eligible for inclusion were assigned to subject categories according to the nature of the study intervention (acute or chronic) and type of SCFA [acetate, butyrate, propionate, mixed or vinegar (acetic acid)]. Study characteristics were extracted for each category, including the authors’ names, publication year, study design, length of intervention [acute (<24 hours) or chronic], sample size, participants’ demographic characteristics (gender, age, BMI, and any health conditions), SCFA concentration, route of administration (oral, intravenous, or gastrointestinal), measurement period, energy and macronutrient matching, and outcomes analyzed. Some identified records contained multiple studies, which were extracted individually. Whilst all comparisons within the same record were captured in the tables summarizing the studies, not all comparisons were meta-analyzed. Selection of the comparisons against the control was based on the highest dose or on the format used in real life (e.g., liquid vinegar over pill). **Table 2** summarizes the eligible study characteristics.

**TABLE 2:** Summary of the acetate studies’ design, participant, and intervention characteristics and outcomes analyzed^1^

		Participant characteristics				Intervention characteristics				Glycemic outcome analyzed
Reference	Study design	Health status	Sample size (M/F)	Age, y	BMI, kg/m^2^	Amount given (rate)	Route of administration	Duration	Control	
Acute										
Scheppach et al., 1988(study 1) (28)	RCT, XO	HV	5	33	22.4	195 mmol (15 mmol/15 min) +50 g CHO	Oral (drink)	360 minutes	Chloride	PBG PI
Laurent et al., 1995(36)	RCT, XO	HV	6 (3/3)	22	21.2	12 mmol/h	Intragastric (infusion)	300 minutes	Saline	PBG PI
Freeland and Wolever, 2010 (37)	RCT, XO	Hyperinsulinemic	6 (0/6)	44	31	20 mmol/L (12.5 ml/min)	Intravenous (infusion)	60 minutes	Saline IV	PBG PI
						60 mmol/L (37.5 ml/min)	Rectal (infusion)	60 minutes	Saline R	PBG PI
Van der Beek et al.,2016 (35)	RCT, XO	HV	6 (6/0)	35	31	100–180 mmol/L	Colonic (infusion)	300 min/d x 3 days	Saline	PBG PI

1 Abbreviations: CHO, carbohydrate; F, female; HV, healthy volunteers; IV, intravenous; M, male; PBG, postprandial blood glucose; PI, postprandial insulin; R, rectal; RCT, randomized controlled trial; XO, crossover.

### Data synthesis and statistical analysis

Descriptive data were reported as the mean ± SD unless otherwise stated. Glycemic control measurements included the blood glucose and insulin (raw or change from baseline) AUC or incremental AUC (iAUC), fasting blood glucose (FBG) or insulin, glycated hemoglobin (Hb1Ac; as a percentage), or insulin sensitivity indexes (e.g., HOMA-IR). For the subject categories that included <3 studies, a narrative review was conducted in accordance with the Cochrane Handbook for Systematic Reviews of Interventions (23). For categories that had ≥3 studies, a meta-analysis was performed. For the meta-analysis, raw data or changes from the baseline iAUC were extracted, and the variance was transformed to SD. When data were available as individual time points, means and variances were extracted using the online tool WebPlotDigitizer 4.4 (Ankit Rohatgi) (https://apps.automeris.io/). Then, the iAUC of the mean and variance was calculated by the trapezoidal rule (24).

### Meta-analysis

A meta-analysis to estimate the pooled effects of the SCFAs on the different glycemic outcomes was performed for each subject category that included ≥3 studies reporting the same glycemic outcome. These were: acute acetate, acute vinegar, and acute and chronic propionate administration. For acute studies, meta-analyses of postprandial blood glucose (PBG) and insulin iAUC were performed. For chronic studies, meta-analyses of PBG and insulin iAUC, fasting glucose and insulin, and glycated hemoglobin (HbA1c) were performed. For these outcomes for each subject category, the weighted effect estimates, reported as the standard mean differences (SMDs) and corresponding 95% CIs, were calculated as using a Sidik-Jonkman random-effects model to allow a wide 95% CI in order to reflect uncertainty in the estimation of between-study heterogeneity. For crossover studies, the SMD was calculated by assuming a parallel design for a more conservative analysis. Heterogeneity was assessed using the I^2^ statistic and visual inspection of the Galbraith plot (**Supplemental Figures 1.2, 2.2, 3.2, 4.2, 5.2, 6.2, 7.2, 8.2, 9.2, 10.2, 11.2, 12.2, 13.2**, and **14.2**). I^2^ values ranged from 0% to 100%, with values of 25% to 49%, 50% to 74%, and ≥75% classified as low, moderate, and high, respectively (25). CIs were determined using the “heterogi” command in Stata (StataCorp). Publication bias was assessed via funnel plots for each meta-analysis (Supplemental Figures 1–14). To assess influential studies, a sensitivity (leave-1-out) analysis was performed for all categories (**Supplemental Figures 1.1, 2.1, 3.1, 4.1, 5.1, 6.1, 7.1, 8.1, 9.1, 10.1, 11.1, 12.1, 13.1**, and **14.1**). Statistical significance was determined at a *P* value ≤0.05. All reported *P* values are 2-sided. All statistical analyses were performed with Stata 17.0.

### Risk-of-bias assessment

Studies were assessed for the risk of bias by 3 independent reviewers (SA-A, JEP, DH) following the revised Cochrane risk-of-bias tool for randomized trials (26). The 6 methodological features assessed were randomization, assignment of the intervention, adherence to the intervention, missing outcome data, measuring the outcome, and selection of the reported result. Studies were classified as having a high risk of bias if they contained methodological flaws that may have influenced the results, having a low risk if the flaw was not deemed to have affected the results, and having some concerns if not enough information was provided to pass a judgement. Disagreements in the classification were resolved by consulting a third party (AC-M).

### Certainty of evidence: Grading of Recommendations, Assessment, Development, and Evaluation

The table summarizing the findings was constructed using GRADEpro software (McMaster University and Evidence Prime Inc.) (http://gradepro.org, accessed 10 December 2021). Certainty of evidence was assessed using Grading of Recommendations, Assessment, Development, and Evaluation (GRADE) recommendations. The certainty of evidence was graded as high, moderate, low, and very low (27).

### Assessment of confounders of glycemic control

Studies that controlled for factors influencing the glycemic response may produce more accurate results. Acute confounders of glycemic control are physical activity, the length of the fasting period, and fiber or alcohol intake. Chronic confounders include changes in the body weight and body fat percentage over the course of the study. Included studies were assessed for how they controlled for elements known to confound glycemic control (e.g., body weight or body fat change, physical activity, or alcohol intake prior to the intervention or an overnight fast), and the results are summarized in **Supplemental Table 3**.

## Results

### Description of studies

**FIGURE 1:**
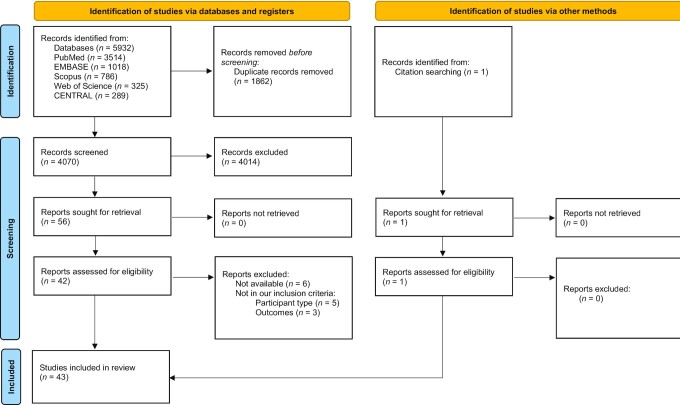
PRISMA flow diagram of references identified and evaluated. From Page et al. (22) For more information, visit http://www.prisma-statement.org/. Abbreviation: PRISMA, Preferred Reporting Items for Systematic Reviews and Meta-Analyses.

The systematic literature search produced a total of 5932 references following the database and manual search (**Figure 1**). Specifically, 3514 publications were identified from PubMed, 289 from Cochrane CENTRAL, 1018 from EMBASE, 325 from Web of Science, 786 from Scopus, and 1 from the manual search. Duplicates (*n* = 1862) were removed. After screening, 4014 records were excluded. A total of 56 full-text articles were assessed for eligibility against the PICOS criteria, 14 of which were excluded. This yielded 43 records to be included in this review. Some records had more than 2 intervention arms per test intervention or more than 2 studies within the same record. This was the case for acetate studies, such as that of Scheppach et al. (28) (2 studies within the same record), and vinegar studies, such as those of Brighenti et al. (29) (2 interventions compared with the same control), Johnston and Buller (30) (2 arms with different food matrices), Johnston et al. (31) (3 studies within the same record), Liatis et al. (32) (2 arms with different glycemic indexes), Darzi et al. (33) (2 studies within the same record), and Feise and Johnston (34) (3 interventions compared with the same control). Nevertheless, not all comparisons were meta-analyzed. The comparisons chosen for meta-analysis are described in the footnote of each forest plot. Therefore, from the 43 identified records, there were 52 studies within those records, 44 of which were included in the meta-analysis. Five investigated acetate (all acute interventions), 2 investigated butyrate (both reporting the same chronic study), 14 investigated propionate (8 acute and 6 chronic interventions), 31 studies investigated vinegar (25 acute and 6 chronic interventions), and 5 investigated mixed SCFAs.

**Tables2–6** describe the study design and participant characteristics of all eligible studies. In the interest of clarity, SCFA interventions will be referred to in the text in the simple forms of acetate, butyrate, or propionate. However, some studies have used different compounds of the SCFAs, such as sodium propionate.

### Risk of bias in included studies

**FIGURE 2:**
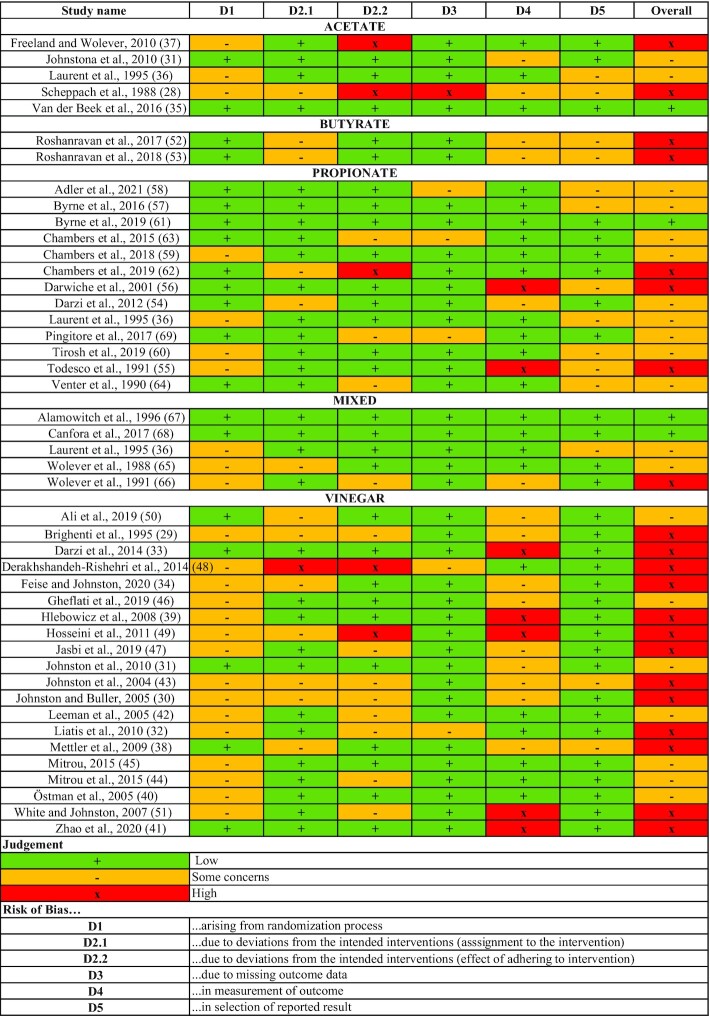
Risk of bias summary for all studies by length-intervention category (acute or chronic) and intervention (acetate, propionate, butyrate, vinegar, or mixed SCFAs).

The risks of bias for the included studies are described in **Figure 2**. Out of the studies, 47% were determined to have a high risk of bias, 44% to have a moderate risk of bias, and 9% to have a low risk of bias. The domains of greatest concern were risks of bias arising from deviations from intended interventions (D2.2), in measurements of the outcome (D4), and in the selection of reported results (D5).

### Effects of interventions

#### Acetate

The characteristics of the eligible acetate studies are summarized in Table 2.

#### Acute interventions

**FIGURE 3:**
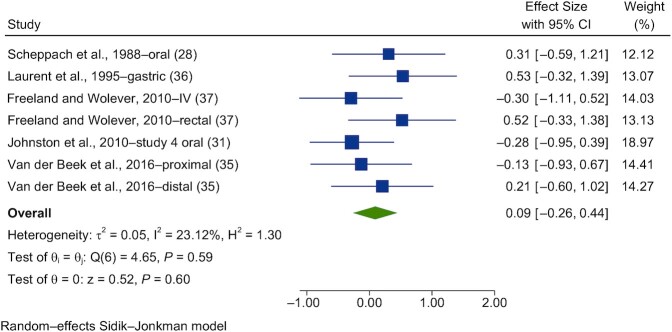
Forest plots for randomized controlled trials of acute acetate on postprandial blood glucose. Acute interventions with acetate had a main effect of 0.09 (95% CI, −0.26 to 0.44; *P* = 0.60) on the postintervention postprandial blood glucose iAUC (*n* = 44). Johnston et al. (31) study 4-oral, in T2D. A random-effects model was used to calculate SMDs (squares), 95% CIs (horizontal lines), and summary effects (SMD; diamond). The study weight (expressed as a percentage) indicates the relative contribution of an individual study to the overall pooled effect size. Between-study heterogeneity was calculated using the I^2^ statistic. A *P* value ≤ 0.05 was considered statistically significant. When interpreting SMDs (or effect sizes) values <0.40 were categorized as having a small effect size, values 0.40 to 0.70 as having a moderate effect size, and values >0.70 as having a large effect size. Abbreviations: iAUC, incremental AUC; IV, intravenous; SMD, standard mean difference; T2D, type 2 diabetes.

**FIGURE 4:**
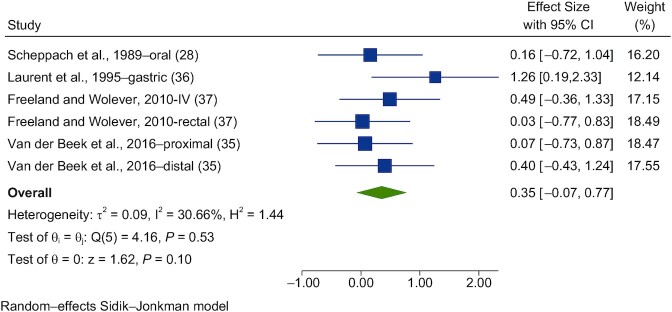
Forest plots for randomized controlled trials of acute acetate on postprandial blood insulin. Acute interventions with acetate had a main effect of 0.35 (95% CI, −0.07 to 0.77; *P* = 0.10) on the postintervention postprandial blood insulin iAUC (*n* = 35)_._ A random-effects model was used to calculate standardized mean differences (squares), 95% CIs (horizontal lines), and summary effects (SMD; diamond). The study weight (expressed as a percentage) indicates the relative contribution of an individual study to the overall pooled effect size. Between-study heterogeneity was calculated using the I^2^ statistic. A *P* value ≤ 0.05 was considered statistically significant. When interpreting SMDs (or effect sizes) values <0.40 were categorized as having a small effect size, values 0.40 to 0.70 as having a moderate effect size, and values >0.70 as having a large effect size. Abbreviations: iAUC, incremental AUC; SMD, standard mean difference.

Seven studies were meta-analyzed for blood glucose (28, 35–37) and 6 for insulin (28, 35–37). Forest plots of the pooled effects of acetate interventions on PBG and insulin are shown in **Figures 3** and **4**.

A random-effects model showed that acute acetate interventions had no effect on PBG (SMD = 0.09; 95% CI, −0.26 to 0.44; *n* = 44) and had nonsignificant interstudy heterogeneity (I^2^ = 23.1%; *P* = 0.59; 95% CI, 0–71; Figure 3). For insulin, acute acetate interventions had no significant effect on the postprandial blood insulin iAUC (SMD = 0.35; 95% CI, −0.07 to 0.77; *n* = 35) and had moderate interstudy heterogeneity (I^2^ = 30.7%; *P* = 0.53; 95% CI, 0–75; Figure 4)_._

Homogeneity via a Galbraith plot, publication bias via a funnel plot, and a sensitivity analysis via a leave-1-out plot were assessed and are reported in **Supplemental Figures 1** and **2.**

### Vinegar

The characteristics of the eligible vinegar studies are summarized in Table 3.

**TABLE 3:** Summary of the oral intake of vinegar studies design, participant and intervention characteristics and outcome analyzed^1^

Reference	Study design	Health status	Sample size (Male/Female)	Age, years	BMI, kg/m^2^	Amount	Type of vinegar	Acetic acid, %	Duration	Coingested with CHO, g	Control	Outcomes analyzed
Acute												
Brighenti et al., 1995 (29)	RCT, XO	HV	5 (4/1)	37	98	20 ml	White (acetic acid from vinegar)	5	95 min	50	White Bread	PBG
Johnston et al., 2004 (43)	RCT, XO	HV	8	NI	NI	20 g	Apple cider	5	60 minutes	87	Placebo drink (not specified)	PBG PI
Johnston et al., 2004-study a (43)	RCT, XO	T2D	11	NI	NI	20 g	Apple cider	5	60 minutes	87	Placebo drink (not specified)	PBG PI
Johnston et al., 2004-study b (43)	RCT, XO	IR	10	NI	NI	20 g	Apple cider	5	60 minutes	87	Placebo drink (not specified)	PBG PI
Johnston and Buller, 2005-study a (30)	RCT, XO	HV	11 (1/10)	27.9	22.7	20 g	Apple cider	5	60 minutes	87	Sweetened water	PBG PI
Johnston and Buller, 2005-study b (30)	RCT, XO	HV	11 (1/10)	27.9	22.7	20 g	Apple cider	5	60 minutes	52	Sweetened water	PBG PI
Leeman et al., 2005 (42)	RCT, XO	HV	13 (3/10)	19–32	22.5	28 g	White	6	120 minutes	50	Boiled potatoes	PBG PI
Östman et al., 2005 (40)	RCT, XO	HV	12 (10/2)	22.9	21.5	28 g	White	6	120 minutes	50	White bread	PBG PI
Hlebowicz et al., 2008 (39)	RCT, XO	HV	13 (6/7)	25	22.8	28 g	White wine	5	120 minutes	50	White bread	PBG
Mettler et al., 2009 (38)	RCT, XO	HV	27 (9/18)	26	22.5	28 g	NI	NI	120 minutes	75	Vanilla milk rice + cinnamon	PBG
Johnston et al., 2010-study 1 (31)	RCT, XO	HV	10 (4/6)	35	27.5	2–20 g	Apple cider	5	120 minutes	0	0 g acetic acid drink	PBG
Johnston et al., 2010-study 2a(31)	RCT, XO	HV	9 (2/7)	50	33.7	20 g	Raspberry	5	120 minutes	75	Placebo (not specified)	PBG
Johnston et al., 2010-study 3 (31)	RCT, XO	HV	10 (2/8)	38	26.3	20 g	Apple cider	5	120 minutes	75	Placebo (not specified)	PBG
Johnston et al., 2010-study 4 (31)	RCT, XO	T2D	9 (4/5)	69	31.4	20 g	Apple cider	5	120 minutes	75	Placebo (not specified)	PBG
Liatis et al., 2010-study a (32)	RCT, XO	T2D	8 (3/5)	57.4	29.8	20 g	Wine (high glycemic index 86/100)	6	120 minutes	51	High glycemic meal	PBG PI
Liatis et al., 2010-studyb (32)	RCT, XO	T2D	8 (4/4)	61.4	30.1	20 g	Wine (low glycemic index 38/100)	6	120 minutes	52	Low glycemic meal	PBG PI
Darzi et al., 2014-study 1 (33)	RCT, XO	HV	16 (3/13)	22.2	22.1	30 g	White wine (palatable)	6	180 minutes	94.5	75 g of sugar-free squash	PBG
Darzi et al., 2014-study 2 (33)	RCT, XO	HV	14 (6/8)	27.5	22.7	30 g	White wine	6	180 minutes	60	Water	PBG
Mitrou et al., 2015-study a (45)	RCT, XO	T2D	11 (4/7)	53.0	25.0	30 ml	NI	6	300 minutes	75	Water	PBG PI muscle glucose uptake
Mitrou et al., 2015-study b (44)	RCT, XO	IGT	8 (4/4)	46.0	30.0	30 ml	Wine	6	300 minutes	75	Water	PBG PI Muscle glucose uptake
Feise and Johnston, 2020-study a (34)	XO	HV	12 (5/7)	22.6	21.2	25 g	Liquid vinegar	5	60 minutes	64	Water	PBG
Feise and Johnston, 2020-study b (34)	XO	HV	12 (5/7)	22.6	21.2	25 g	Vinegar pills	5	60 minutes	64	Water	PBG
Feise and Johnston, 2020-study c (34)	XO	HV	12 (5/7)	22.6	21.2	25 g	Crushed vinegar pills	5	60 minutes	64	Water	PBG
Zhao et al., 2020 (41)	RCT, XO	HV	15 (0/15)	23.6	20.3	30 g	Black rice	5	200 minutes	35	White rice, dried apple	PBG
Chronic												
White and Johnston, 2007 (51)	XO	T2D	11 (4/7)	40–72	29.1	30 ml	Apple cider	0.002	2 days	Usual diet	Water	FBG
Hosseini et al., 2011 (49)	Parallel	T2D	30 (15/15)	30–60	NI	15 ml	Vinegar	NI	4 weeks	Usual diet	Water	FBG HbA1c
Derakhshandeh-Rishehri et al.,2014 (48)	RCT, parallel	HV	72 (32/40)	31.6	25.3; 22.8	21.66 g	Honey	NI	4 weeks	Usual diet	Normal diet	FBG FI HOMA-IR
Ali et al., 2019 (50)	RCT, parallel	T2D	55 (26/29)	30–60	NI	20 ml	Dates	NI	10 weeks	Usual diet	Honey in water	FBG HbA1c
Gheflati et al., 2019 (46)	RCT, parallel	T2D	62 (20/42)	49.5; 52.1	29.0; 28.9	20 ml	Apple	5	8 weeks	Usual diet	Normal diet	FBG FI HOMA-IR QUICKI
Jasbi et al., 2019 (47)	RCT, parallel	HV	45 (41/4)	29.6; 30.1	27.8; 28.5	60 ml	Red wine	6	8 weeks	Usual diet	Apple cider vinegar tablet	FBG FI HOMA-IR

1 Abbreviations: CHO, carbohydrate; FBG, fasting blood glucose; FI, fasting insulin; HbA1c, glycated hemoglobin; HV, healthy volunteers; IGT, impaired glucose tolerance; IR, insulin resistant; NI, no information; PBG, postprandial blood glucose; PI, postprandial insulin; QUICKI, Quantitative Insulin-Sensitivity Check Index; RCT, randomized controlled trial; T2D, type 2 diabetes; XO, crossover.

### Acute interventions

During the literature search, 15 studies within 11 references were identified that investigated the effects of acute vinegar administration on glycemic control. These 15 studies were meta-analyzed (29–31, 33, 34, 38–43) for PBG responses in healthy individuals, with 7 in metabolically compromised individuals (31, 32, 43–45). Five of the healthy volunteer studies (30, 40, 42, 43) and 6 of the nonhealthy volunteer studies (32, 43–45) were also meta-analyzed for the postprandial insulin response.

**FIGURE 5:**
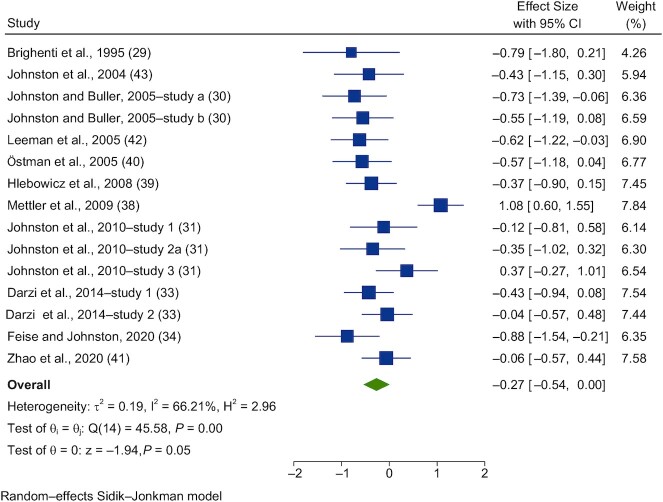
Forest plots for randomized controlled trials of acute vinegar intake on postprandial blood glucose in healthy volunteers. Acute interventions with vinegar had a main effect of −0.27 (95% CI, −0.54 to 0.00; *P* = 0.05) on the postintervention postprandial blood glucose iAUC (*n* = 186) in healthy subjects. A random-effects model was used to calculate standardized mean differences (squares), 95% CIs (horizontal lines), and summary effects (SMD; diamond). The study weight (expressed as a percentage) indicates the relative contribution of an individual study to the overall pooled effect size. Between-study heterogeneity was calculated using the I^2^ statistic. A *P* value ≤ 0.05 was considered statistically significant. When interpreting SMDs (or effect sizes) values <0.40 were categorized as having a small effect size, values 0.40 to 0.70 as having a moderate effect size, and values >0.70 as having a large effect size. Brighenti et al. (29) used acetic acid within vinegar (coingested with 50 g of CHO). Johnston and Buller (30) used (study a) a bagel and juice meal and (study b) chicken teriyaki. Johnston et al. (31) used (study 1) 1 g of acetic acid as vinegar consumed prior to the test meal (bagel + juice); (study 2a) 1 g of acetic acid as vinegar consumed with the test meal; and (study 3) 1 g of acetic acid as vinegar ingested immediately prior to a 75-gram dextrose load in 10 healthy adults. Darzi et al. (33) (study 1) used both a palatable drink with 25 g vinegar vs. control, alongside a mixed breakfast. Darzi et al. (33) (study 2) used a milkshake preload before intake of 30 g of vinegar (containing 6% acetic acid) + 150 g water or the control. Feise and Johnston (34) used 25 g of liquid vinegar (1.25 g acetic acid). Abbreviations: CHO, carbohydrate; iAUC, incremental AUC; SMD, standard mean difference.

**FIGURE 6:**
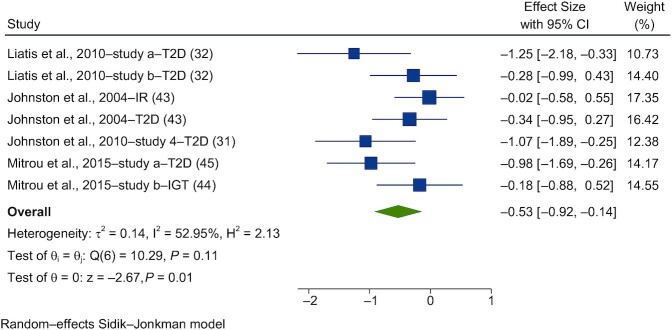
Forest plots for randomized controlled trials of acute vinegar intake on postprandial blood glucose in nonhealthy adults. Acute interventions with vinegar had a main effect of −0.53 (95% CI, −0.92 to −0.14; *P* = 0.01) on the postintervention postprandial blood glucose iAUC (*n* = 67) in nonhealthy subjects. A random-effects model was used to calculate standardized mean differences (squares), 95% CIs (horizontal lines), and summary effects (SMD; diamond). The study weight (expressed as a percentage) indicates the relative contribution of an individual study to the overall pooled effect size. Between-study heterogeneity was calculated using the I^2^ statistic. A *P* value ≤ 0.05 was considered statistically significant. When interpreting SMDs (or effect sizes) values <0.40 were categorized as having a small effect size, values 0.40 to 0.70 as having a moderate effect size, and values >0.70 as having a large effect size. Liatis et al. (32) used (study a) a high-GI meal and (study b) a low-GI meal. Mitrou et al. (44, 45) published results in the (study a) *Journal of Diabetes Research* (Mitrou et al. 45) and (study b) *European Journal of Clinical Nutrition* (Mitrou et al. 44). Abbreviations: GI, glycemic index; iAUC, incremental AUC; IGT, impaired glucose tolerance; IR, insulin resistant; SMD, standard mean difference; T2D, type 2 diabetes.

**FIGURE 7:**
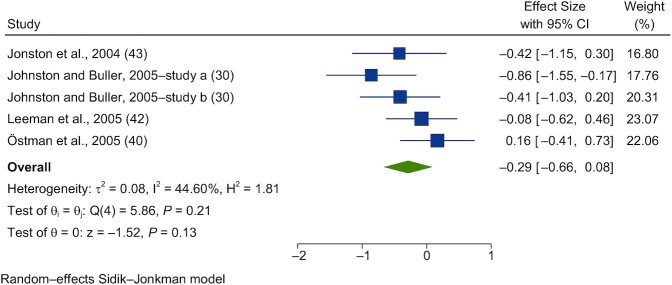
Forest plots for randomized controlled trials of acute vinegar intake on postprandial blood insulin in healthy volunteers. Acute interventions with vinegar had a main effect of −0.29 (95% CI, −0.66 to 0.08; *P* = 0.13) on the postintervention postprandial insulin iAUC (*n* = 55) in healthy subjects. A random-effects model was used to calculate standardized mean differences (squares), 95% CIs (horizontal lines), and summary effects (SMD) (diamond). The study weight (expressed as a percentage) indicates the relative contribution of an individual study to the overall pooled effect size. Between-study heterogeneity was calculated using the I^2^ statistic. A *P* value ≤ 0.05 was considered statistically significant. When interpreting SMDs (or effect sizes) values <0.40 were categorized as having a small effect size, values 0.40 to 0.70 as having a moderate effect size, and values >0.70 as having a large effect size. Johnston and Buller (30) used (study a) a bagel and juice meal and (study b) chicken teriyaki. Abbreviations: iAUC, incremental AUC; SMD, standard mean difference.

**FIGURE 8:**
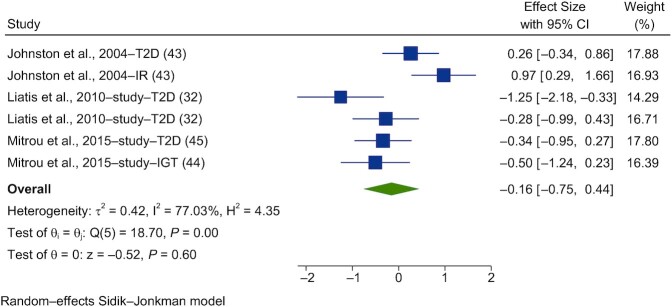
Forest plots for randomized controlled trials of acute vinegar intake on postprandial blood insulin in nonhealthy adults. Acute interventions with vinegar had a main effect of −0.16 (95% CI, −0.75 to 0.44; *P* = 0.60) on the postintervention postprandial blood glucose iAUC (*n* = 58) in nonhealthy adults_._ A random-effects model was used to calculate standardized mean differences (squares), 95% CIs (horizontal lines), and summary effects (SMD; diamond). The study weight (expressed as a percentage) indicates the relative contribution of individual studies to the overall pooled effect size. Between-study heterogeneity was calculated using the I^2^ statistic. A *P* value ≤ 0.05 was considered statistically significant. When interpreting SMDs (or effect sizes) values <0.40 were categorized as having a small effect size, values 0.40 to 0.70 as having a moderate effect size, and values >0.70 as having a large effect size. Liatis et al. (32) used (study a) a high-GI meal and (study b) a low-GI meal. Mitrou et al. (44, 45) published results in the (study a) *Journal of Diabetes Research* (Mitrou et al. 45) and (study b) *European Journal of Clinical Nutrition* (Mitrou et al. 44). Abbreviations: GI, glycemic index; iAUC, incremental AUC; IGT, impaired glucose tolerance; IR, insulin resistant; SMD, standard mean difference; T2D, type 2 diabetes.

Forest plots of the pooled effects of vinegar interventions on PBG and insulin are shown in **Figures 5**–**8**. For blood glucose, a random-effects model showed that acute vinegar interventions had a significant effect on PBG in healthy subjects (SMD = −0.27; 95% CI, −0.54 to 0.00; *n* = 186; Figure 5). The interstudy heterogeneity was significant (I^2^ = 66.2%; *P* = 0.001; 95% CI, 48–82). Acute interventions with vinegar had a significant effect on PBG in subjects with impairments in glucose tolerance (SMD = −0.53; 95% CI, −0.92 to −0.14; *n* = 67; Figure 6). The interstudy heterogeneity was not significant (I^2^ = 53.0%; *P* = 0.11; 95% CI, 0–75).

Acute interventions with vinegar had no significant effect on postprandial insulin (PI) in healthy subjects (SMD = −0.29; 95% CI, −0.66 to 0.08; *n* = 55; Figure 7). The studies had nonsignificant heterogeneity (I^2^ = 44.6%; *P* = 0.21; 95% CI, 0–74). Acute interventions with vinegar had no significant effect on PI in subjects with impaired glucose tolerance (IGT) or T2D (SMD = −0.16; 95% CI, −0.75 to 0.44; *n* = 58; Figure 8). Substantial heterogeneity was seen between the studies included in this meta-analysis (I^2^ = 77.0%; *P* = 0.001; 95% CI, 39–88).

Homogeneity via a Galbraith plot, publication bias via a funnel plot, and a sensitivity analysis via a leave-1-out plot were assessed and are reported in **Supplemental Figures 3–7**.

### Chronic interventions

**FIGURE 9:**
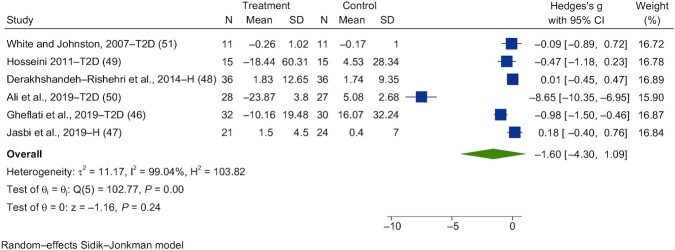
Forest plots for randomized controlled trials of chronic vinegar intake on fasting blood glucose. Chronic interventions with vinegar had a main effect of −1.60 (95% CI, −4.30 to 1.09; *P* = 0.24) on the postintervention fasting blood glucose iAUC (*n* = 143). A random-effects model was used to calculate Hedge's g (squares), 95% CIs (horizontal lines), and summary effects (diamond). The study weight (expressed as a percentage) indicates the relative contribution of an individual study to the overall pooled effect size. Between-study heterogeneity was calculated using the I^2^ statistic. A *P* value ≤ 0.05 was considered statistically significant. Abbreviations: iAUC, incremental AUC; H, healthy volunteers; T2D, type 2 diabetes.

During the literature search, 7 chronic intervention studies using vinegar were identified, and 6 were included in the meta-analysis (46–51) investigating FBG. Chronic interventions with vinegar had no significant effect on fasting glucose (SMD = −1.60; 95% CI, −4.30 to 1.09; *n* = 143; **Figure 9**). The interstudy heterogeneity was significant (I^2^ = 99.0%; *P* = 0.001; 95% CI, 92–97).

**FIGURE 10:**
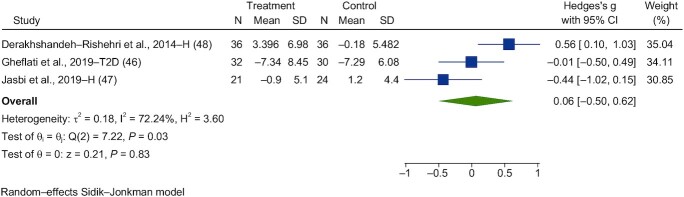
Forest plots for randomized controlled trials of chronic vinegar intake on postprandial blood insulin. Chronic interventions with vinegar had a main effect of 0.06 (95% CI, −0.50 to 0.62; *P* = 0.83) on the postintervention postprandial blood insulin iAUC (*n* = 89)_._ A random-effects model was used to calculate Hedge's g (squares), 95% CIs (horizontal lines), and summary effects (diamond). The study weight (expressed as a percentage) indicates the relative contribution of an individual study to the overall pooled effect size. Between-study heterogeneity was calculated using the I^2^ statistic. A *P* value ≤ 0.05 was considered statistically significant. When interpreting effect sizes, values <0.40 were categorized as having a small effect size, values 0.40 to 0.70 as having a moderate effect size, and values >0.70 as having a large effect size. Abbreviations: iAUC, incremental AUC; H, healthy volunteers; T2D, type 2 diabetes.

Three studies were identified that investigated the effects of chronic vinegar on fasting insulin (FI) responses (46–48). Chronic interventions with vinegar had a significant effect on fasting blood insulin (SMD = 0.06; 95% CI, −0.50 to 0.62; *n* = 89; **Figure 10**). The interstudy heterogeneity was substantial (I^2^ = 72.2%; *P* = 0.03; 95% CI, 6–92).

Homogeneity via a Galbraith plot, publication bias via a funnel plot, and a sensitivity analysis via a leave-1-out plot were assessed and are reported in **Supplemental Figure 8**.

Two eligible studies (49, 50) investigated the effects of chronic vinegar supplementation on HbA1c in individuals with T2D. Patients were supplemented with 15 mL and 20 mL of vinegar for a month or 10 weeks, respectively (49, 50). The authors in both the 15-mL and 20-mL studies reported a significant decrease in HbA1c (of 7% and 9%, respectively), whereas in the placebo group the HbA1c levels decreased by 1% and increased by 2%, respectively (49, 50).

Three studies investigated the degree of insulin resistance, using HOMA-IR, following chronic vinegar intake (46–48). Two of these were investigated in a healthy cohort, showing that while 4 weeks of supplementation of 21 g of vinegar did not result in a significant change compared to the control group (48), 8 weeks of supplementation led to a significant decrease of 8% in HOMA-IR compared to the control groupn (47). One study provided vinegar supplementation in people with T2D for 8 weeks and reported that HOMA-IR, Quantitative Insulin-Sensitivity Check Index (QUICKI), and HOMA-β values were not significantly different compared to those in the control group (46).

### Butyrate

The characteristics of the eligible butyrate studies are summarized in Table 4.

**TABLE 4:** Summary of the butyrate studies’ design, participant, and intervention characteristics and outcomes analyzed^1^

		Participant characteristics				Intervention characteristics				
Reference	Study design	Health status	Sample size (Male/Female)	Age, years, mean	BMI, kg/m^2^, mean	Amount given	Route of administration	Duration	Control	Outcome analyzed
Chronic										
Roshanravanet al., 2017(52)	RCT, Parallel	T2D	30 (10/20)	49	30.3	600 mg Butyrate + 10 g butyrate powder	Oral (capsule)	45 days	Starch	FBG FI PBG PI HOMA-IR HbA1c
Roshanravanet al., 2018 (53)	RCT, Parallel	T2D	30 (10/20)	49	30.3	600 mg Butyrate + 10 g starch powder	Oral (capsule)	45 days	Starch	QUICKI

1 Abbreviations: FBG, fasting blood glucose; FI, fasting insulin; HbA1c, glycated hemoglobin; PBG, postprandial blood glucose; PI, postprandial insulin; QUICKI, Quantitative Insulin-Sensitivity Check Index; RCT, randomized controlled trial; T2D, type 2 diabetes.

### Chronic interventions

Two interventions reported glycemic outcomes following a chronic intervention with butyrate (52, 53). These 2 records described the same study, so a meta-analysis was not possible. In this study, 60 participants with type 2 diabetes were randomized in a parallel design to 4 groups (*n* = 15 in each), in which they had to consume 6 oral capsules (100 mg) and 10 g of powder a day for 45 days. Two of the interventions were assessed in this review. These were sodium butyrate capsules and starch powder (intervention a), and starch capsules and starch powder (control). One publication reported no significant differences in FBG, PBG at 2 hours, FI, HbA1c, and HOMA-IR values postintervention compared to the control group (52). The other reported QUICKI results, which were not significantly different from those in the control group following the 45-day intervention (*P* = 0.137) (53). Compared to the control group, GLP-1 secretion significantly increased following the butyrate intervention (by 22.57 pg/ml; *P* = 0.008) when adjusted for the baseline value, BMI, and blood pressure (52).

### Propionate

The characteristics of the eligible propionate studies are summarized in Table 5.

**TABLE 5:** Summary of the propionate studies’ design, participant, and intervention characteristics and outcomes analyzed^1^

		Participant's characteristics				Intervention characteristics				Glycemic outcome analyzed
Reference	Study design	Health status	Sample size (Male/Female)	Age, years	BMI, kg/m^2^	Amount given	Route of administration	Time-course	Control	
Acute										
Todesco et al., 1991 (55)	RCT, XO	HV	6 (3/3)	32	22.5	3.3. g	Oral bread	180 minutes	Bread	PBG
Laurent et al., 1995 (36)	RCT	HV	6 (3/3)	22	21.2	4 mmol/h	Intragastric (infusion)	300 minutes	Saline	PBG PI
Darwiche et al., (56)	RCT, XO	HV	9 (5/4)	32	23.6	1.85 g	Oral (bread)	125 minutes	Bread	PBG PI
Darzi et al., 2012 (54)	RCT, XO	HV	20 (9/11)	25	23.1	43.8 g	Oral (sourdough bread)	180 minutes	Control bread	PBG PI
Byrne et al., 2016 (57)	RCT, XO	HV	20 (20/0)	52	25.2	10 g	Oral (powder)	360 minutes	Inulin	PBG PI
Chambers et al., 2018 (59)	RCT, XO	HV	18 (9/9)	50	30.5	6.8 g	Oral (tablet)	180 minutes	NaCl 4164 mg	PBG PI
Tirosh et al., 2019 (60)	RCT, XO	HV	14 (9/5)	41	23.7	1.0 g	Oral (calcium propionate)	240 minutes	Placebo (not specified)	PBG PI
Adler et al., 2021 (58)	RCT, XO	HV	27 (12/15)	30	26.7	1.5 g	Oral (calcium propionate)	240 minutes	Calcium carbonate	PBG
Chronic										
Venter et al., 1990 (64)	Paired comparison	HV	20 (0/20)	20–21	18–22.5	7.5 g/d	Oral (capsule)	7 weeks	Calcium phosphate	PBG PI
Todesco et al., 1991 (55)	RCT, XO	HV	6 (3/3)	32	22.5	9.9 g/d	Oral (propionate bread)	1 week	Propionate free white bread	PBG
Chambers et al., 2015 (63)	RCT, Parallel	HV	49 (19/30)	54	32.5	10 g/d	GIT (powder)	24 weeks	Inulin	FI HbA1c HOMA-IR
Pingitore et a., 2017 (69)	RCT, Parallel	HV	49 (19/30)	53	32.5	10 g/d	GIT (sachet)	24 weeks	Inulin	PBG PI
Chambers et al., 2019 (62)	RCT, XO	HV	12 (3/6)	60	29.8	20 g/d	GIT (powder)	42 days	Cellulose	FBG FI HOMA-IR
Byrne et al., 2019 (61)	RCT, XO	HV	21 (9/12)	18–65	60	10 g/d	GIT (bread roll and smoothie)	1 week	Bread	PBG PI

1 Abbreviations: FBG, fasting blood glucose; FI, fasting insulin; GIT, gastrointestinal tract; HbA1c, glycated hemoglobin; HV, healthy volunteers; PBG, postprandial blood glucose; PI, postprandial insulin; RCT, randomized controlled trial; XO, crossover.

### Acute interventions

Eight studies were found to investigate the effects of acute propionate administration on glycemic control. Of these, all were meta-analyzed (36, 54–60) for glucose and 7 were analyzed for insulin (36, 54, 56–60).

**FIGURE 11:**
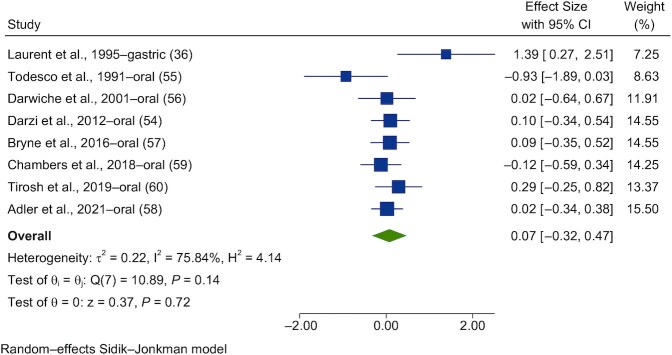
Forest plots for randomized controlled trials of acute propionate on postprandial blood glucose. Acute interventions with propionate had a main effect of 0.07 (95% CI, −0.32 to 0.47; *P* = 0.72) on the postintervention postprandial blood glucose iAUC (*n* = 123). A random-effects model was used to calculate standardized mean differences (squares), 95% CIs (horizontal lines), and summary effects (diamond). The study weight (expressed as a percentage) indicates the relative contribution of an individual study to the overall pooled effect size. Between-study heterogeneity was calculated using the I^2^ statistic. A *P* value ≤ 0.05 was considered statistically significant. When interpreting effect sizes, values <0.40 were categorized as having a small effect size, values 0.40 to 0.70 as having a moderate effect size, and values >0.70 as having a large effect size. Abbreviation: iAUC, incremental AUC.

**FIGURE 12:**
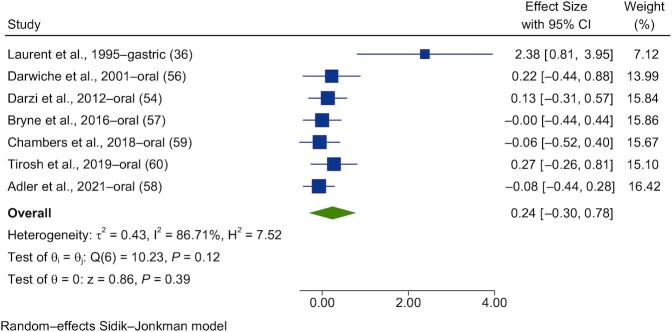
Forest plots for randomized controlled trials of acute propionate on postprandial blood insulin. Chronic interventions with propionate had a main effect of 0.24 (95% CI, −0.30 to 0.78; *P* = 0.39) on the postintervention intervention postprandial blood insulin iAUC_._ (*n* = 117). A random-effects model was used to calculate standardized mean differences (squares), 95% CIs (horizontal lines), and summary effects (diamond). The study weight (expressed as a percentage) indicates the relative contribution of an individual study to the overall pooled effect size. Between-study heterogeneity was calculated using the I^2^ statistic. A *P* value ≤ 0.05 was considered statistically significant. When interpreting effect sizes, values <0.40 were categorized as having a small effect size, values 0.40 to 0.70 as having a moderate effect size, and values >0.70 as having a large effect size. Abbreviation: iAUC, incremental AUC.

Forest plots of the pooled effects of propionate acute interventions on glycemic outcomes are shown in **Figures 11** and **12**. For PBG (Figure 11) and insulin (Figure 12), random-effects models of the acute interventions with propionate had no significant effect [SMD = 0.07 (95% CI, −0.32 to 0.47; *n* = 123) and SMD = 0.24 (95% CI, −0.30 to 0.78; *n* = 117), respectively]. The interstudy heterogeneity was nonsignificant for both PBG and insulin [I^2^ = 75.8% (*P* = 0.14; 95% CI, 0–72) and I^2^ = 86.7% (*P* = 0.12; 95% CI, 0–75), respectively].

Homogeneity via a Galbraith plot, publication bias via a funnel plot, and a sensitivity analysis via a leave-1-out plot were assessed and are reported in **Supplemental Figures 9** and **10**.

### Chronic interventions

Five studies were found to investigate the effects of chronic propionate administration on glycemic control, all of which were meta-analyzed for PBG (55, 61–64). Four studies were identified for PI (61–64). Four studies measured fasting glucose and insulin (61–64).

**FIGURE 13:**
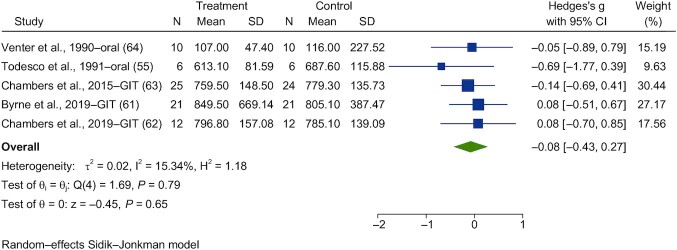
Forest plots for randomized controlled trials of chronic propionate on postprandial blood glucose. Chronic interventions with propionate had a main effect of −0.08 (95% CI, −0.43 to 0.27; *P* = 0.65) on the postintervention postprandial blood glucose iAUC (*n* = 73). Chambers et al. (63) and Pingitore et al. (69) reported the same study, so only Chambers et al. (63) was reported in the meta-analysis. A random-effects model was used to calculate standardized mean differences (squares), 95% CIs (horizontal lines), and summary effects (diamond). The study weight (expressed as a percentage) indicates the relative contribution of an individual study to the overall pooled effect size. Between-study heterogeneity was calculated using the I^2^ statistic. A *P* value ≤ 0.05 was considered statistically significant. When interpreting effect sizes, values <0.40 were categorized as having a small effect size, values 0.40 to 0.70 as having a moderate effect size, and values >0.70 as having a large effect size. Abbreviations: GIT, gastrointestinal tract; iAUC, incremental AUC.

**FIGURE 14:**
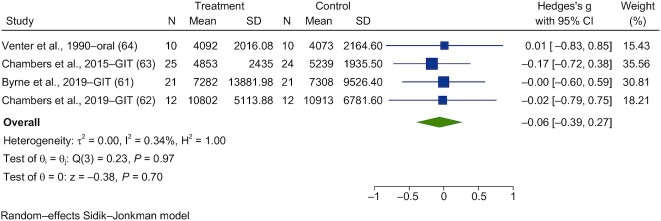
Forest plots for randomized controlled trials of chronic propionate on postprandial blood insulin. Chronic interventions with propionate had a main effect of −0.06 (95% CI, −0.39 to 0.27; *P* = 0.70) on the postintervention intervention postprandial blood insulin iAUC_._ (*n* = 67). A random-effects model was used to calculate standardized mean differences (squares), 95% CIs (horizontal lines), and summary effects (diamond). The study weight (expressed as a percentage) indicates the relative contribution of an individual study to the overall pooled effect size. Between-study heterogeneity was calculated using the I^2^ statistic. A *P* value ≤ 0.05 was considered statistically significant. When interpreting effect sizes, values <0.40 were categorized as having a small effect size, values 0.40 to 0.70 as having a moderate effect size, and values >0.70 as having a large effect size. Abbreviations: GIT, gastrointestinal tract; iAUC, incremental AUC.

**FIGURE 15:**
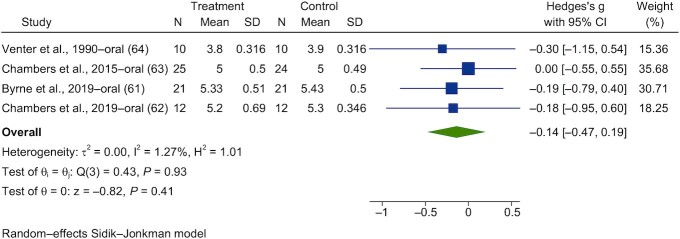
Forest plots for randomized controlled trials of chronic propionate on fasting blood glucose. Chronic interventions with propionate had a main effect of −0.14 (95% CI, −0.47 to 0.19; *P* = 0.41) on the postintervention fasting blood glucose (*n* = 67). A random-effects model was used to calculate standardized mean differences (squares), 95% CIs (horizontal lines), and summary effects (diamond). The study weight (expressed as a percentage) indicates the relative contribution of an individual study to the overall pooled effect size. Between-study heterogeneity was calculated using the I^2^ statistic. A *P* value ≤ 0.05 was considered statistically significant. When interpreting effect sizes, values <0.40 were categorized as having a small effect size, values 0.40 to 0.70 as having a moderate effect size, and values >0.70 as having a large effect size.

**FIGURE 16:**
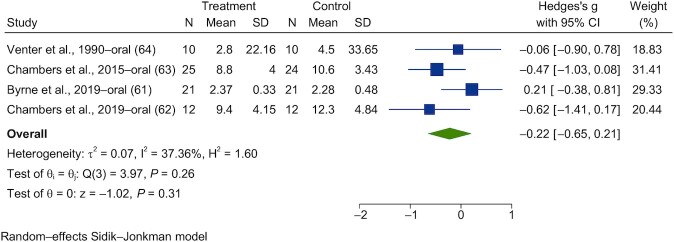
Forest plots for randomized controlled trials of chronic propionate on fasting blood insulin. Chronic interventions with propionate had a main effect of −0.22 (95% CI, −0.65 to 0.21; *P* = 0.31) on the postintervention intervention fasting blood insulin. (*n* = 67). A random-effects model was used to calculate standardized mean differences (squares), 95% CIs (horizontal lines), and summary effects (diamond). The study weight (expressed as a percentage) indicates the relative contribution of an individual study to the overall pooled effect size. Between-study heterogeneity was calculated using the I^2^ statistic. A *P* value ≤ 0.05 was considered statistically significant. When interpreting effect sizes, values <0.40 were categorized as having a small effect size, values 0.40 to 0.70 as having a moderate effect size, and values >0.70 as having a large effect size.

Forest plots are shown in **Figures 13****–****16**. Chronic interventions with propionate had no significant effect on the PBG (Figure 13) or insulin (Figure 14) iAUCs [SMD = −0.08 (95% CI, −0.43 to 0.27; *n* = 73) and SMD = −0.06 (95% CI, −0.39 to 0.27; *n* = 67), respectively]_._ The interstudy heterogeneity was nonsignificant for both PBG and PI [I^2^ = 15.3% (*P* = 0.79; 95% CI, 0–79) and I^2^ = 0.3% (*P* = 0.97; 95% CI, 0–85), respectively]. Chronic interventions with propionate had no significant effect on FBG (Figure 15) or insulin [Figure 16; SMD = −0.14 (95% CI, −0.47 to 0.19; *n* = 67) and SMD = −0.22 (95% CI, −0.65 to 0.21; *n* = 67), respectively]. The interstudy heterogeneity was nonsignificant for both glucose and insulin [I^2^ = 1.3% (*P* = 0.93; 95% CI, 0–85) and I^2^ = 37.4% (*P* = 0.26; 95% CI, 0–88), respectively].

Homogeneity via a Galbraith plot, publication bias via a funnel plot, and a sensitivity analysis via a leave-1-out plot were assessed and are reported in **Supplemental Figures 11–14**.

### Mixed SCFAs

The characteristics of the eligible studies using mixed SCFAs are summarized in Table 6.

**TABLE 6:** Summary of the mixed SCFAs studies design, participant and intervention characteristics and glycemic outcome analyzed^1^

		Participant's characteristics				Intervention characteristics				
Reference	Study design	Health status	Sample size (M/F)	Age, years	BMI, kg/m^2^	Amount given (acetate, propionate, butyrate)	Route of administration	Time-course	Control	Glycemic outcomes analyzed
Mixed SCFAs (acute)										
Wolever et al., 1988 (65)	RCT, XO	HV	6 (3/3)	33.0	NI	Acetate 90 mmol/L + 30 mmol/L Propionate or Acetate 180 mmol/L + 60 mmol/L Propionate	Rectal	120 minutes	Saline	PBG PI
Wolever et al., 1991 (66)	RCT, XO	HV	6	29.0	24.1	Acetate (180 mmol/L) + propionate (60 mmol/L)	Rectal	120 minutes	Saline	PBG PI
Laurent et al., 1995 (36)	RCT, XO	HV	6 (3/3)	22	21.2	Acetate 12 mmol/h Propionate 4 mmol/h	Intragastric (infusion)	300 minutes	Saline	PBG PI
Alamowitch et al., 1996 (67)	RCT, XO	HV	6	26.0	20.9	90 mmol/L (Acetate: propionate: butyrate; 60,25,15 mmol/L)	Ileal	18 hours	Saline	Basal hepatic glucose production insulin sensitivity
Canfora et al., 2017 (68)	RCT, XO	HV	12 (12/0)	36.0	25–35	200 mmol/L (high acetate: 24, 8, 8 mmol/L) (high butyrate: 8, 8, 24 mmol/L) (high propionate: 8, 24, 8 mmol/L)^2^	Rectal	300 minutes	Saline	PBG PI Carbohydrate oxidation

1 Abbreviations: HV, healthy volunteers; NI, no information; PBG, postprandial blood glucose; PI, postprandial insulin; RCT, randomized controlled trial; XO, crossover.

2 The second enema was given 3 hours after the first infusion and given with 75 g of oral glucose load to represent the postprandial state.

### Acute administration

Five studies (36, 65–68) were found to investigate the effects of acute, mixed SCFA administration on glycemic control, all of which were given via the ileum or rectum. In 6 healthy individuals, Wolever and colleagues (65) rectally infused different ratios of SCFA mixtures: acetate at 180 mmol/L and propionate at 60 mmol/L or acetate at 90 mmol/L and propionate at 30 mmol/L and an isotonic saline solution. Neither solution induced a change in blood glucose concentrations. However, the high-acetate mixture led to a decrease in free fatty acids and an increase in total cholesterol and triglyceride concentrations.

The same research team then rectally administered a combination of acetate (180 mmol/L) and propionate (60 mmol/L) to the same population, compared to acetate, propionate, and saline solutions alone (66). The results showed that blood glucose increased by +0.16 mmol/L (*P* ≤ 0.05) and that insulin decreased by −17 pmol/L (*P* ≤ 0.05) with the mixed SCFAs administration compared to acetate alone, independently of changes in glucagon, free fatty acids, and total cholesterol.

Another study in healthy, lean males who received a 18-hour ileal perfusion of SCFAs (acetate at 60 mmol/L, propionate at 25 mmol/L, and butyrate at 15 mmol/L) (67). This was followed by a saline solution at 12 hours. The study showed that mixed SCFA administration did not have an effect on insulin sensitivity, basal hepatic glucose production, or concentrations of triacylglycerol, total cholesterol, and insulin between the 3 conditions (67).

Canfora and team (68) used a rectal infusion of 200 mmol/L of a high-acetate, -propionate, or -butyrate solution or a placebo solution in healthy participants, followed by a 75-g glucose load. There was no differential effect on blood glucose or insulin levels between the 3 SCFA mixtures.

### Certainty of evidence

The certainty of evidence ranged from low to very low for all glycemic outcomes in all SCFAs investigated. GRADE assessments of each outcome can be found in **Tables 7–11**. The certainty of evidence was downgraded due to high risks of bias, imprecision due to small sample sizes and wide CIs, and differences in study methodologies, dose sizes, and study populations. Furthermore, for many of the studies the glycemic response was not the primary outcome, reducing the likelihood that researchers would be able to detect an effect.

**TABLE 7:** GRADE summary of results for acetate compared to placebo or usual treatment for HV and patients with IGT and T2D^1^

	Anticipated absolute effects^2^ (95% CI)					
Outcomes	Risk with placebo or usual treatment	Risk with acetate	Relative effect (95% CI)	Number of participants (studies)	Certainty of the evidence (GRADE)	Comments
Acute postprandial glucose	—	SMD = 0.09 (−0.26 to 0.44)	—	44 (7 RCTs)	2 Very low^3^	The evidence suggests that acetate results in little to no difference in postprandial glucose
Acute postprandial insulin	—	SMD = 0.35 (−0.07 to 0.77)	—	35 (6 RCTs)	2 Very low^3,4^	Acetate may result in little to no difference in postprandial insulin

1 In the GRADE working group grades of evidence, very low certainty indicates we have very little confidence in the effect estimate: the true effect is likely to be substantially different from the estimate of effect. Abbreviations: GRADE, Grading of Recommendations, Assessment, Development, and Evaluation; HV, healthy volunteers; IGT, impaired glucose tolerance; PI, postprandial insulin; RCT, randomized controlled trial; SMD, standardized mean difference; T2D, type 2 diabetes.

2 The risk in the intervention group (and its 95% CI) is based on the assumed risk in the comparison group and the relative effect of the intervention (and its 95% CI).

3 There is a risk of bias due to a lack of blinding and selective outcome reporting. Study populations differed between studies (HV, T2D, hyperinsulinemia, and overweight or obese). The dosage of acetate varied widely, between 1–360 mmol. Results are not generalizable due to the small sample size. Not applicable to wider populationdue to administration methods for acetate. The 95% CIs are very wide.

4 The study populations differed between studies (HV, hyperinsulinemia, and overweight or obese). The dosage of acetate varied widely, between 36–360 mmol. None of the studies had PI as their primary outcome. A possible publication bias was detected by funnel plot.

**TABLE 8:** GRADE summary of results for vinegar compared to placebo or usual treatment for HVs and patients with IGT and T2D^1^

	Anticipated absolute effects^2^ (95% CI)					
Outcomes	Risk with placebo or usual treatment	Risk with vinegar	Relative effect (95% CI)	Number of participants (studies)	Certainty of the evidence (GRADE)	Comments
Acute postprandial glucose (HV)	—	SMD = −0.27 (−0.54 to 0.00)	—	186 (15 RCTs)	2 Very low^3^	Vinegar may reduce or have little to no effect on postprandial glucose in HV, but the evidence is very uncertain
Acute postprandial glucose (T2D, IR, IGT)	—	SMD = −0.53 (−0.92 to 0.14)	—	67 (7 RCTs)	2 Very low^4^^–^^6^	Vinegar may reduce or have little to no effect on postprandial glucose in individuals with T2D, IR, or IGT, but the evidence is very uncertain
Chronic fasting blood glucose (all studies)	—	SMD = −1.60 (−4.30 to 1.09)	—	143 (6 RCTs)	2 Very low^6,7,8^	Vinegar may reduce or have little to no effect on fasting blood glucose, but the evidence is very uncertain
Acute postprandial insulin (HV)	—	SMD = −0.29 (−0.66 to 0.08)	—	55 (5 RCTs)	2 Very low^5,6,8^	Vinegar may reduce or have little to no effect on postprandial insulin in HV, but the evidence is very uncertain
Acute postprandial insulin (T2D, IR, IGT)	—	SMD = −0.16 (−0.75 to 0.44)	—	58 (6 RCTs)	2 Very low^6,9^	Vinegar may reduce or have little to no effect on postprandial insulin in individuals with T2D, IR, or IGT, but the evidence is very uncertain
Chronic fasting insulin (all studies)	—	SMD = 0.06 (−0.50 to 0.62)	—	89 (3 RCTs)	2 Very low^6,10^	Vinegar may increase or have little to no effect on fasting insulin, but the evidence is very uncertain

1 In the GRADE working group grades of evidence, very low certainty indicates we have very little confidence in the effect estimate: the true effect is likely to be substantially different from the estimate of effect. Abbreviations: GRADE, Grading of Recommendations, Assessment, Development, and Evaluation; HV, healthy volunteers; IGT, impaired glucose tolerance; IR, insulin resistant; SMD, standardized mean difference; RCT, randomized controlled trial; T2D, type 2 diabetes.

2 The risk in the intervention group (and its 95% CI) is based on the assumed risk in the comparison group and the relative effect of the intervention (and its 95% CI).

3 A risk of bias arose from a lack of allocation concealment, through the lack of blinding, and in measurement of the outcomes. Different types of vinegar were used, although similar concentrations of acetic acid were documented. There was serious inconsistency (severe heterogeneity I^2^ = 65.1%; *P* = 0.001). The 95% CI is wide.

4 A risk of bias arose from a lack of allocation concealment, due to the lack of blinding, measurement of outcomes, and selective outcome reporting.

5 There is inconsistency due to a very wide 95% CI.

6 There was a possible publication bias detected by funnel plot.

7 There was serious inconsistency (severe heterogeneity I^2^ = 99.0%; *P* = 0.001). There is a very wide 95% CI. The studies were not generalizable, as they were mostly conducted on metabolically unhealthy individuals. Some study comparators contained small amounts of vinegar or acetic acid.

8 A risk of bias arose from a lack of allocation concealment, a lack of blinding, and in the measurement of the outcome.

9 There was serious inconsistency (severe heterogeneity I^2^ = 77.0%; *P* = 0.001). The 95% CI is very wide. There is a risk of bias due to a lack of allocation concealment, lack of blinding, incomplete accounting of patients and outcome events, and selective outcome reporting.

10 A risk of bias arose from a lack of allocation concealment and lack of blinding. The study populations differed between the 3 studies. Different types of vinegar were used. There was serious inconsistency (severe heterogeneity I^2^ = 70.6%; *P* = 0.02).

**TABLE 9:** GRADE summary of results for propionate compared to placebo or usual treatment for HVs and patients with IGT and T2D^1^

	Anticipated absolute effects^2^ (95% CI)					
Outcomes	Risk with placebo or usual treatment	Risk with Propionate	Relative effect (95% CI)	Number of participants (studies)	Certainty of the evidence (GRADE)	Comments
Acute postprandial blood glucose	—	SMD = 0.07 (−0.32 to 0.47)	—	123 (8 RCTs)	2 Very low^3,4^	Propionate may increase or have little to no effect on postprandial glucose (acute), but the evidence is very uncertain
Chronic postprandial blood glucose	—	SMD = −0.08 (−0.43 to 0.27)	—	73 (5 RCTs)	2 Low^4^^–^^6^	The evidence is very uncertain about the effect of propionate on postprandial glucose (chronic)
Chronic fasting glucose	—	SMD = −0.14 (−0.47 to 0.19)	—	67 (4 RCTs)	2 Very low^5^^–^^7^	The evidence suggests that propionate results in little to no difference in fasting glucose (chronic)
Acute postprandial insulin	—	SMD = 0.24 (−0.30 to 0.78)	—	117 (7 RCTs)	2 Very low^3,4^	The evidence is very uncertain about the effect of propionate on postprandial insulin (acute)
Chronic postprandial insulin	—	SMD = −0.06 (−0.39 to 0.27)	—	167 (4 RCTs)	2 Very low^3,6,7^	The evidence suggests that propionate results in little to no difference in postprandial insulin (chronic)
Chronic fasting insulin	—	SMD = −0.22 (−0.65 to 0.21)	—	67 (4 RCTs)	2 Very low^4^^–^^6^	The evidence is very uncertain about the effect of propionate on fasting insulin (chronic)

1 In the GRADE Working Group grades of evidence, low certainty indicates our confidence in the effect estimate is limited: the true effect may be substantially different from the estimate of the effect. Very low certainty indicates we have very little confidence in the effect estimate: the true effect is likely to be substantially different from the estimate of effect. Abbreviations: GRADE, Grading of Recommendations, Assessment, Development, and Evaluation; HV, healthy volunteers; IGT, impaired glucose tolerance; PBG, postprandial blood glucose; RCT, randomized controlled trial; SMD, standardized mean difference; T2D, type 2 diabetes

2 The risk in the intervention group (and its 95% CI) is based on the assumed risk in the comparison group and the relative effect of the intervention (and its 95% CI).

3 The risk of bias mainly arises from the lack of allocation concealment and incomplete accounting of patients and outcome events. Inconsistency may stem from differing interventions (including sodium propionate, calcium propionate, and inulin propionate-ester) and dosages (ranging from <1 g to 9.9 g of the intervention). PBG was the main outcome in 50% of studies.

4 There is a very wide 95% CI.

5 The risk of bias mainly arose from a lack of blinding.

6 A possible publication bias was detected by funnel plot.

7 There is a wide 95% CI.

**TABLE 10:** GRADE Summary of results for mixed SCFAs compared to placebo or usual treatment for HV and patients with IGT and T2D^1^

	Anticipated absolute effects^2^ (95% CI)					
Outcomes	Risk with placebo or usual treatment	Risk with Mixed SCFA	Relative effect (95% CI)	Number of participants (studies)	Certainty of the evidence (GRADE)	Comments
Acute postprandial blood glucose	Not pooled	Not pooled	—	42 (4 RCTs)	2 Low^3^	The evidence suggests that mixed SCFA results in little to no difference in acute postprandial blood glucose. The majority of studies found no significant difference in postprandial blood glucose. One study saw a small, significant increase in blood glucose
Acute postprandial insulin	Not pooled	Not pooled	—	36 (3 RCTs)	2 Low^3^	The evidence suggests that mixed SCFA results in little to no difference in acute postprandial insulin. One study saw a small, significant decrease in insulin secretion after mixed SCFA administration

1 In the GRADE Working Group grades of evidence, low certainty indicates our confidence in the effect estimate is limited: the true effect may be substantially different from the estimate of the effect. Abbreviations: GRADE, Grading of Recommendations, Assessment, Development, and Evaluation; HV, healthy volunteers; IGT, impaired glucose tolerance; RCT, randomized controlled trial; T2D, type 2 diabetes.

2 The risk in the intervention group (and its 95% CI) is based on the assumed risk in the comparison group and the relative effect of the intervention (and its 95% CI).

3 It is difficult to generalize the results due to the small sample sizes. Rectal and gastric infusions mean that the interventions are not very applicable, replicable, or tolerable, reducing transferability. The studies tend to have small sample sizes, increasing imprecision.

**TABLE 11:** GRADE summary of results for butyrate compared to placebo or usual treatment for patients with T2D^1^

	Anticipated absolute effects^2^ (95% CI)					
Outcomes	Risk with placebo or usual treatment	Risk with butyrate	Relative effect (95% CI)	Number of participants (studies)	Certainty of the evidence (GRADE)	Comments
Fasting blood glucose	—	MD = −1.20 (−2.91 to 0.51)	—	30 (1 RCT)	2 Very low^3^	The evidence is very uncertain about the effect of butyrate on fasting blood glucose, as there is only 1 study on chronic butyrate that fit our criteria
Fasting insulin	—	MD = 0.9 (0.57–1.31)	—	30 (1 RCT)	2 Very low^3^	The evidence is very uncertain about the effect of butyrate on fasting insulin, as there was only 1 study investigating butyrate that fit our criteria

1 In the GRADE working group grades of evidence, very low certainty indicates we have very little confidence in the effect estimate: the true effect is likely to be substantially different from the estimate of effect. Abbreviations: GRADE, Grading of Recommendations, Assessment, Development, and Evaluation; MD, mean difference; RCT, randomized controlled trial; T2D, type 2 diabetes.

2 The risk in the intervention group (and its 95% CI) is based on the assumed risk in the comparison group and the relative effect of the intervention (and its 95% CI).

3 The risk of bias was high due to a lack of blinding, incomplete accounting of patients and outcome events, and selective outcome reporting. Publication bias is suspected, as there is only 1 study investigating butyrate, which demonstrates butyrate has a significant effect.

### Confounders of glycemic control

The studies were assessed for controlling for known confounders of glycemic control (body weight and fat change; standard evening meal; whether fiber, strenuous exercise, and alcohol were avoided; and whether an overnight fast was completed prior to the study visit), which are summarized in Supplemental Table 3.

In acute studies, 1 acetate (28) and 3 vinegar (31, 33, 41) studies instructed participants to consume a standard evening meal. Participants in 4 vinegar studies (31, 33, 34, 41) and 2 propionate studies (54, 57), avoided strenuous physical activity. One acetate study (28), 3 vinegar studies (33, 34, 41), and 3 propionate studies (54, 57, 59) discouraged participants from consuming alcohol. Three acetate studies (28, 35, 36), 1 vinegar study (54), 2 propionate studies (36, 54), and 2 mixed vinegar studies (36, 67) prescribed a low-fiber diet before the study visit. All acute studies, excluding 1 which provided no information (29), required participants to fast for more than 6 hours before the study visit.

In chronic studies, 50% of studies accounted for changes in body weight and body fat (52, 53, 55, 61–64, 69). Three chronic propionate studies instructed participants to consume a standard evening meal (62, 63, 69). Four propionate studies instructed participants to avoid strenuous physical activity (61–63, 69). One vinegar study (50) and 4 propionate studies (61–63, 69) instructed participants to abstain from alcohol before the study visit. All studies requested participants to fast for >6 hours before the visit.

### Adverse events

The adverse events (AEs) reported for each intervention arm for each study category were assessed (**Supplemental Table 4**). AE data were not disclosed in all publications.

In acute interventions, no AEs were reported for acetate nor vinegar (35, 41, 44, 45). Two studies reported AEs for propionate interventions: 1 case of nausea was reported (57) and 1 study documented no AEs (54). Two studies reported AEs for mixed SCFA administration, and 1 had up to 6 incidences of belching, in both the intervention and control groups (65), while the other reported no AEs (68).

In chronic interventions with propionate, there were 3 incidences of flatulence when consuming inulin-propionate ester bread (61). Another study with propionate reported 6 cases of nausea, 2 cases of constipation, 1 case of flatulence, and 4 cases of vomiting in the intervention group, although nausea, constipation, and flatulence were also reported in the control group (64). One study with propionate assessed AEs but did not have any to report (63). Three vinegar studies reported AEs (47, 48, 50), but only 1 reported 1 case of nausea, stomachache, and headache (48).

### Compliance

Study adherence to the intervention for each study category was assessed (**Supplemental Table 5**). Withdrawals were defined as participants that dropped out after randomization. One acute study reported a 40% withdrawal rate, because researchers failed to clip the catheter to the colonic mucosa, which meant the SCFA could not be administered (35). Studies acutely supplementing propionate had 100% adherence, whereas chronic interventions had an average withdrawal rate of 12%, and 1 study reported a participant withdrawal due to nausea (64). Studies using chronic butyrate had a 1.7% withdrawal rate due to losses to follow-up (52, 53).

## Discussion

A total of 43 publications with 46 studies and 913 participants were incorporated into our analysis, which showed that acute vinegar administration had a favorable effect on blood glucose in subjects with T2D or IGT and healthy participants. Acute and/or chronic administrations of acetate, vinegar, propionate, butyrate, and mixed SCFAs had no effect on glycemic measures, including FBG, FI, PBG, and PI. A summary of the results of the meta-analyses can be found in **Table 12**.

**TABLE 12:** Summary of the results from the meta-analysis for all subject categories^1^

		Acute	Chronic
Acetate	Glucose	0.09 (−0.26 to 0.44), *n* = 44	No studies found
	Insulin	0.35 (−0.07 to 0.77), *n* = 35	No studies found
Propionate	Glucose	0.07 (−0.32 to 0.47), *n* = 123	PBG, −0.08 (−0.43 to 0.27), *n* = 73 FBG, −0.14 (−0.47 to 0.19), *n* = 67
	Insulin	0.24 (−0.30 to 0.78), *n* = 117	PI, −0.06 (−0.39 to 0.27), *n* = 67 FI, −0.22 (−0.65 to 0.21), *n* = 67
Butyrate	Glucose	No studies found	No meta-analysis possible. See narrative review
	Insulin	No studies found	No meta-analysis possible. See narrative review
Mixed SCFAs	Glucose	No meta-analysis possible. See narrative review	No studies found
	Insulin	No meta-analysis possible. See narrative review	No studies found
Vinegar	Glucose	−0.27 (−0.54 to 0.00), *n* = 186, healthy −0.53 (−0.92 to −0.14), *n* = 67, T2D, IGT	−1.60 (−4.30 to 1.09), *n* = 143
	Insulin	−0.29 (−0.66 to 0.08), *n* = 55, healthy −0.16 (−0.75 to 0.44), *n* = 58, T2D, IGT	0.06 (−0.50 to 0.62), *n* = 89

1 Results are shown as the SMD for acute studies and Hedges’ g for chronic studies (95% CI). A *P* value ≤ 0.05 was significant. Sample sizes are of the number of participants pooled from all studies included in the meta-analysis. Results are shown for all types of participants (healthy and nonhealthy) and for postprandial outcomes, unless otherwise stated. Abbreviations: FBG, fasting blood glucose; FI, fasting insulin; IGT, impaired glucose tolerance; PBG, postprandial blood glucose; PI, postprandial insulin; SMD, standard mean difference; T2D, type 2 diabetes.

### Acute SCFA administration

The effects of acute SCFA administration upon glycemic responses has been explored using acetate, vinegar, propionate, and mixed SCFAs. Doses varied widely, from 12 to 200 mmol/L, and durations ranged from 60 to 1080 minutes. Our findings, which suggest that acute vinegar influences PBG, correspond with a recent meta-analysis (70) in which all participants were pooled and vinegar was shown to reduce glucose and insulin concentrations. This could bode well for future treatments to halt the progression of glycemic deterioration.

Previous studies have suggested that vinegar, propionate, and acetate delay gastric emptying, slowing the rate of glucose absorption from a meal (54, 56, 71, 72). Moreover, vinegar and propionate could inhibit digestive starch enzymes (55, 73), although digestion could be modulated by phenolic compounds rather than by the presence of acetic acid in vinegar (74). Increased fecal bulk was also reported after propionate administration (55), suggesting that undigested starch could be reaching the colon, thereby reducing the glycemic load. Furthermore, acetate and propionate administration promoted gluconeogenesis in rodent studies (75, 76), but gastric administration of propionate was not shown to have any effect (36). One study (66) reported an increase in PBG and a reduction in PI after administration of a propionate-acetate mixture, compared to acetate alone, suggesting that propionate has gluconeogenic potential (77). When acetate is administered via the distal colon (35), it is able to bypass oxidation by the liver and enter systemic circulation via the rectal venous plexus (68). Reductions in circulating free fatty acids have been observed after propionate and acetate administration, which could suggest acetate found in the peripheral circulation may influence fat oxidation (28, 35, 36, 59, 60, 65, 68). However, when acetate was administered via constant gastric infusion in rats in another study, researchers reported increases in lipogenesis and insulin resistance (78).

In the few acute studies that recorded AEs, nausea and belching were reported. The lack of AE reporting makes it difficult to determine the tolerability and safety of SCFA administration. Reported withdrawals were due to methodological issues, noncompliance, unpleasant tastes of interventions, and participant availability. Although there were AEs, the results suggest SCFAs could be tolerated by participants. Unfortunately, this cannot be confirmed, as most studies did not report compliance or withdrawals.

Acute vinegar supplementation was shown to significantly improve PBG in all participants. However, the GRADE certainty of evidence for all acute outcomes, except for mixed SCFAs, was very low. Caution should be taken when interpreting these results, as there is still little to no certainty that acute SCFA administration has any effect on PBG and PI. The low certainty of evidence and lack of significant results in this systematic review stems from variability in the route of administration, dosage, participant health, and sample size, demonstrated by the high heterogeneity of some outcomes [PBG in healthy volunteers (HV) and PI in unhealthy volunteers after vinegar supplementation] and wide I^2^ 95% CIs. Furthermore, publication bias was detected in several outcomes [excluding PBG for vinegar (HV), propionate, and mixed SCFAs and PI for propionate]. Sensitivity analyses also indicated that 3 studies (29, 31, 38) were driving the outcome for acute PBG of vinegar (HV), and 1 study (40) had a significant influence on the outcome of acute PI in HV. These factors influenced the precision and directness of the outcomes, which downgraded the quality of evidence.

### Chronic SCFA administration

The effects of chronic SCFA supplementation in the forms of vinegar, butyrate, and propionate have been investigated. Doses were from 12 to 200 mmol/L, and study durations ranged between 2 and 70 days. All interventions were orally administered, and some studies used alimentary vehicles, such as bread, smoothies, cheese, and dietary fibers. In this review and meta-analysis, chronic supplementation of SCFAs had no significant effect on PBG, PI, FBG, or FI.

Two studies administered more than 15 ml of vinegar per day and reported a significant reduction in FBG (46, 50). Reducing the rate of gastric emptying, via vinegar administration, could have an effect on fasting glucose and insulin concentrations in the long term (65, 71, 72). However, this is yet to be extensively studied. Some studies attributed changes in fasting glycemia to reduced oxidative stress, which can be associated with both the acetic acid and phenolic compounds present in vinegar (79, 80), but only 1 study reported an increase in 2,20-diphenyl-1-picrylhydrazyl, a free radical (46). Oxidative stress is associated with reductions in insulin sensitivity and glycemic deterioration (81).

Chronic sodium butyrate supplementation showed no effect on glycemic measures compared to a starch placebo. Furthermore, a study using ^13^C-labelled butyrate administered in the colon demonstrated that only 2% of butyrate is found systemically (82). At this concentration, it is unlikely that butyrate would exert an effect on metabolically active tissues, such as adipocytes and skeletal muscle, to alter glucose tolerance.

Chambers and colleagues (62) found that fasting insulin, Matsuda index (a measure of insulin sensitivity), and HOMA-IR values improved when propionate was supplemented compared to results in the cellulose group, but were not significantly different to the insulin control group. This perhaps reinforces previous findings suggesting that incubation with propionate inhibits apoptosis and stimulates insulin release in pancreatic β-cells in vitro (69). As inulin can be used for bacterial fermentation, it is not possible to distinguish whether the effect seen was due to increased propionate or overall SCFA production.

An increase in 2-hour postprandial GLP-1 levels was detected following the butyrate intervention (52). Butyrate and other SCFAs act as signaling molecules in the colon, binding to FFAR2 and FFAR3 expressed in the enteroendocrine L-cells and triggering the release of gut hormones such as GLP-1 (83). Chronic propionate administration to adipocytes expressing FFAR2 has been shown to inhibit lipolysis and reduce circulating nonesterified fatty acid concentrations (84). Maintaining low circulating concentrations of nonesterified fatty acids may prevent β-cell dysfunction and peripheral insulin resistance over time (85).

The AEs reported included nausea, flatulence, constipation, vomiting, and belching. Reported withdrawals were due to noncompliance, losses to follow-up, personal reasons, AEs both unrelated and related (vinegar, propionate) to the intervention, consent withdrawal, and the unpleasant taste of the intervention (vinegar). In some cases, withdrawal reasons were not reported; thus, whether they are related to the intervention is not known. These results could suggest that these interventions are not feasible or tolerable for chronic supplementation in a free-living population.

The GRADE certainty of evidence for all chronic outcomes was very low. Caution should be taken when interpreting these results, as there is still little to no certainty that chronic SCFA administration has any effect on PBG, PI, FBG, or FI. High heterogeneity was detected in chronic FBG and PBG responses to vinegar supplementation, and wide 95% CIs were seen for all heterogeneity results, excluding chronic vinegar supplementation and postprandial glucose responses, indicative of lower certainty of the heterogeneity value. Furthermore, publication bias was detected in all chronic outcomes. These factors influenced the precision, consistency, and directness of the outcomes, which downgraded the quality of evidence.

### Future research on SCFAs and glycemic control

Further high-quality, double-blind, randomized controlled trials using standardized study methodology (sites of administration, types of interventions) and powered to detect changes in glycemic responses are required. Future studies should employ gold-standard methodology (e.g., euglycemic hyperinsulinemic clamps) to elucidate the impacts of SCFAs on insulin sensitivity. Studies should also account for and report on glycemic confounders, such as physical activity and body weight changes. Researchers should ensure test foods are blinded and palatable to avoid triggering delayed gastric emptying and confounding results. Further research should attempt to elucidate the effects of SCFA interconversion in the colon by gut microbiota on the host glucose metabolism. Furthermore, tracer and dose-response studies could help to determine optimal concentrations of SCFAs and the roles of SCFAs in different metabolic states. Lastly, improving the understanding of FFAR desensitization during chronic administration of SCFAs may provide insight into how chronic SCFA supplementation could play a therapeutic role in the future.

### Strengths and limitations

One strength of this systematic review is that studies were separated according to categories to increase homogeneity. By separating individuals based on health status, we were able to demonstrate that vinegar supplementation may influence glycemia in metabolically compromised individuals; to our knowledge, this was previously unreported. One limitation of this review is that the paired nature of data from crossover studies was not accounted for, so these studies may be underweighted. Our restrictive inclusion criteria identified a small and heterogeneous pool of studies for each subject category. Some records did not report uniform glycemic measures, such as AUCs, for acute interventions and could not be included in the meta-analysis, which highlights the need to standardize glycemic outcome reporting to aid future meta-analyses. In this review, we analyzed the PBG and PI data via the iAUC, potentially masking any significant differences at specific time points (e.g., first-phase insulin response), which could be informative of metabolic disease progression for subjects with or at risk of T2D.

### Conclusions

The present systematic review and meta-analysis found that acetate, butyrate, and propionate have no effect on glycemic control. Acetic acid, in the form of vinegar, acutely reduced blood glucose levels in adults with T2D and IGT and in healthy adults. This evidence comes from a very limited and heterogeneous number of studies for all categories, with moderate to high risks of bias and low to very low certainties of evidence. Future high-quality research should be focused on investigating the effects of both acute and chronic interventions of SCFAs, with glycemic control measures as the primary outcome in subjects of all health statuses. Such studies should be controlled for confounders that can affect glycemia to ensure that high-quality evidence is produced.

## Supplementary Material

nqac085_Supplemental_File

## Data Availability

Data described in the manuscript, code book, and analytic code will be made available upon request pending application and approval. The protocol for this systematic review and meta-analysis was registered in the international prospective register of systematic reviews (PROSPERO, registration number: CRD42021231115). Supplemental data is available via the “Supplementary data ” link in the online posting of the article at https://academic.oup.com/ajcn/.

## Notes

The Section for Nutrition Research, Department of Metabolism, Digestion and Reproduction is funded by grants from the MRC (Medical Research Council), BBSRC (Biotechnology and Biological Sciences Research Council), and NIHR (National Institute for Health and Care Research); an Integrative Mammalian Biology (IMB) Capacity Building Award; an FP7-HEALTH-2009-241592 EuroCHIP (European Cancer Health Indicator Project-III) grant; and the NIHR Biomedical Research Centre Funding Scheme.

The views expressed are those of the author(s) and not necessarily those of the NHS, the NIHR, or the Department of Health.

Supplemental Tables 1–5 and Supplemental Figures 1–14 are available from the “Supplementary data” link in the online posting of the article and from the same link in the online table of contents at https://academic.oup.com/ajcn/.

AC-M and JEP contributed equally to this work.

Abbreviations used: AE, adverse event; FBG, fasting blood glucose; FFAR, free-fatty-acid receptor; FI, fasting insulin; GLP-1, glucagon-like peptide 1; GRADE, Grading of Recommendations, Assessment, Development, and Evaluation; HbA1c, glycated hemoglobin; HV, healthy volunteers; iAUC, incremental AUC; IGT, impaired glucose tolerance; PBG, postprandial blood glucose; PI, postprandial insulin; PICOS, population, intervention, comparison, outcomes, and study design; PRISMA, Preferred Reporting Items for Systematic Reviews and Meta-Analyses; QUICKI, Quantitative Insulin-Sensitivity Check Index; SMD, standard mean difference; T2D, type 2 diabetes.
