# Supplementary material for: The effects of SCFAs on glycemic control in humans: a systematic review and meta-analysis
Source: Am J Clin Nutr. 2022 Apr 7;116(2):335–61. doi: 10.1093/ajcn/nqac085 (PMC9348993; doi:10.1093/ajcn/nqac085)
Supplement: nqac085_Supplemental_File [file nqac085_supplemental_file.docx]

**The effect of short-chain fatty acids on glycemic control in humans: A systematic review and meta-analysis**

Anna Cherta-Murillo

**Supplemental Online Material**

| **Supplemental Table 1. Search algorithms for each database.** | |
| --- | --- |
| **Database** | **Search Algorithm** |
| PubMed | ("short-chain fatty acids"[Title/Abstract] OR "short-chain fatty acids"[Title/Abstract] OR "short-chain fatty acid"[Title/Abstract] OR "short-chain fatty acid"[Title/Abstract] OR "acetate"[Title/Abstract] OR "acetic acid"[Title/Abstract] OR "vinegar"[Title/Abstract] OR "propionate"[Title/Abstract] OR "propionic acid"[Title/Abstract] OR "butyrate"[Title/Abstract] OR "butyric acid"[Title/Abstract]) AND "clinical trial"[Publication Type] AND (("humans"[Title/Abstract] OR "human"[Title/Abstract] OR "people"[Title/Abstract] OR "adults"[Title/Abstract] OR "adult"[Title/Abstract] OR "subjects"[Title/Abstract] OR "volunteers"[Title/Abstract] OR "participants"[Title/Abstract] OR "patients"[Title/Abstract] OR "men"[Title/Abstract] OR "man"[Title/Abstract]) AND "clinical trial"[Publication Type]) AND ("glucose"[Title/Abstract] OR "glycem*"[Title/Abstract] OR "glycaem*"[Title/Abstract] OR "insulin"[Title/Abstract] OR "insulin*"[Title/Abstract] OR "metabolic"[Title/Abstract] OR "metabolism"[Title/Abstract] OR "homeostatic model assessment"[Title/Abstract] OR "glycated haemoglobin"[Title/Abstract] OR "glycated hemoglobin"[Title/Abstract] OR "glycosylated haemoglobin"[Title/Abstract] OR "a1c"[Title/Abstract] OR "a1c"[Title/Abstract]) AND "clinical trial"[Publication Type] |
| EMBASE | ("short chain fatty acids" or "short-chain fatty acids" or "short chain fatty acid" or "short-chain fatty acid" or acetate or "acetic acid" or "vinegar" or propionate or "propionic acid" or butyrate or "butyric acid").ti. And (humans or human or people or adults or adult or subjects or volunteers or participants or patients or men or man).ti. and (glucose or glycaem* or glycem* or insulin or insulin* or metabolic or metabolism or "homeostatic model assessment" or "glycated haemoglobin" or "glycated hemoglobin" or "glycosylated haemoglobin" or A1c or A1C).ab. |
| Web of Science | TITLE(("short chain fatty acids"  OR "short-chain fatty acids"  OR "short chain fatty acid"  OR "short-chain fatty acid"  OR acetate  OR "acetic acid"  OR "vinegar" OR propionate  OR "propionic acid"  OR butyrate  OR "butyric acid")  AND (humans  OR human  OR people  OR adults  OR adult  OR subjects  OR volunteers  OR participants  OR patients  OR men  OR man)  AND (glucose  OR glycaem*  OR glycem*  OR insulin  OR insulin*  OR metabolic  OR metabolism)) |
| Scopus | TITLE(("short chain fatty acids" or "short-chain fatty acids" or "short chain fatty acid" or "short-chain fatty acid" or acetate or "acetic acid" or "vinegar" or propionate or "propionic acid" or butyrate or "butyric acid") AND (humans or human or people or adults or adult or subjects or volunteers or participants or patients or men or man) AND (glucose or glycaem* or glycem* or insulin or insulin* or metabolic or metabolism)) |
| Cochrane CENTRAL | (short chain fatty acids or short-chain fatty acids or short chain fatty acid or short-chain fatty acid or acetate or acetic acid or vinegar or propionate or propionic acid or butyrate or butyric acid) in Title Abstract Keyword AND (humans or human or people or adults or adult or subjects or volunteers or participants or patients or men or man) in Title Abstract Keyword AND (glucose or glycaem* or glycem* or insulin or insulin* or metabolic or metabolism) |

| **Supplemental Table 2. Excluded papers upon full-text screening** | | |
| --- | --- | --- |
| **Year of Publication** | **Main Author** | **Reason for exclusion** |
| 1990 | Akanji (1) | Population: Chronic renal failure end stage |
| 1994 | Alamowitch (2) | Conference paper |
| 1992 | Amaral (3) | Not available |
| 2001 | Ball (4) | Poster |
| 2019 | Blessy Pusparatha (5) | Control: single-arm |
| 1994 | Bornet (6) | Review |
| 2018 | Bouter (7) | Control: healthy population not intervention |
| 2015 | Canfora (8) | Abstract paper |
| 2015 | Canfora (9) | Conference paper |
| 2019 | Cani (10) | Review |
| 2019 | Chambers (11) | Population: fatty liver disease |
| 2012 | Diaz-Buxo (12) | Outcomes: not glycemic with sufficient detail |
| 2009 | Ebrahimi-Mamaghani* | Not available |
| 2012 | Fernandes (13) | Control: against type population not intervention |
| 2007 | Geng* | Not available |
| 2018 | Johnston (14) | Abstract meeting |
| 2019 | Khatib (15) | Abstract paper |
| 2015 | Kohn (16) | Commentary |
| 1998 | Liljeberg (17) | Results: no error bars |
| 2016 | Lim (18) | Review |
| 2020 | Malkova (19) | Outcomes: not glycemic |
| 2018 | Marzocco (20) | Study design: no control or randomized |
| 2010 | Mitrou(21) | Population: type 1 diabetes |
| 2013 | Nosrati* | Not available in English |
| 2020 | Pagliai (22) | Outcomes: not glycemic |
| 2019 | Petersen (23) | Control: healthy population not intervention |
| 2014 | Petsiou (24) | Review |
| 1967 | Piccardo (25) | Not available in English |
| 2016 | Polyviou (26) | Outcomes: not glycemic |
| 2009 | Salbe (27) | Intervention: administered with octreotide |
| 2016 | Sasaki (28) | Conference paper |
| 2007 | Smith(29) | Outcomes: not glycemic |
| 2003 | Sugiyama (30) | Design: comparators not matched |
| 2013 | Suzuki* | Not available |
| 1993 | Todesco (31) | Control: against type population not intervention |
| 2019 | Vahid (32) | Design: control also contains vinegar |
| 2012 | Van Dijk* | Comment to editor |
| 2014 | Van der Beek (33) | Conference paper |
| 1988 | Yki-Jarvinen (34) | Results: no error bars |
| *citation not available | | |

| **Supplemental Table 3. Control for study design confounders for glycemic outcomes** | | | | | | | |
| --- | --- | --- | --- | --- | --- | --- | --- |
|  | **Weight Change** | **Standard Evening** **Meal** | **Strenuous Exercise** **Avoided** | **Alcohol Avoided** | **Body Fat Change** | **Low fiber diet Prior Study Visit** | **Overnight Fast (>6)** |
| **Acetate (acute)** | | | | | | | |
| Scheppach (1988) (35) | NA | Yes | NI | Yes | NA | Yes (3 days) | Yes (10-12 h) |
| Laurent (1995) (36) | NA | No | NI | NI | NA | Yes (3 days) | Yes (12 h) |
| Freeland (2010) (37) | NA | NI | NI | NI | NA | NI | Yes (12 h) |
| Johnston (2010) (38) | NA | Yes | Yes | No | NA | No | Yes (>10 h) |
| Van der Beek (2016) (39) | NA | No | NI | NI | NA | Yes (3 days) | Yes (16 h) |
| **Vinegar (Acute)** | | | | | | | |
| Brighenti (1995) (40) | NA | No | No | No | No | NA | NI |
| Johnston (2004) (41) | NA | No | No | No | NA | No | Yes (NI) |
| Johnston (2005) (42) | NA | No | No | No | NA | No | Yes (NI) |
| Ostman (2005) (43) | NA | No | No | No | NA | No | Yes (NI) |
| Leeman (2005) (44) | NA | No | No | No | NA | No | Yes (NI) |
| Hlebowicz (2007) (45) | NA | No | No | No | NA | No | Yes (8 h) |
| Mettler (2009) (46) | NA | NI | NI | NI | NA | NI | Yes (10 h) |
| Liatis (2010) (47) | NA | No | No | No | NA | No | Yes (12 h) |
| Johnston (2010) (38) | NA | Yes | Yes | No | NA | No | Yes (>10 h) |
| Darzi (2014) (48) | NA | Yes | Yes | Yes | NA | Yes (evening meal) | Yes (>12 h) |
| Mitrou (2015) (49) | NA | No | No | No | NA | No | Yes (NI) |
| Mitrou (2015) (50) | NA | No | No | No | NA | No | Yes (NI) |
| Zhao (2020) (51) | NA | Yes | Yes | Yes | NA | No | Yes (>12 h) |
| Feise (2020) (52) | NA | No | Yes | Yes | NA | No | Yes (10 h) |
| **Vinegar (Chronic)** | | | | | | | |
| Ali (2019) (53) | No | No | NI | Yes | NI | No | Yes (NI) |
| Gheflati (2019) (54) | No | No | No | No | NI | No | Yes (NI) |
| Hosseini (2011) (55) | NI | No | NI | NI | NI | No | Yes (NI) |
| Jasbi (2019) (56) | No | No | No | No | No | No | Yes (10 h) |
| White (2007) (57) | NA | No | No | No | NA | No | Yes (NI) |
| Derakhshandeh‑Risheheri (2014) (58) | No | No | No | No | No | No | Yes (12 h) |
| **Propionate (Acute)** | | | | | | | |
| Todesco (1991) (59) | Yes | No | NI | NI | NI | NI | Yes (12 h) |
| Laurent (1995) (36) | NA | No | NI | NI | No | Yes (3 days) | Yes (12 h) |
| Darwiche (2001) (60) | NA | No | No | NI | NI | NI | Yes (NI) |
| Darzi (2012) (61) | NA | No | Yes (24 h) | Yes | No | Yes (night before) | Yes (12 h) |
| Byrne (2016) (62) | NA | No | Yes (24 h) | Yes | NI | NI | Yes (NI) |
| Chambers (2018) (63) | No | No | No | Yes | Yes | No | Yes (NI) |
| Tirosh (2019) (64) | NA | NA | NA | NA | NA | NA | Yes (8 h) |
| Adler (2021) (65) | NA | No | NI | NI | NI | No (propionate-free diet for 7 days prior) | Yes (8 h) |
| **Propionate (chronic)** | | | | | | | |
| Venter (1990) (66) | No | No | NI | NI | NI | NI | Yes (12 h) |
| Todesco (1991) (59) | Yes | No | NI | NI | NI | NI | Yes (12 h) |
| Chambers (2015) (67) | Yes | Yes | Yes (24 h) | Yes | Yes | No | Yes (10 h) |
| Pingitore (2017) (68) | Yes | Yes | Yes (24 h) | Yes | Yes | No | Yes (10 h) |
| Byrne (2019) (69) | Yes | No | Yes (24 h) | Yes | Yes | No | Yes (12 h) |
| Chambers (2019) (70) | Yes | Yes | Yes (24 h) | Yes | Yes | No | Yes (10 h) |
| **Butyrate (Chronic)** | | | | | | | |
| Roshanravan (2017) (71) | Yes | No | No | NI | Yes | NI | Yes (12 h) |
| Roshanravan (2018) (72) | Yes | No | No | NI | Yes | NI | Yes (12 h) |
| **Mixed SCFAs (Acute)** | | | | | | | |
| Wolever (1988) (73) | NA | No | No | No | NA | No | Yes (12 h) |
| Wolever (1991) (74) | NA | No | No | No | NA | No | Yes (NI) |
| Laurent (1995) (36) | NA | No | NI | NI | NA | Yes (3 days) | Yes (12 h) |
| Alamowitch (1996) (75) | NA | No | No | No | NA | Yes (meal prior) | Yes (NI) |
| Canfora (2017) (76) | NA | No | No | No | NA | No | Yes (NI) |

| **Supplemental Table 4. Summary of adverse events by study** | | | | | | | | | | | |
| --- | --- | --- | --- | --- | --- | --- | --- | --- | --- | --- | --- |
| **Gastrointestinal (%)** | | | | | | | | | | | |
| **Reference** | **Treatment groups** | **Nausea** | **Constipation** | **Flatulence** | **Vomiting** | **StD** | **GERD** | **Bloating** | **Belching** | **Diarrhea** | **Others** |
| **Acetate (Acute)** | | | | | | | | | | | |
| **Scheppach 1988** | Na Acetate + K Acetate | NI | NI | NI | NI | NI | NI | NI | NI | NI | NI |
| **(Study 1)** (35) | Chloride | NI | NI | NI | IN | NI | NI | NI | NI | NI | NI |
| **Scheppach 1988** | Na Acetate | NI | NI | NI | NI | NI | NI | NI | NI | NI | NI |
| **(Study 3)** (35) | chloride | NI | NI | NI | NI | NI | NI | NI | NI | NI | NI |
| **Laurent (1995)** (36) | Na Acetate | NI | NI | NI | NI | NI | NI | NI | NI | NI | NI |
|  | Saline | NI | NI | NI | NI | NI | NI | NI | NI | NI | NI |
| **Freeland (2010)** (37) | Acetate | NI | NI | NI | NI | NI | NI | NI | NI | NI | NI |
|  | Saline | NI | NI | NI | NI | NI | NI | NI | NI | NI | NI |
| **Johnston (2010) (study 4)** (38) | Na acetate | NI | NI | NI | NI | NI | NI | NI | NI | NI | NI |
|  | Placebo | NI | NI | NI | NI | NI | NI | NI | NI | NI | NI |
| **Van der Beek (2016)** (39) | Na Chloride (proximal) | 0 | 0 | 0 | 0 | 0 | 0 | 0 | 0 | 0 | 0 |
|  | Na Chloride (distal) | 0 | 0 | 0 | 0 | 0 | 0 | 0 | 0 | 0 | 0 |
|  | Na Acetate (proximal) | 0 | 0 | 0 | 0 | 0 | 0 | 0 | 0 | 0 | 0 |
|  | Na Acetate (distal) | 0 | 0 | 0 | 0 | 0 | 0 | 0 | 0 | 0 | 0 |
| **Propionate (Acute)** | | | | | | | | | | | |
| **Todesco (1991)** | Propionate Bread | NI | NI | NI | NI | NI | NI | NI | NI | NI | NI |
| **Study 1** (59) | Plain bread | NI | NI | NI | NI | NI | NI | NI | NI | NI | NI |
| **Darwiche (2001)** (60) | Propionate Bread | NI | NI | NI | NI | NI | NI | NI | NI | NI | NI |
|  | Plain bread | NI | NI | NI | NI | NI | NI | NI | NI | NI | NI |
| **Darzi (2012)** (61) | Propionate Bread | 0 | 0 | 0 | 0 | 0 | 0 | 0 | 0 | 0 | 0 |
|  | Plain bread | 0 | 0 | 0 | 0 | 0 | 0 | 0 | 0 | 0 | 0 |
| **Byrne (2016)** (62) | IPE | 0 | NI | NI | NI | NI | NI | NI | NI | NI | NI |
|  | Inulin | 5 (1) | NI | NI | NI | NI | NI | NI | NI | NI | NI |
| **Chambers (2018)** (63) | IPE | 0 | NI | NI | NI | NI | NI | NI | NI | NI | NI |
|  | Na Chloride | 0 | NI | NI | NI | NI | NI | NI | NI | NI | NI |
| **Tirosh (2019)** (64) | Propionate | NI | NI | NI | NI | NI | NI | NI | NI | NI | NI |
|  | Placebo | NI | NI | NI | NI | NI | NI | NI | NI | NI | NI |
| **Adler (2021)** (65) | Ca Propionate | NI | NI | NI | NI | NI | NI | NI | NI | NI | NI |
|  | Ca Carbonate | NI | NI | NI | NI | NI | NI | NI | NI | NI | NI |
| **Propionate (Chronic)** | | | | | | | | | | | |
| **Venter (1990)** (66) | Na Propionate | 30 (6) | 10 (2) | 5 (1) | 20 (4) | 0 | 0 | 0 | 0 | 0 | 0 |
|  | Control | 15 (3) | 10 (2) | 5 (1) | 0 | 0 | 0 | 0 | 0 | 0 | 0 |
| **Todesco (1991)** | Propionate bread | NI | NI | NI | NI | NI | NI | NI | NI | NI | NI |
| **Study 2** (59) | Plain bread | NI | NI | NI | NI | NI | NI | NI | NI | NI | NI |
| **Laurent (1995)** (36) | Na Acetate | NI | NI | NI | NI | NI | NI | NI | NI | NI | NI |
|  | Saline | NI | NI | NI | NI | NI | NI | NI | NI | NI | NI |
| **Chambers (2015)** (67) | IPE | 0 | NI | NI | NI | NI | NI | NI | NI | NI | NI |
|  | Inulin | 0 | NI | NI | NI | NI | NI | NI | NI | NI | NI |
| **Pingitore (2017)** (68) | IPE | NI | NI | NI | NI | NI | NI | NI | NI | NI | NI |
|  | Inulin | NI | NI | NI | NI | NI | NI | NI | NI | NI | NI |
| **Byrne (2019)** (69) | IPE bread | 0 | 0 | 14.3 (3) | NI | 0 | 0 | 0 | 0 | 0 | 0 |
|  | Plain bread | 0 | 0 | 0 (0) | NI | 0 | 0 | 0 | 0 | 0 | 0 |
| **Chambers (2019)** (70) | IPE | NS | NI | NS | NI | NI | NI | NS | NS | NI | NS |
|  | Cellulose | NS | NI | NS | NI | NI | NI | NS | NS | NI | NS |
| **Butyrate** **(Chronic)** | | | | | | | | | | | |
| **Roshanravan** | Butyrate | NI | NI | NI | NI | NI | NI | NI | NI | NI | NI |
| **(2017)** (71) | Starch | NI | NI | NI | NI | NI | NI | NI | NI | NI | NI |
| **Roshanravan** | Butyrate | NI | NI | NI | NI | NI | NI | NI | NI | NI | NI |
| **(2018)** (72) | Starch | NI | NI | NI | NI | NI | NI | NI | NI | NI | NI |
| **Vinegar (Acute)** | | | | | | | | | | | |
| **Brighenti (1995)** (40) | Acetic acid from vinegar | NI | NI | NI | NI | NI | NI | NI | NI | NI | NI |
|  | Acetate from vinegar | NI | NI | NI | NI | NI | NI | NI | NI | NI | NI |
| **Johnston (2004)** (41) | Sodium chloride | NI | NI | NI | NI | NI | NI | NI | NI | NI | NI |
|  | Vinegar (healthy) | NI | NI | NI | NI | NI | NI | NI | NI | NI | NI |
|  | Placebo (healthy) | NI | NI | NI | NI | NI | NI | NI | NI | NI | NI |
|  | Vinegar (insulin resistant) | NI | NI | NI | NI | NI | NI | NI | NI | NI | NI |
|  | Placebo (insulin resistant) | NI | NI | NI | NI | NI | NI | NI | NI | NI | NI |
|  | Vinegar (T2D) | NI | NI | NI | NI | NI | NI | NI | NI | NI | NI |
|  | Placebo (T2D) | NI | NI | NI | NI | NI | NI | NI | NI | NI | NI |
| **Johnston (2005)** (42) | Apple cider vinegar | NI | NI | NI | NI | NI | NI | NI | NI | NI | NI |
|  | Placebo | NI | NI | NI | NI | NI | NI | NI | NI | NI | NI |
| **Leeman (2005)** (44) | Vinegar with potatoes | NI | NI | NI | NI | NI | NI | NI | NI | NI | NI |
|  | Potatoes | NI | NI | NI | NI | NI | NI | NI | NI | NI | NI |
| **Ostman (2005)** (43) | Acetic acid | NI | NI | NI | NI | NI | NI | NI | NI | NI | NI |
|  | Bread | NI | NI | NI | NI | NI | NI | NI | NI | NI | NI |
| **Hlebowicz (2007)*** (45) | White wine+ White bread | NI | NI | NI | NI | NI | NI | NI | NI | NI | NI |
|  | White bread | NI | NI | NI | NI | NI | NI | NI | NI | NI | NI |
|  | White wine+ Wholemeal bread | NI | NI | NI | NI | NI | NI | NI | NI | NI | NI |
|  | Wholemeal bread | NI | NI | NI | NI | NI | NI | NI | NI | NI | NI |
| **Mettler (2009)** (46) | Vinegar | NI | NI | NI | NI | NI | NI | NI | NI | NI | NI |
|  | Milk rice | NI | NI | NI | NI | NI | NI | NI | NI | NI | NI |
| **Johnston (2010) (study 1)** (38) | Apple cider vinegar | NI | NI | NI | NI | NI | NI | NI | NI | NI | NI |
|  | Placebo | NI | NI | NI | NI | NI | NI | NI | NI | NI | NI |
| **(study 2)** (38) | Red raspberry vinegar | NI | NI | NI | NI | NI | NI | NI | NI | NI | NI |
|  | Placebo | NI | NI | NI | NI | NI | NI | NI | NI | NI | NI |
| **(study 3)** (38) | Apple cider vinegar | NI | NI | NI | NI | NI | NI | NI | NI | NI | NI |
|  | Placebo | NI | NI | NI | NI | NI | NI | NI | NI | NI | NI |
| **(study 4)** (38) | Apple cider vinegar | NI | NI | NI | NI | NI | NI | NI | NI | NI | NI |
|  | Placebo | NI | NI | NI | NI | NI | NI | NI | NI | NI | NI |
| **Liatis (2010)** (47) | Vinegar (high glycemic index meal) | NI | NI | NI | NI | NI | NI | NI | NI | NI | NI |
|  | Placebo (high glycemic index meal) | NI | NI | NI | NI | NI | NI | NI | NI | NI | NI |
|  | Vinegar (low glycemic index meal) | NI | NI | NI | NI | NI | NI | NI | NI | NI | NI |
|  | Placebo (low glycemic index meal) | NI | NI | NI | NI | NI | NI | NI | NI | NI | NI |
| **Darzi (2014)** (48) | White wine | NI | NI | NI | NI | NI | NI | NI | NI | NI | NI |
|  | Water | NI | NI | NI | NI | NI | NI | NI | NI | NI | NI |
| **Mitrou (2015)** (49) | White wine vinegar | 0 | 0 | 0 | 0 | 0 | 0 | 0 | 0 | 0 | 0 |
|  | Water | 0 | 0 | 0 | 0 | 0 | 0 | 0 | 0 | 0 | 0 |
| **Mitrou (2015)** (50) | White wine vinegar | 0 | NI | NI | 0 | NI | NI | NI | NI | NI | NI |
|  | Water | 0 | NI | NI | 0 | NI | NI | NI | NI | NI | NI |
| **Feise (2020)** (52) | Control | NI | NI | NI | NI | NI | NI | NI | NI | NI | NI |
|  | Vinegar | NI | NI | NI | NI | NI | NI | NI | NI | NI | NI |
|  | Acetic acid pill | NI | NI | NI | NI | NI | NI | NI | NI | NI | NI |
|  | Acetic acid crushed pill | NI | NI | NI | NI | NI | NI | NI | NI | NI | NI |
| **Zhao (2020)** (51) | White rice vinegar | 0 | 0 | 0 | 0 | 0 | 0 | 0 | 0 | 0 | 0 |
|  | White rice | 0 | 0 | 0 | 0 | 0 | 0 | 0 | 0 | 0 | 0 |
| **Vinegar (chronic)** | | | | | | | | | | | |
| **White (2007)** (57) | Apple cider vinegar | NI | NI | NI | NI | NI | NI | NI | NI | NI | NI |
|  | Water | NI | NI | NI | NI | NI | NI | NI | NI | NI | NI |
| **Hosseini (2011)** (55) | Vinegar | NI | NI | NI | NI | NI | NI | NI | NI | NI | NI |
|  | Water | NI | NI | NI | NI | NI | NI | NI | NI | NI | NI |
| **Derakhshandeh-Rishehri (2014)**** (58) | Honey vinegar syrup | 1.63 (1) | NI | NI | NI | NI | NI | NI | NI | NI | 1.63 (1), Headache  1.63 (1), Stomach ache |
|  | Placebo | NI | NI | NI | NI | NI | NI | NI | NI | NI | NI |
| **Ali (2019)** (53) | Date vinegar | 0 | 0 | 0 | 0 | 0 | 0 | 0 | 0 | 0 | 0 |
|  | Placebo | 0 | 0 | 0 | 0 | 0 | 0 | 0 | 0 | 0 | 0 |
| **Gheflati (2019)** (54) | Apple cider vinegar | NI | NI | NI | NI | NI | NI | NI | NI | NI | NI |
|  | Placebo | NI | NI | NI | NI | NI | NI | NI | NI | NI | NI |
| **Jasbi (2019)** (56) | Red wine vinegar | 0 | 0 | 0 | 0 | 0 | 0 | 0 | 0 | 0 | 0 |
|  | Placebo tablets | 0 | 0 | 0 | 0 | 0 | 0 | 0 | 0 | 0 | 0 |
| **Mixed SCFA** | | | | | | | | | | | |
| **Wolever (1988)** (73) | Acetate (90 mmol) + Propionate (30mmol) | NI | NI | NI | NI | NI | NI | NI | 66.6 (4) | NI | NI |
|  | Acetate (180 mmol) + Propionate (60mmol) | NI | NI | NI | NI | NI | NI | NI | 100 (6) | NI | NI |
|  | Isotonic Saline | NI | NI | NI | NI | NI | NI | NI | 83.3 (5) | NI | NI |
| **Wolever (1991)** (74) | Acetate (180 mmol) + propionate (60 mmol) | NI | NI | NI | NI | NI | NI | NI | NI | NI | NI |
|  | Saline solution | NI | NI | NI | NI | NI | NI | NI | NI | NI | NI |
| **Laurent (1995)** (36) | Acetate + Propionate | NI | NI | NI | NI | NI | NI | NI | NI | NI | NI |
|  | Saline | NI | NI | NI | NI | NI | NI | NI | NI | NI | NI |
| **Alamowitch (1996)** (75) | SCFA mixture [Acetate (60%) + propionate (25%) + butyrate (15%)] for 18 h | NI | NI | NI | NI | NI | NI | NI | NI | NI | NI |
|  | SCFA mixture for 12 h + saline solution 6h | NI | NI | NI | NI | NI | NI | NI | NI | NI | NI |
|  | Saline solution | NI | NI | NI | NI | NI | NI | NI | NI | NI | NI |
| **Canfora (2017)** (76) | SCFA mixture high in acetate (200 mmol/L) | 0 | 0 | 0 | 0 | 0 | 0 | 0 | 0 | 0 | 0 |
|  | SCFA mixture high in propionate (200 mmol/L) | 0 | 0 | 0 | 0 | 0 | 0 | 0 | 0 | 0 | 0 |
|  | SCFA mixture high in butyrate (200 mmol/L) | 0 | 0 | 0 | 0 | 0 | 0 | 0 | 0 | 0 | 0 |
|  | Placebo | 0 | 0 | 0 | 0 | 0 | 0 | 0 | 0 | 0 | 0 |
| *Only mention: “If the subject reported gastrointestinal symptoms (diarrhea or constipation) on the study day, the examination was postponed”, but no mention of participants suffering these symptoms. **Side effects appeared after 15 days.  0, none; Ca, calcium, Na, sodium, NI, no information, NS, not significantly different between groups, (), participant number; IPE, inulin propionate ester; GERD, gastro-oesophageal reflux disease; StD, stomach discomfort. | | | | | | | | | | | |

| **Supplemental Table 5. Summary of adherence by study** | | |
| --- | --- | --- |
|  | **Percentage withdrawal (n)** | **Reasons for withdrawal** |
| Acetate (Acute) | | |
| Scheppach (1988) (35) | 0 | NA |
| Laurent (1995) (36) | 0 | NA |
| Freeland (2010) (37) | 0 | NA |
| Johnston (2010) (38) |  |  |
| Van der Beek (2016) (39) | 40 (4) | Two failed attempts to clip the catheter to the colonic mucosa on two separate days |
| **Propionate** **(Acute)** | | |
| Todesco (1991) (59) | 0 | NA |
| Laurent (1995) (36) | 0 | NA |
| Darwiche (2001) (60) | 0 | NA |
| Darzi (2012) (61) | 0 | NA |
| Byrne (2016) (62) | 0 | NA |
| Chambers (2018) (63) | 0 | NA |
| Tirosh (2019) (64) | 0 | NA |
| Adler (2021) (65) | 3.44 (1) | Unable to follow protocol (n=1) |
| **Propionate (Chronic)** | | |
| Venter (1990) (66) | 5 (1) | Nausea |
| Todesco (1991) (59) | 0 | NA |
| Chambers (2015) (67) | 18.3 (11) | Did not complete the 24-week supplementation |
| Pingitore (2017) (68) | 0 | NA |
| Byrne (2019) (69) | 0 | NA |
| Chambers (2019) (70) | 0 | NA |
| **Butyrate** **(Chronic)** | | |
| Roshanravan (2017) (71) | 1.7 (1) | Lost to follow up |
| Roshanravan (2018) (72) | 1.7 (1) | Lost to follow up |
| **Vinegar (Acute)** | | |
| Brighenti (1995) (40) | 0 | NA |
| Johnston (2004) (41) | 0 | NA |
| Johnston (2005) (42) | 0 | NA |
| Ostman (2005) (43) | 0 | NA |
| Leeman (2005) (44) | 0 | NA |
| Hlebowicz (2007) (45) | 0 | NA |
| Mettler (2009) (46) | 0 | NA |
| **Johnston (2010) (study 1)** (38) | 0 | NA |
| **(study 2) (**4) | 0 | NA |
| **(study 3) (**4) | 0 | NA |
| **(study 4) (**4) | 0 | NA |
| **Liatis (2010)** (47) | 0 | NA |
| **Darzi (2014)** (48) | 0 | NA |
| **Mitrou (2015)**(49) | 0 | NA |
| **Mitrou (2015)** (50) | 0 | NA |
| Zhao (2020) (51) | 17 (3) | Time conflicts (n=2) and dislike of vinegar (n=1) |
| Feise (2020) (52) | 0 | NA |
| **Vinegar (Chronic)** | | |
| **White (2007)** (57) | 0 | NA |
| **Hosseini (2011)** (55) | 0 | NA |
| **Derakhshandeh-Rishehri (2014)** (58) | 84.7 (11) | Personal reasons (n=5), viral infection and drug therapy (n=4), seasonal allerglycemic indexes (n=1), adverse effects (nausea, stomachache, headache) (n=1) |
| **Ali (2019)** (53) | 8.33 (5) | Protocol variance (n=3), consent withdrawal (n=2) |
| **Gheflati (2019)** (54) | 11 (8) | Vinegar intolerance, not intention to continue the trial, medication changes (no number of participants provided for each withdrawal reason) |
| **Jasbi (2019)** (56) | 29 (19) | Dislike vinegar taste (n=2), no mention of withdrawal reasons for the rest of the participants. |
| **Mixed SCFA** | | |
| Wolever (1988) (73) | 0 | NA |
| Wolever (1991) (74) | 0 | NA |
| Laurent (1995) (36) | 0 | NA |
| Alamowitch (1996) (75) | 0 | NA |
| Canfora (2017) (76) | 7.6 (1) | The participant decided to withdraw before starting. |
| NA, not applicable. | | |


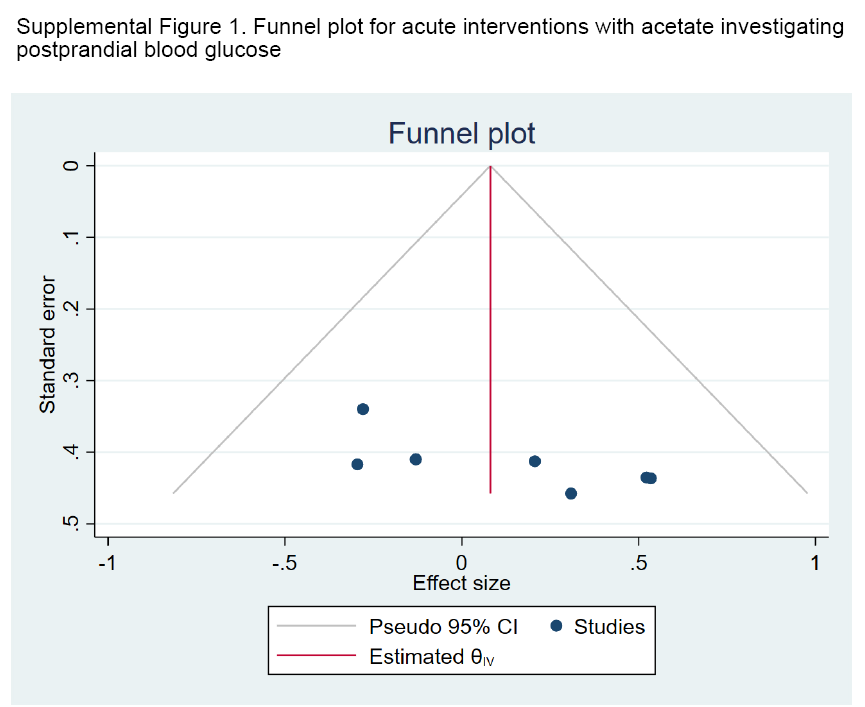


**Supplemental Figure 1.** **Funnel plots to assess publication bias in randomized controlled trials of acute acetate on postprandial blood glucose.** n= 44. CI, confidence interval.


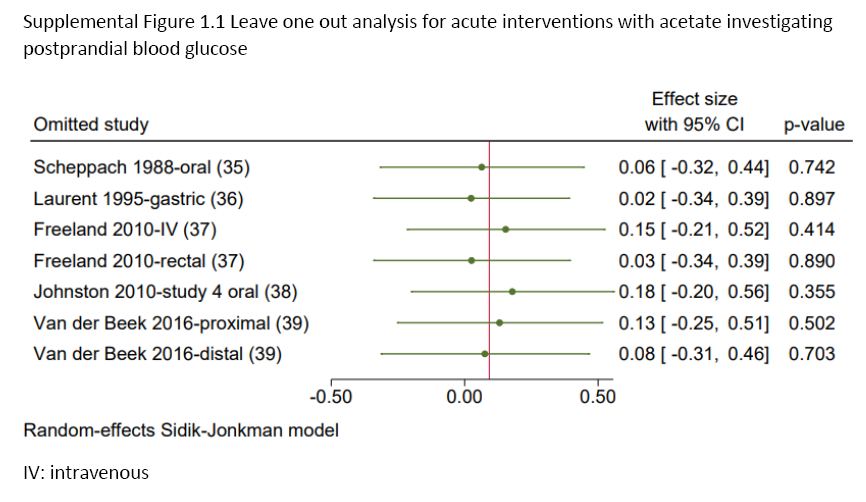


**Supplemental Figure 1.1.** **Leave one out sensitivity analysis for randomized controlled trials of acute acetate on postprandial blood glucose.** Random-effects Sidik-Johnkman model. n= 44. P-value ≤0.05 was considered statistically significant. CI, confidence interval, IV, intravenous.


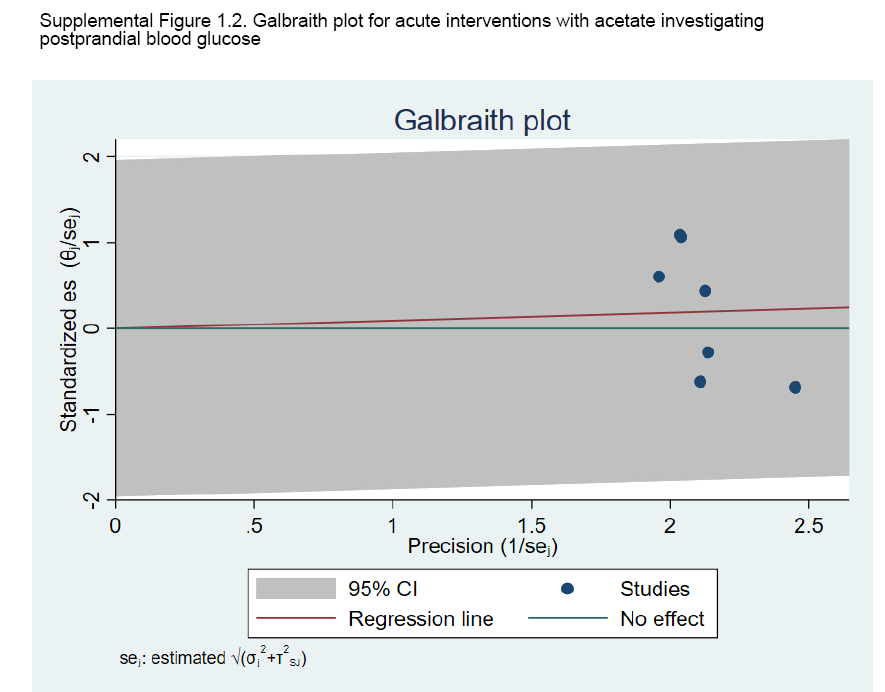
**Supplemental Figure 1.2.** **Galbriath plot to assess for heterogeneity for randomized controlled trials of acute acetate on postprandial blood glucose.** n= 44. CI, confidence interval.


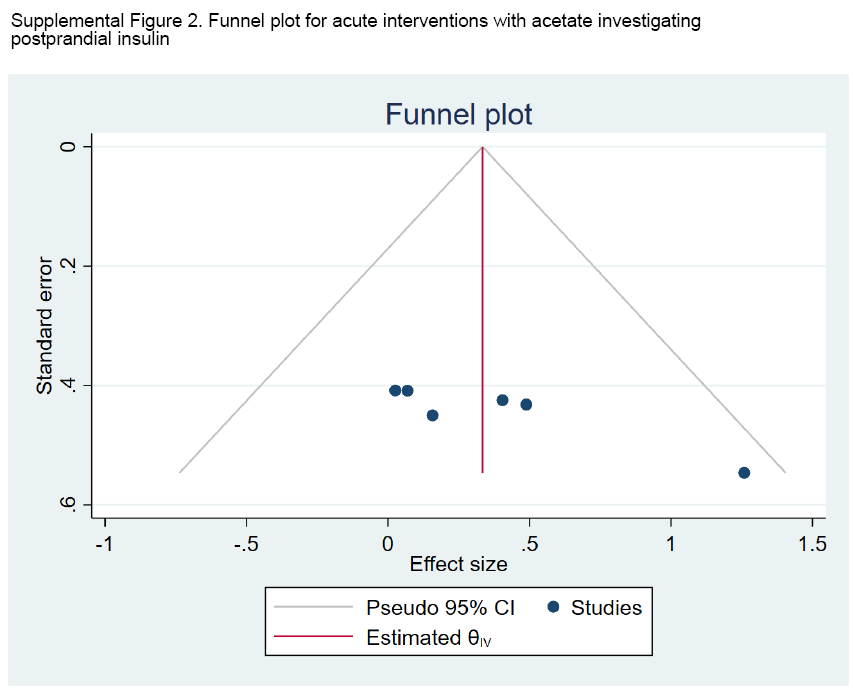
**Supplemental Figure 2.** **Funnel plots to assess publication bias in randomized controlled trials of acute acetate on postprandial blood insulin.** n=35. CI, confidence interval.


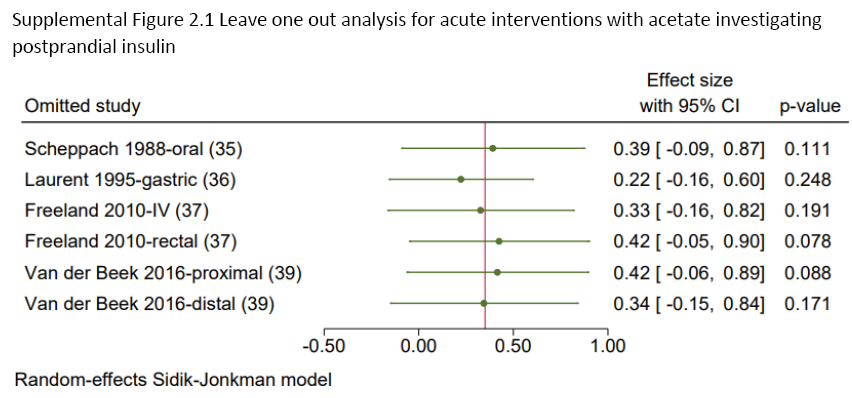


**Supplemental Figure 2.1.** **Leave one out sensitivity analysis for randomized controlled trials of acute acetate on postprandial blood insulin.** n=35. Random-effects Sidik-Johnkman model. n=35. P-value ≤0.05 was considered statistically significant. CI, confidence interval, IV, intravenous.


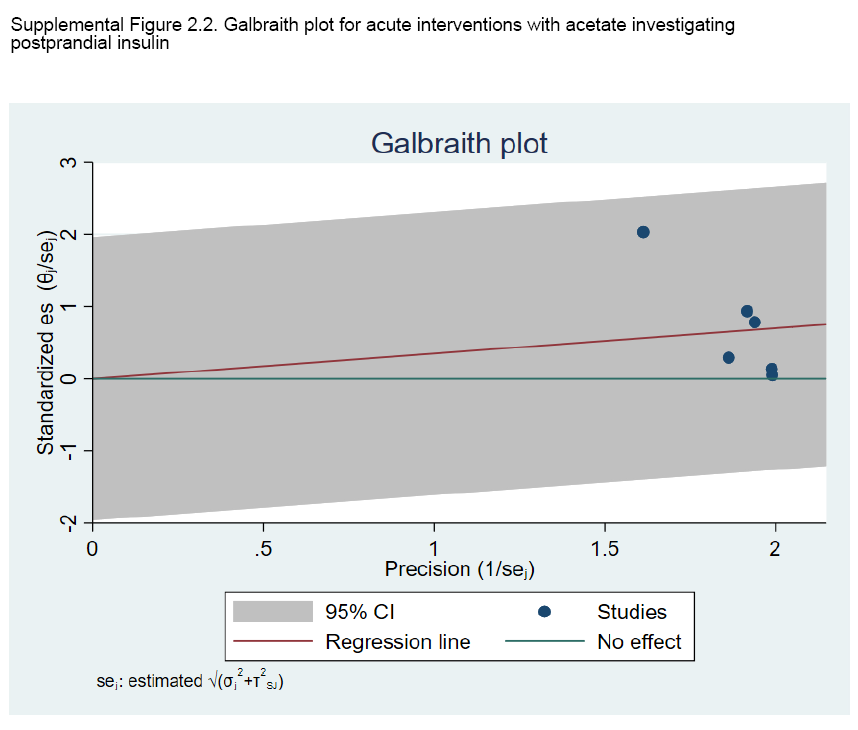
**Supplemental Figure 2.2.** **Galbriath plot to assess for heterogeneity for randomized controlled trials of acute acetate on postprandial blood insulin.** n=35. CI, confidence interval.


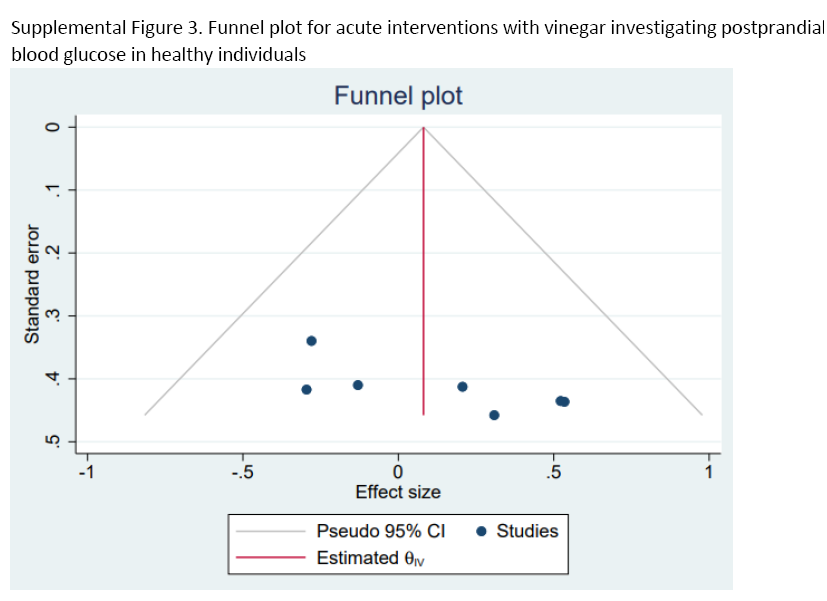


**Supplemental Figure 3.** **Funnel plots to assess publication bias in acute vinegar intake on postprandial blood glucose in healthy volunteers.** n=186. CI, confidence interval.


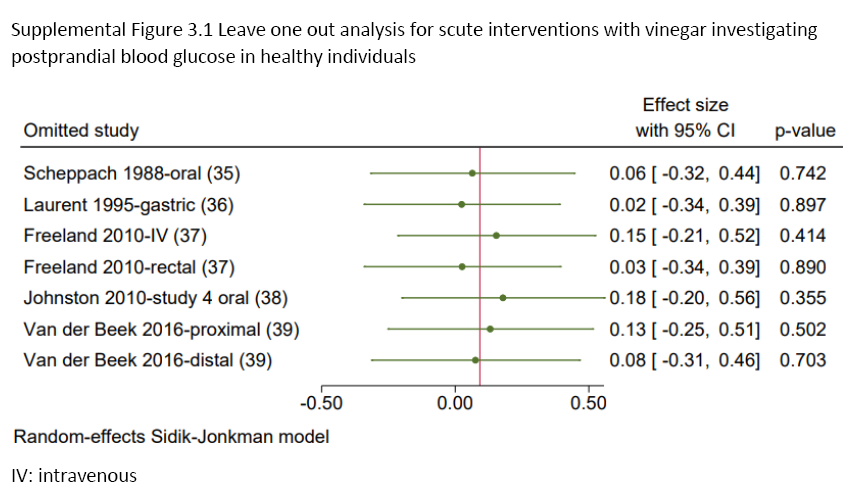
**Supplemental Figure 3.1.** **Leave one out sensitivity analysis for acute vinegar intake on postprandial blood glucose in healthy volunteers.** n=186.Random-effects Sidik-Johnkman model. n=35. P-value ≤0.05 was considered statistically significant. CI, confidence interval, IV, intravenous.


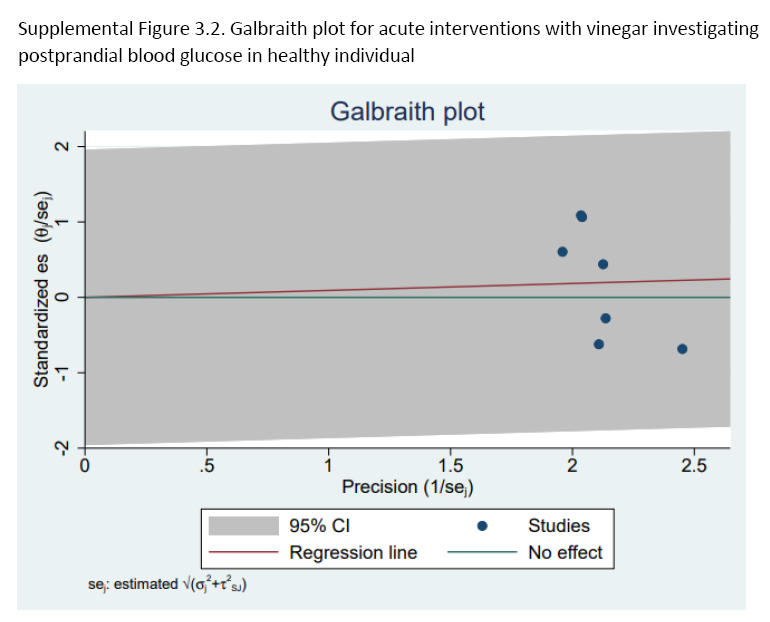
**Supplemental Figure 3.2.** **Galbriath plot to assess for heterogeneity for acute vinegar intake on postprandial blood glucose in healthy volunteers.** n=186. CI, confidence interval.


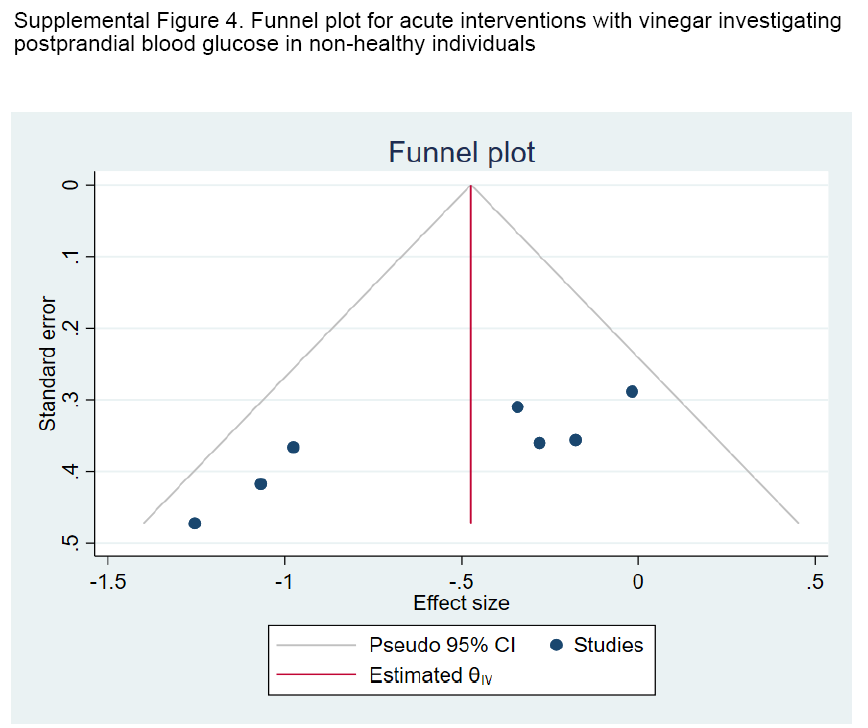


**Supplemental Figure 4.** **Funnel plots to assess publication bias in acute vinegar intake on postprandial blood glucose in non-healthy adults.** n=67. CI, confidence interval.


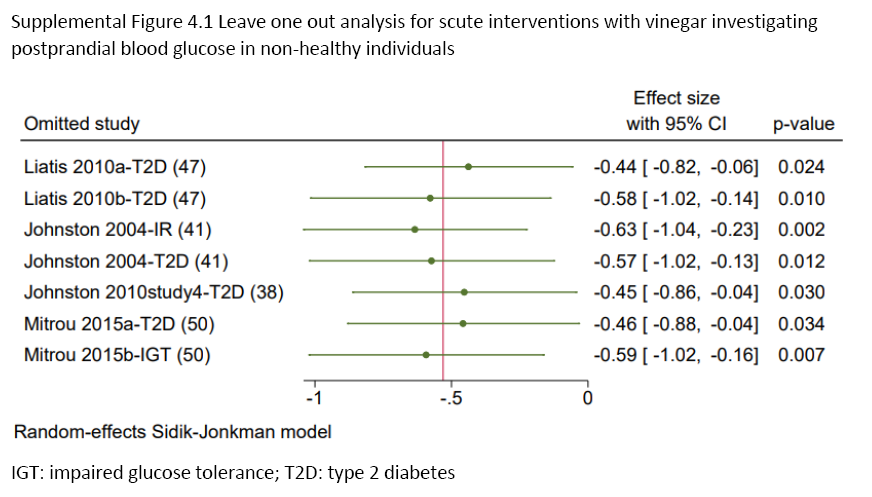


**Supplemental Figure 4.1.** **Leave one out sensitivity analysis for acute vinegar intake on postprandial blood glucose in non-healthy adults.** n=67. Random-effects Sidik-Johnkman model. P-value ≤0.05 was considered statistically significant. CI, confidence interval, IGT, impaired glucose tolerance, T2D, type 2 diabetes


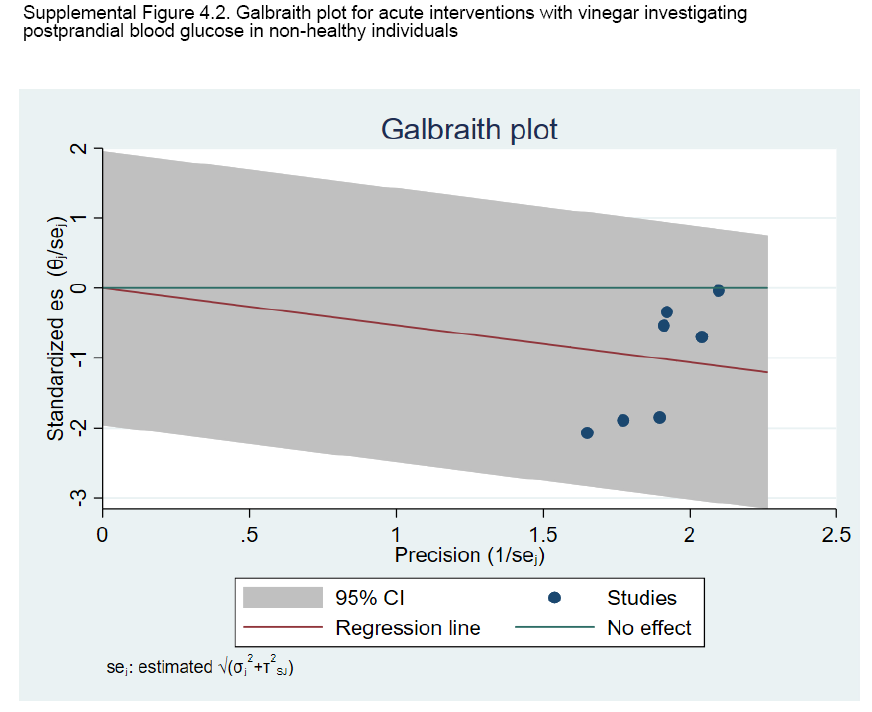


**Supplemental Figure 4.2.** **Galbriath plot to assess for acute vinegar intake on postprandial blood glucose in non-healthy adults.** n=67. CI, confidence interval.


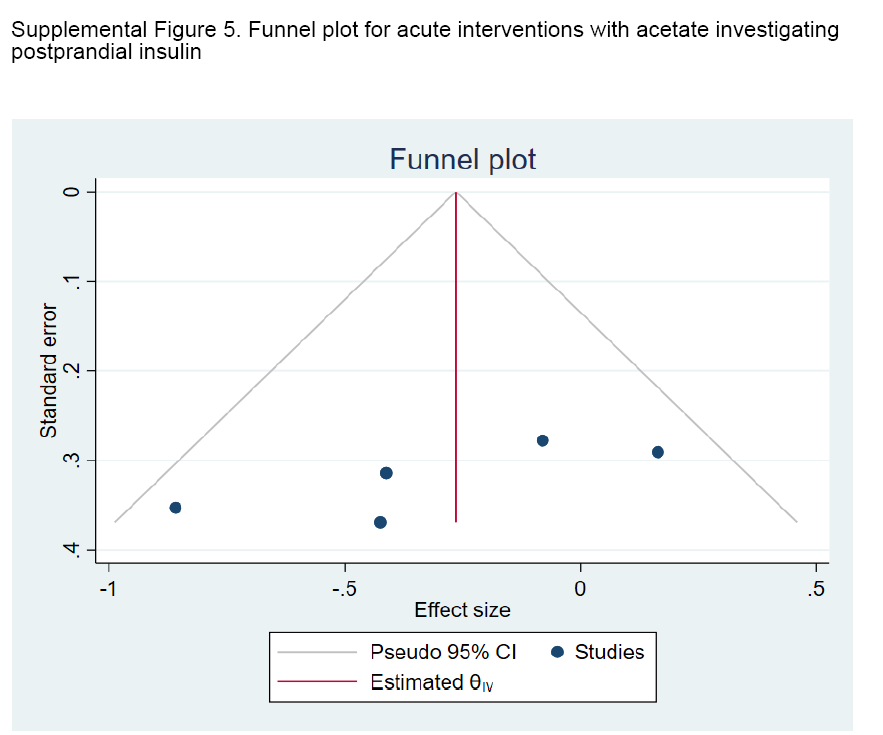


**Supplemental Figure 5.** **Funnel plots to assess publication bias in acute vinegar intake on postprandial blood insulin in healthy volunteers.** n=55. CI, confidence interval.


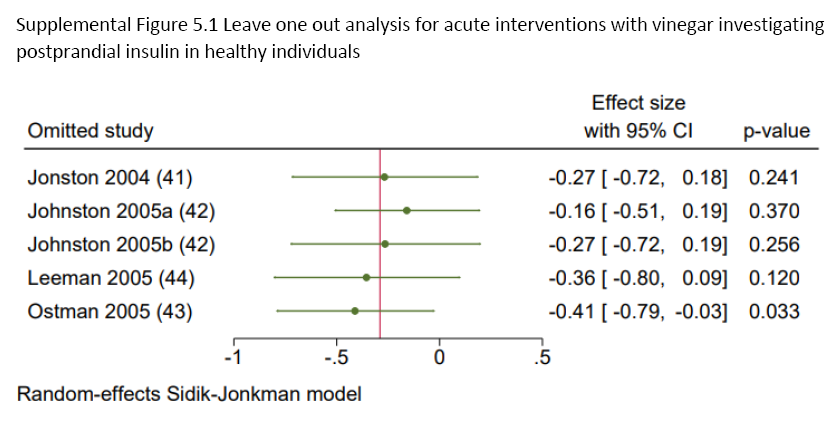


**Supplemental Figure 5.1.** **Leave one out sensitivity analysis for acute vinegar intake on postprandial blood insulin in healthy volunteers.** n=55. Random-effects Sidik-Johnkman model. P-value ≤0.05 was considered statistically significant. CI, confidence interval.

**
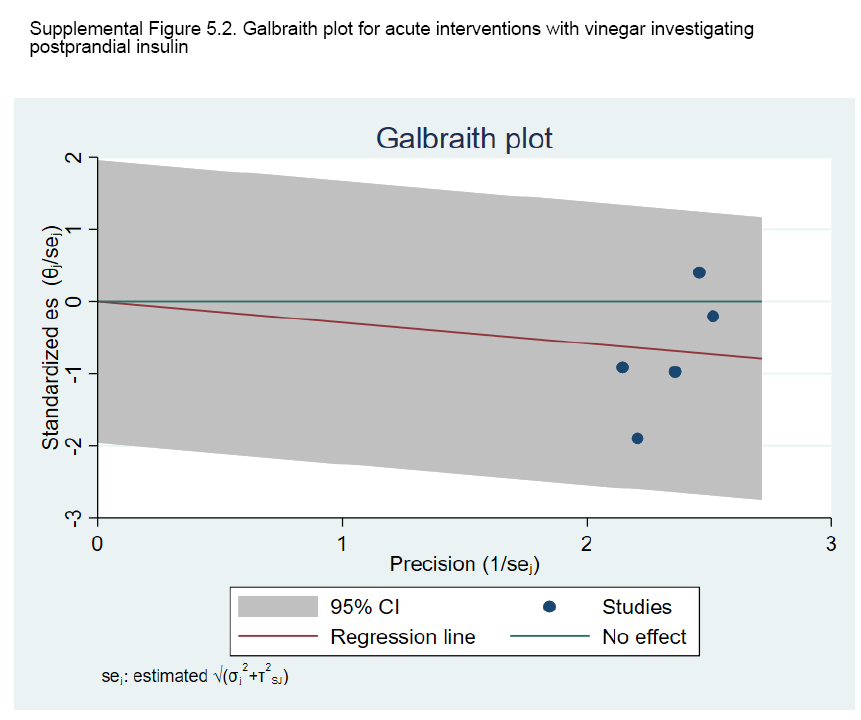
 Supplemental Figure 5.2.** **Galbriath plot to assess for acute vinegar intake on postprandial blood insulin in healthy volunteers.** n=55. CI, confidence interval.

**
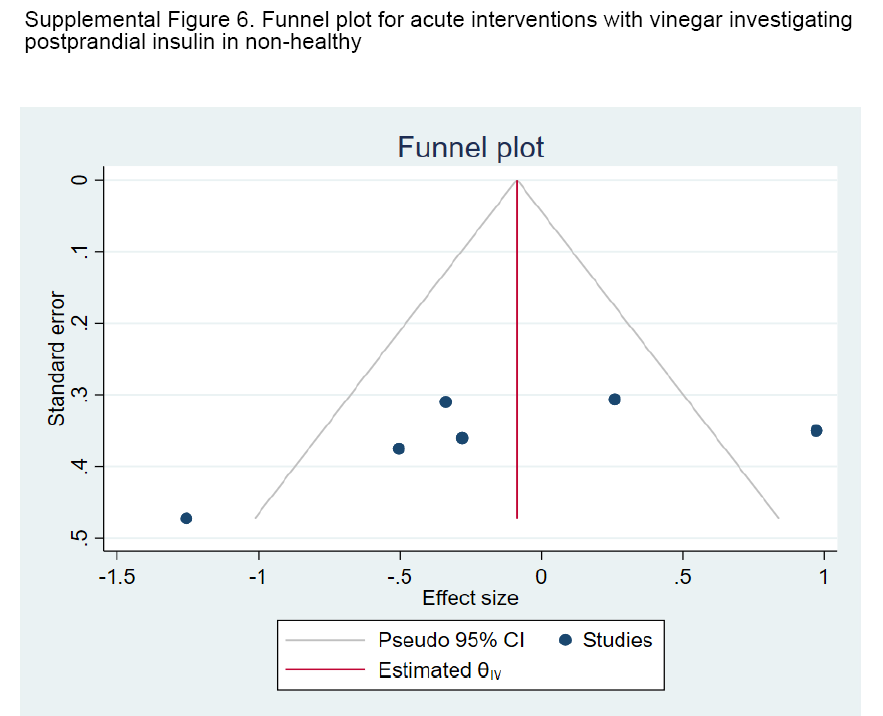
** **Supplemental Figure 6.** **Funnel plots to assess publication bias in acute vinegar intake on postprandial blood insulin in non-healthy adults.** n=58. CI, confidence interval.

**
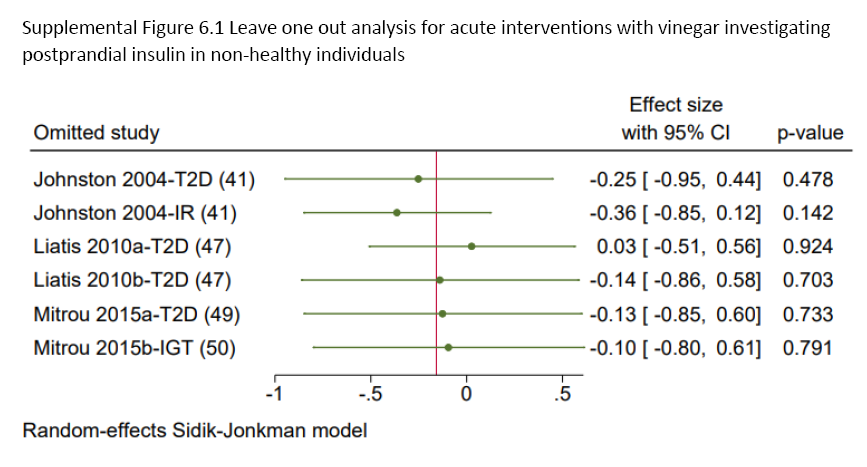
 Supplemental Figure 6.1.** **Leave one out sensitivity analysis for acute vinegar intake on postprandial blood insulin in non-healthy adults.** n=58. Random-effects Sidik-Johnkman model. P-value≤0.05 was considered statistically significant. CI, confidence interval.


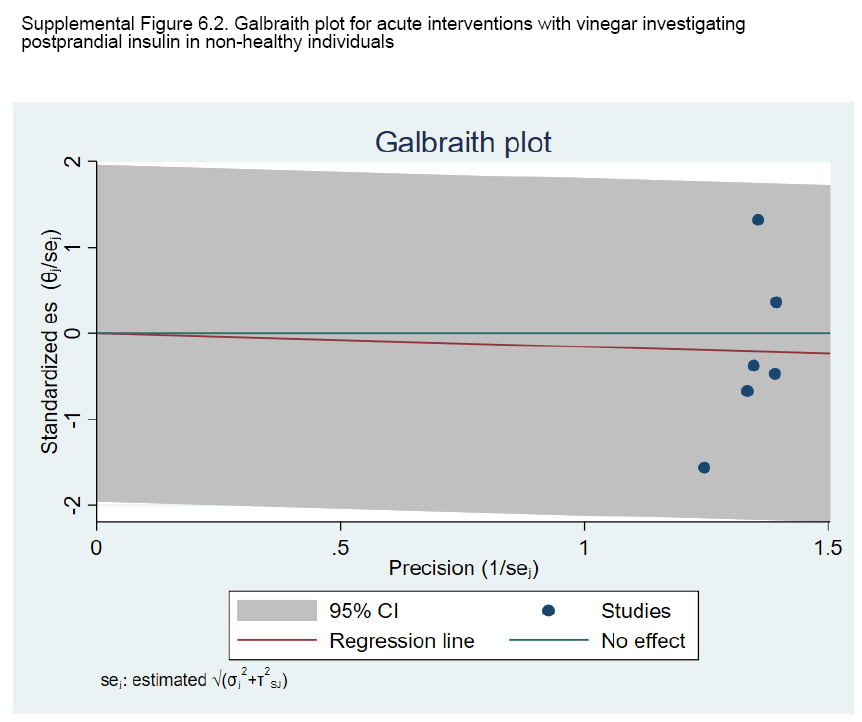
**Supplemental Figure 6.2.** **Galbriath plot to assess for acute vinegar intake on postprandial blood insulin in non-healthy adults.** n=58. CI, confidence interval.


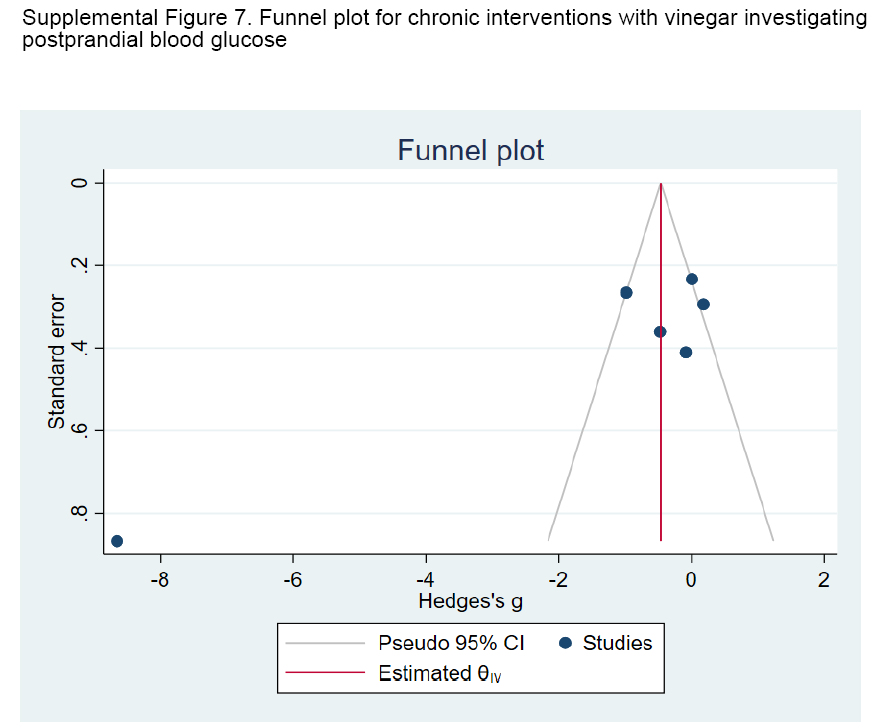


**Supplemental Figure 7.** **Funnel plots to assess publication bias in chronic vinegar intake on postprandial blood glucose.** n=143. CI, confidence interval.


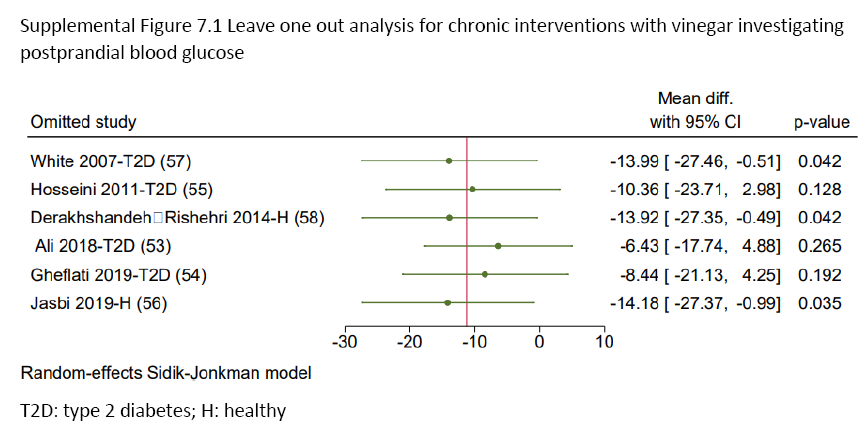
**Supplemental Figure 7.1.** **Leave one out sensitivity analysis for chronic vinegar intake on postprandial blood glucose.** n=143. Random-effects Sidik-Johnkman model. P-value≤0.05 was considered statistically significant. CI, confidence interval, H, healthy, T2D, type 2 diabetes.


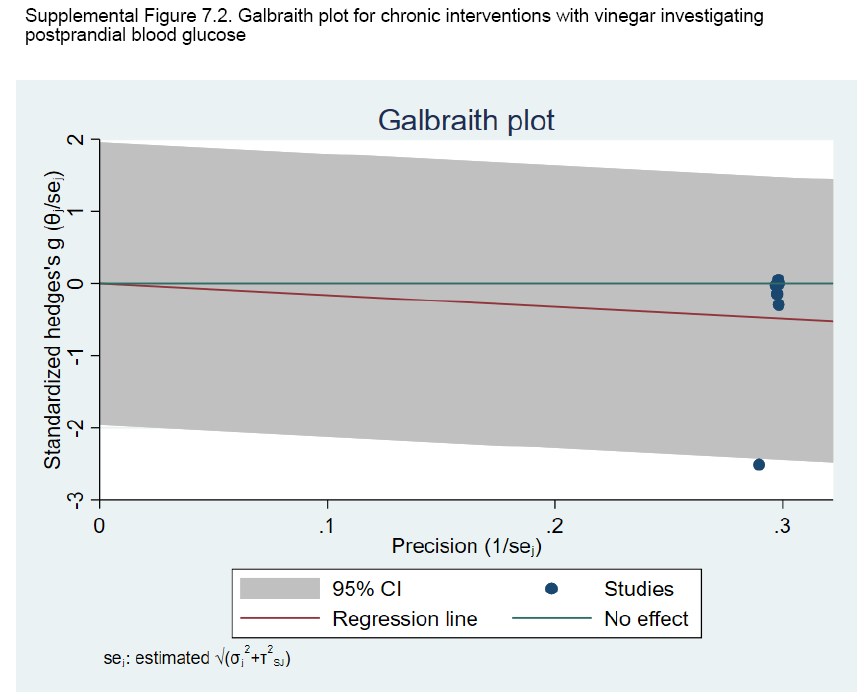
**Supplemental Figure 7.2.** **Galbriath plot to assess for chronic vinegar intake on postprandial blood glucose.** n=143. CI, confidence interval.


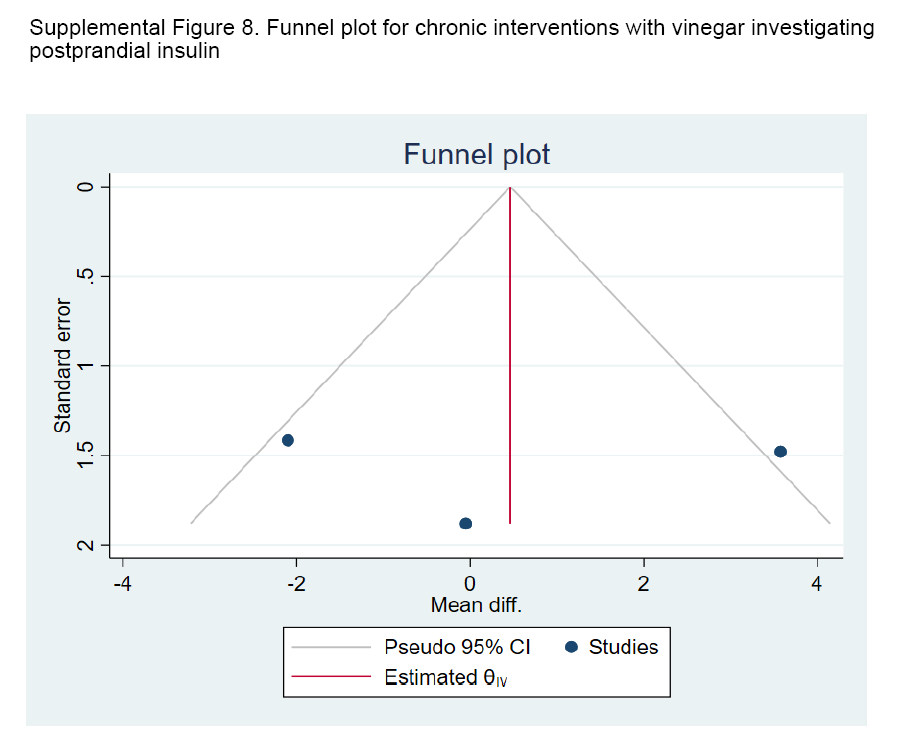
**Supplemental Figure 8.** **Funnel plots to assess publication bias in chronic vinegar intake on postprandial blood insulin.** n=89. CI, confidence interval.


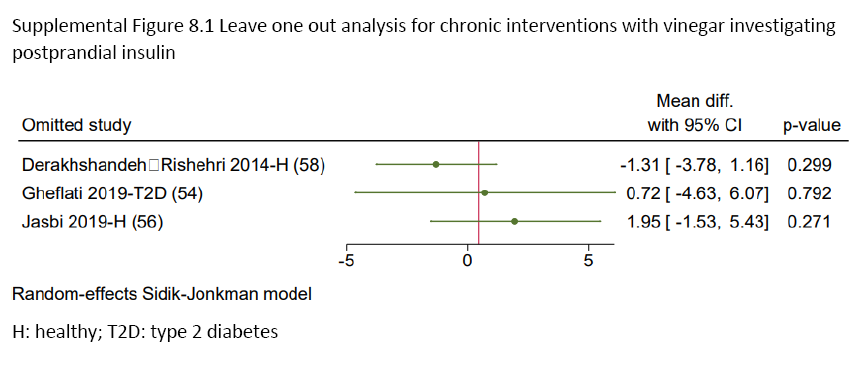
**Supplemental Figure 8.1.** **Leave one out sensitivity analysis for chronic vinegar intake on postprandial blood insulin.** n=89. Random-effects Sidik-Johnkman model. P-value≤0.05 was considered statistically significant. CI, confidence interval, H, healthy, T2D, type 2 diabetes.


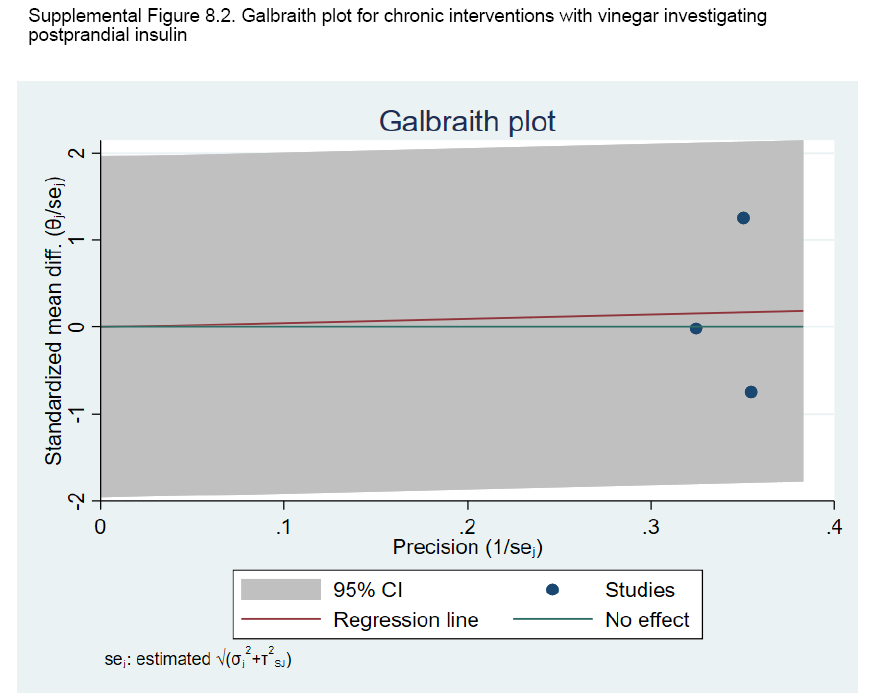
**Supplemental Figure 8.2.** **Galbriath plot to assess for chronic vinegar intake on postprandial blood insulin.** n=89. CI, confidence interval.


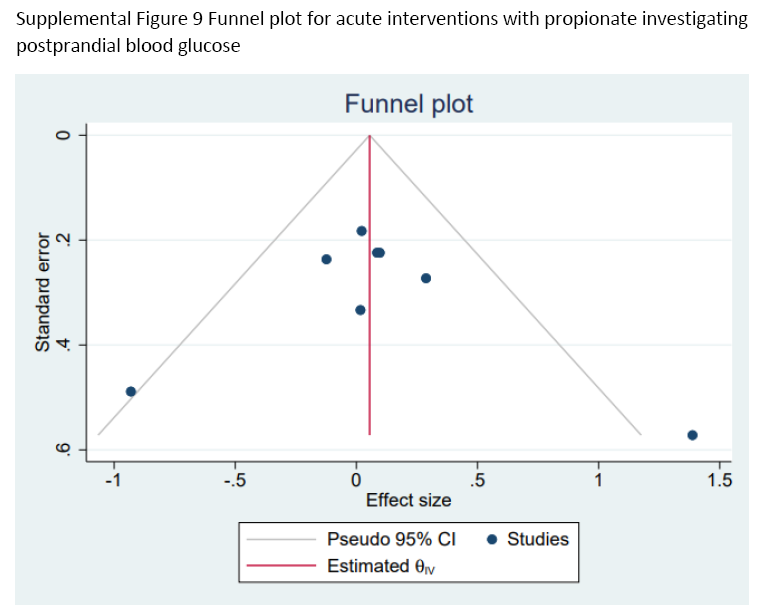
**Supplemental Figure 9. Funnel plots to assess publication bias in acute propionate on postprandial blood glucose.** n=123. CI, confidence interval.


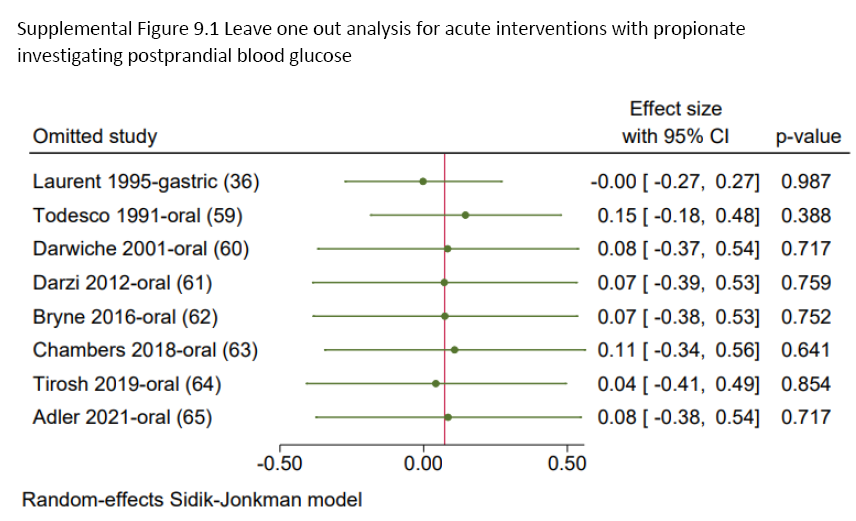
**Supplemental Figure 9.1.** **Leave one out sensitivity analysis for acute propionate on postprandial blood glucose.** n=123. Random-effects Sidik-Johnkman model. P-value≤0.05 was considered statistically significant. CI, confidence interval.


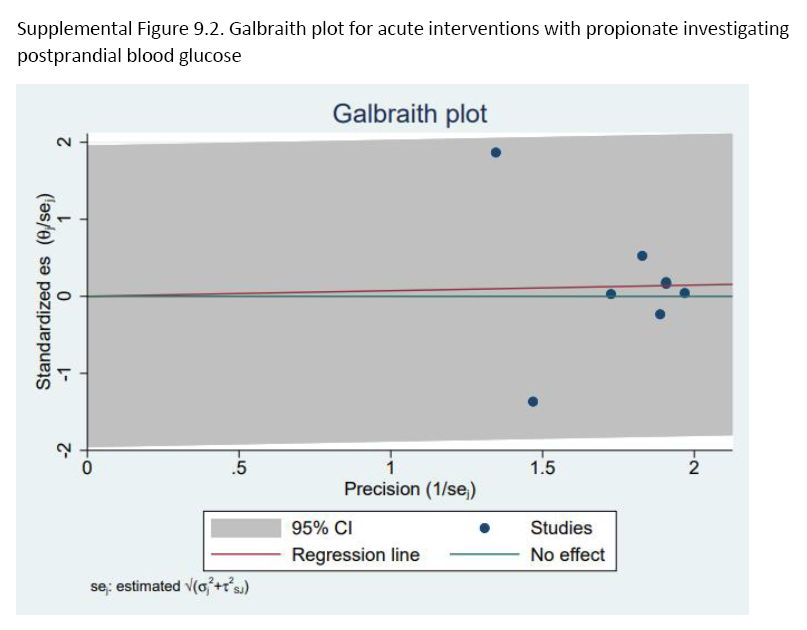
**Supplemental Figure 9.2.** **Galbriath plot to assess for acute propionate on postprandial blood glucose.** n=123. CI, confidence interval.


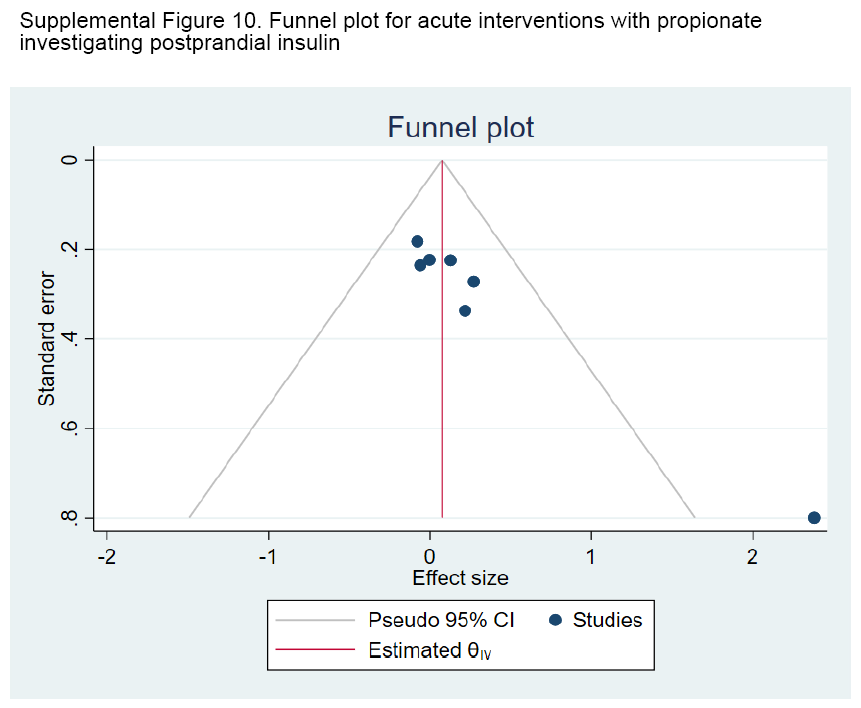
**Supplemental Figure 10.** **Funnel plots to assess publication bias in acute propionate on postprandial blood insulin.** n=117. CI, confidence interval.


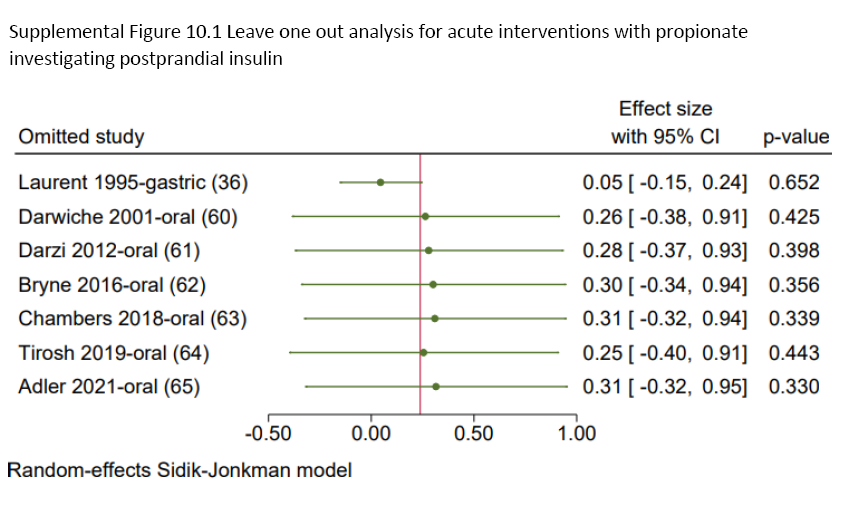
**Supplemental Figure 10.1.** **Leave one out sensitivity analysis for acute propionate on postprandial blood insulin.** n=117. Random-effects Sidik-Johnkman model. P-value≤0.05 was considered statistically significant. CI, confidence interval.


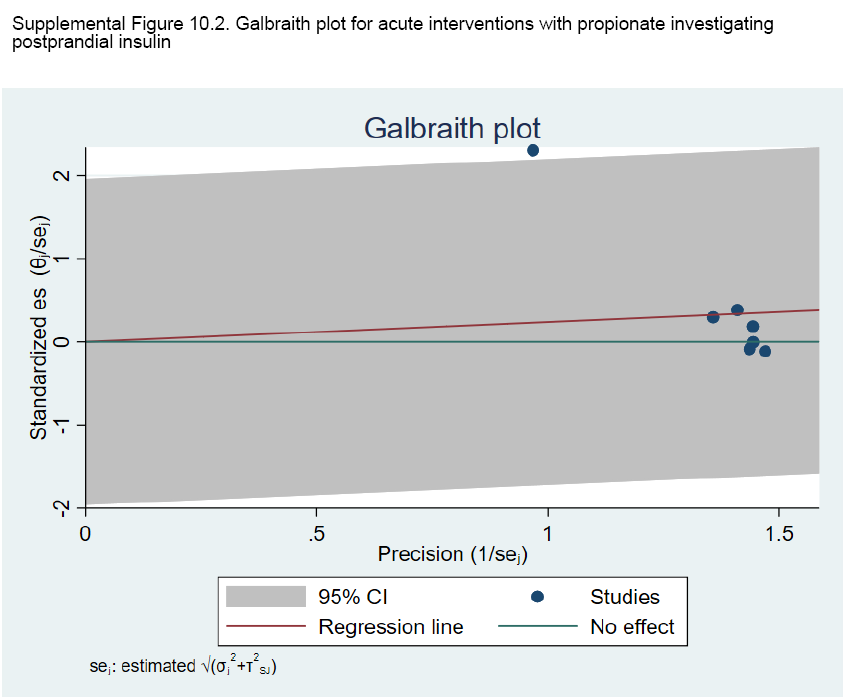
**Supplemental Figure 10.2.** **Galbriath plot to assess for acute propionate on postprandial blood insulin.** n=117. CI, confidence interval.


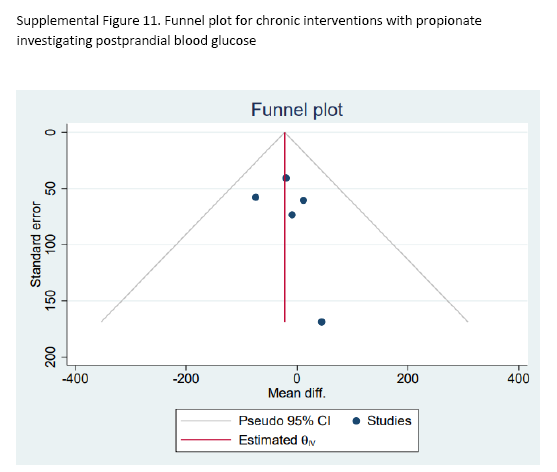


**Supplemental Figure 11.** **Funnel plots to assess publication bias in chronic propionate on postprandial blood glucose.** n=73. CI, confidence interval.


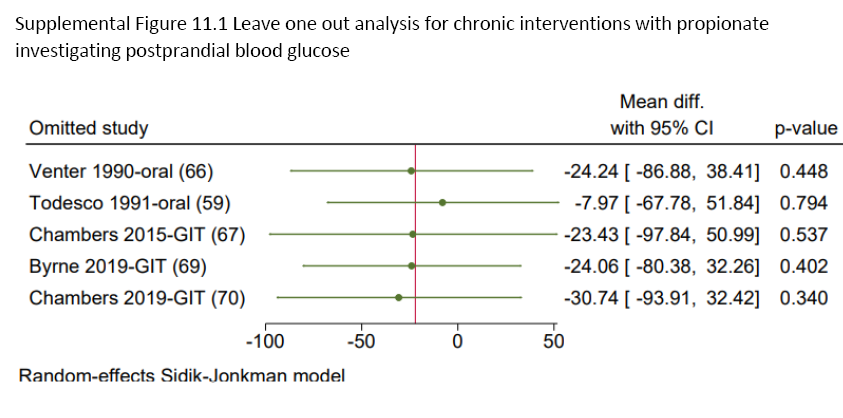
**Supplemental Figure 11.1.** **Leave one out sensitivity analysis for chronic propionate on postprandial blood glucose.** n=73.Random-effects Sidik-Johnkman model. P-value≤0.05 was considered statistically significant. CI, confidence interval.


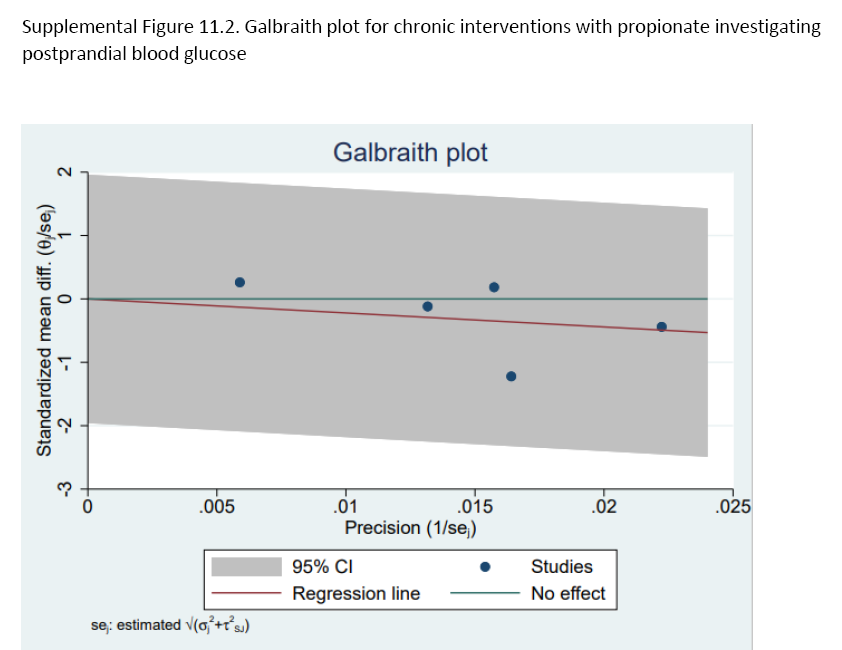
**Supplemental Figure 11.2.** **Galbriath plot to assess for chronic propionate on postprandial blood glucose.** n=73. CI, confidence interval.

**
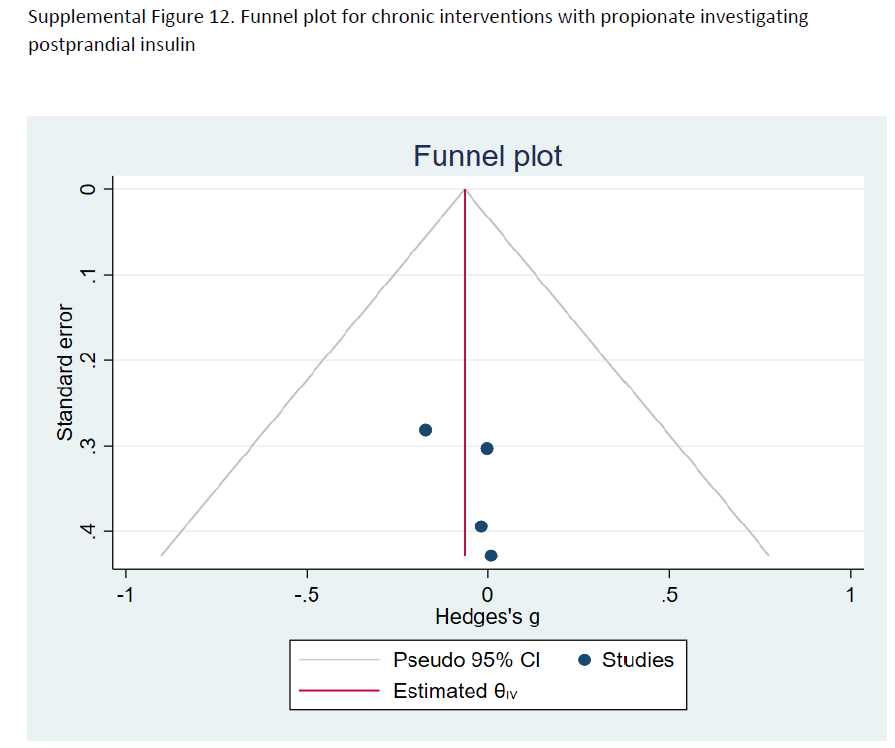
**

**Supplemental Figure 12.** **Funnel plots to assess publication bias in chronic propionate on postprandial blood insulin.** n=117. CI, confidence interval.


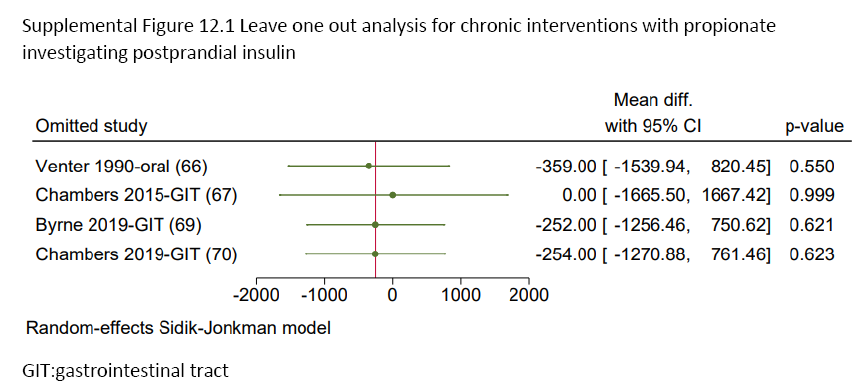
**Supplemental Figure 12.1.** **Leave one out sensitivity analysis for chronic propionate on postprandial blood insulin.** n=117. Random-effects Sidik-Johnkman model. P-value≤0.05 was considered statistically significant. CI, confidence interval, GIT, gastrointestinal tract


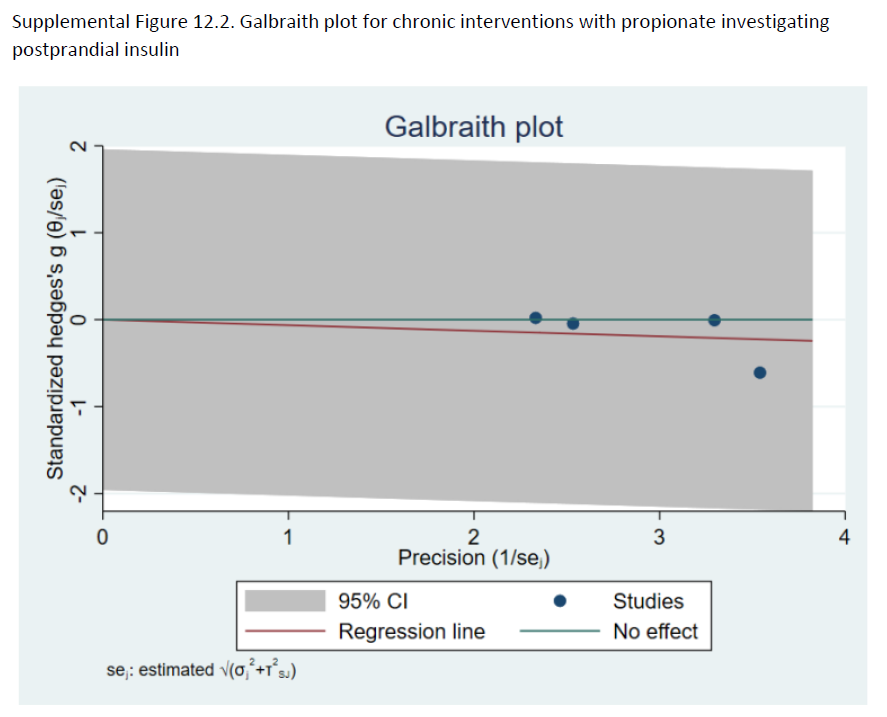
**Supplemental Figure 12.2.** **Galbriath plot to assess for chronic propionate on postprandial blood insulin.** n=117. CI, confidence interval.


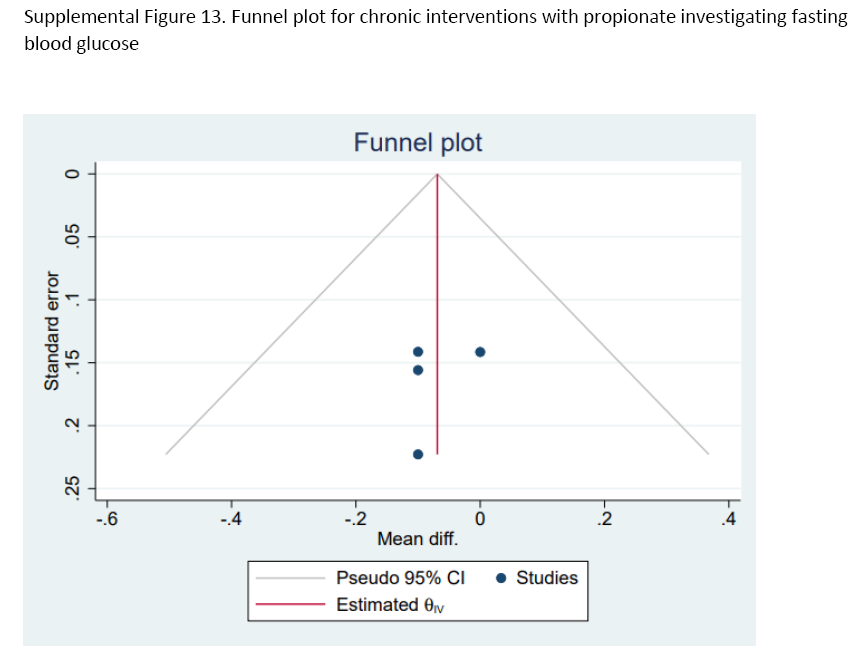


**Supplemental Figure 13.** **Funnel plots to assess publication bias in chronic propionate on fasting blood glucose.** n=67. CI, confidence interval.


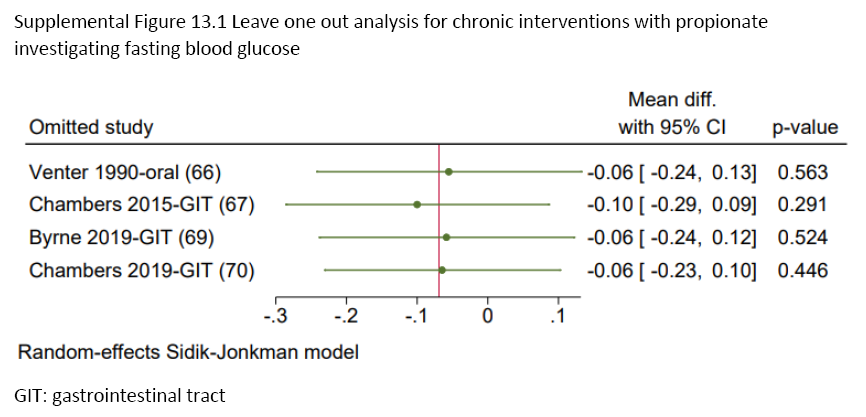


**Supplemental Figure 13.1.** **Leave one out sensitivity analysis for chronic propionate on fasting blood glucose.** n=67. Random-effects Sidik-Johnkman model. P-value≤0.05 was considered statistically significant. CI, confidence interval.


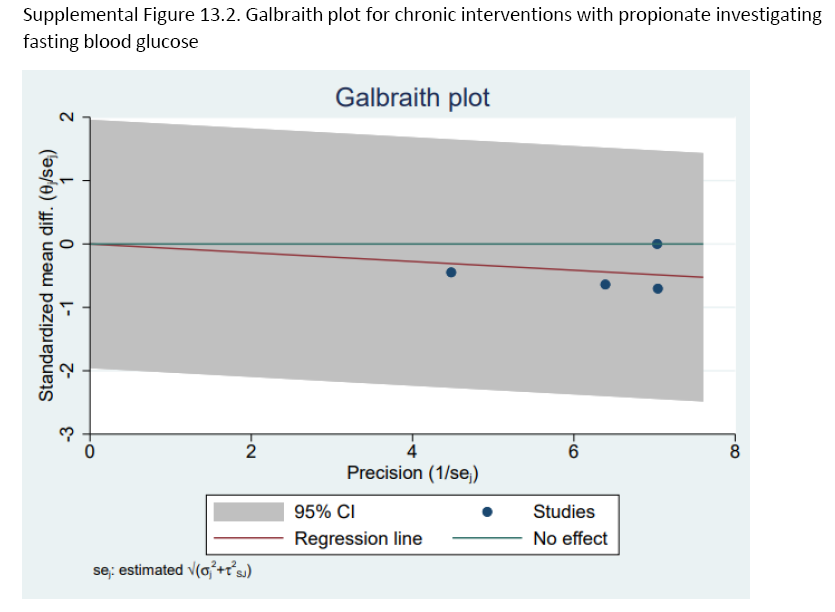
 **Supplemental Figure 13.2.** **Galbriath plot to assess for chronic propionate on fasting blood glucose.** n=67. CI, confidence interval.


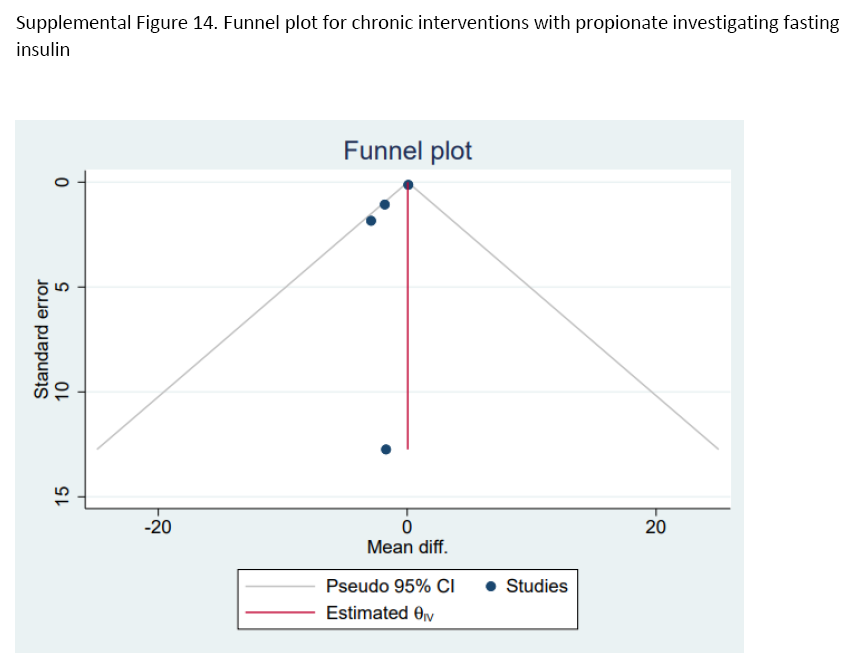
**Supplemental Figure 14.** **Funnel plots to assess publication bias in chronic propionate on fasting blood insulin.** n=67. CI, confidence interval.


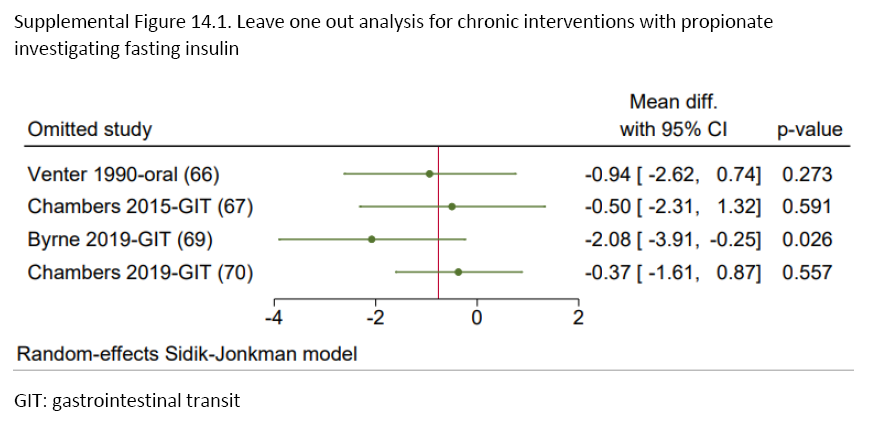
**Supplemental Figure 14.1.** **Leave one out sensitivity analysis for chronic propionate on fasting blood insulin.** n=67. Random-effects Sidik-Johnkman model. P-value≤0.05 was considered statistically significant. CI, confidence interval, GIT, gastrointestinal tract.


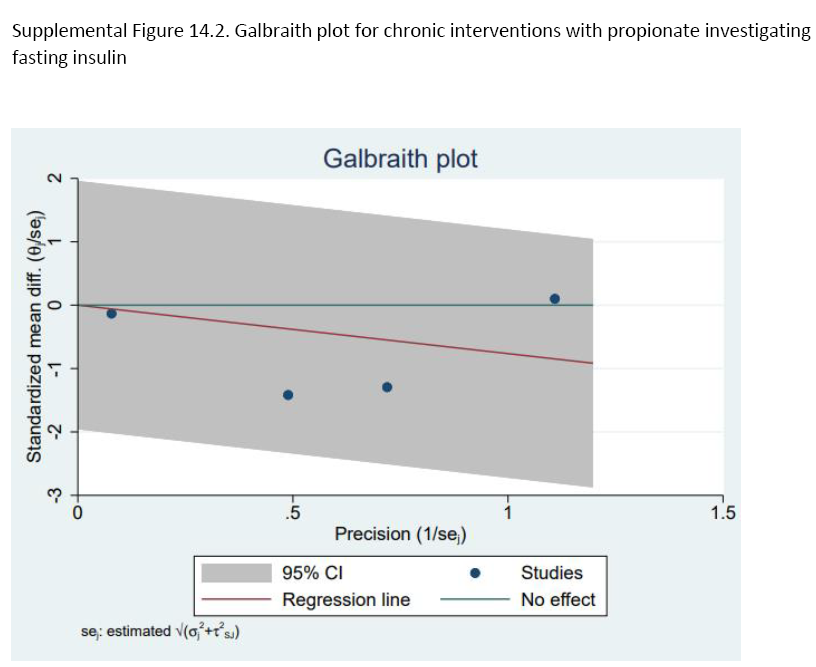
**Supplemental Figure 14.2.** **Galbriath plot to assess for chronic propionate on fasting blood insulin.** n=67. CI, confidence interval.

References

1. Akanji AO, Hockaday TD. Acetate tolerance and the kinetics of acetate utilization in diabetic and nondiabetic subjects. Am J Clin Nutr. 1990 Jan;51(1):112–8.

2. Alamowitch C, Boillot J, Boussairi A, Auboiron S, Guyon F, Chevalier A, Bruzzo F, Rizkalla SW, Bornet FRJ, Slama G. Short chain fatty acids do not alter glucose metabolism but acetate modifies plasma-lipids in humans. 1994;37(Supp 1):A129–A129.

3. Amaral L, Morgan D, Stephen AM, Whiting S. Effect of propionate on lipid-metabolism in healthy-human subjects. FASEB J. 1992;6(5):A1655–A1655.

4. Ball D, Green G. The metabolic response to exercise following sodium acetate ingestion in man. In 2001. p. S213.

5. Pusparatha SB, Devi RG, Jyothipriya A. Effects of apple cider vinegar on diabetic and obese patients. Drug Invention Today. 2019;12(5):3.

6. Bornet FR. Short chain fatty acids and metabolic effects in humans. Gums and stabilisers for food industry. 1994;217–29.

7. Bouter KE, Bakker GJ, Levin E, Hartstra AV, Kootte RS, Udayappan SD, Katiraei S, Bahler L, Gilijamse PW, Tremaroli V, et al. Differential metabolic effects of oral butyrate treatment in lean versus metabolic syndrome subjects. Clinical and Translational Gastroenterology. 2018 May;9(5):e155.

8. Canfora EE, Jocken JW, Blaak EE. Short-chain fatty acids in control of body weight and insulin sensitivity. Nat Rev Endocrinol. 2015 Oct;11(10):577–91.

9. Orals. Diabetes. 2014 Jun 1;63(Supplement_1):A1–102.

10. Cani PD, Van Hul M, Lefort C, Depommier C, Rastelli M, Everard A. Microbial regulation of organismal energy homeostasis. Nature Metabolism. 2019 Jan;1(1):34–46.

11. Chambers ES, Byrne CS, Rugyendo A, Morrison DJ, Preston T, Tedford C, Bell JD, Thomas L, Akbar AN, Riddell NE, et al. The effects of dietary supplementation with inulin and inulin-propionate ester on hepatic steatosis in adults with non-alcoholic fatty liver disease. Diabetes Obes Metab. 2019 Feb;21(2):372–6.

12. Diaz-Buxo JA, Greenberg J, Ho C-H, Howson L, Sergeyeva O, Hinsu P, Mullon C. A Randomized, Controlled, Three-arm, Open-label, Cross-over Bioequivalence Study Comparing Calcium Acetate Oral Solution and Calcium Acetate Gelcaps in Healthy Volunteers. 2012;(2):10.

13. Fernandes J, Vogt J, Wolever TMS. Intravenous acetate elicits a greater free fatty acid rebound in normal than hyperinsulinaemic humans. Eur J Clin Nutr. 2012 Sep;66(9):1029–34.

14. Johnston C. A comparison of liquid vinegar and commercial vinegar pills for managing insulin resistance and postprandial glucose concentrations [Internet]. Wiley Online Library. [cited 2022 Feb 22]. Available from: https://faseb.onlinelibrary.wiley.com/doi/abs/10.1096/fasebj.2018.32.1_supplement.925.10

15. Khatib M, Chambers E, Morrison DJ, Frost G. A pilot study to evaluate the effect of increased colonic propionate on glucose homeostasis during a hypocaloric diet. :1.

16. Kohn JB. Is Vinegar an Effective Treatment for Glycemic Control or Weight Loss? Journal of the Academy of Nutrition and Dietetics. 2015 Jul;115(7):1188.

17. Liljeberg H, Björck I. Delayed gastric emptying rate may explain improved glycaemia in healthy subjects to a starchy meal with added vinegar. Eur J Clin Nutr. 1998 May;52(5):368–71.

18. Lim J, Henry CJ, Haldar S. Vinegar as a functional ingredient to improve postprandial glycemic control—human intervention findings and molecular mechanisms. Molecular Nutrition & Food Research. 2016;60(8):1837–49.

19. Malkova D, Polyviou T, Rizou E, Gerasimidis K, Chambers ES, Preston T, Tedford CM, Frost G, Morrison DJ. Moderate intensity exercise training combined with inulin-propionate ester supplementation increases whole body resting fat oxidation in overweight women. Metabolism. 2020 Mar 1;104:154043.

20. Marzocco S, Fazeli G, Di Micco L, Autore G, Adesso S, Dal Piaz F, et al. Supplementation of Short-Chain Fatty Acid, Sodium Propionate, in Patients on Maintenance Hemodialysis: Beneficial Effects on Inflammatory Parameters and Gut-Derived Uremic Toxins, A Pilot Study (PLAN Study). J Clin Med. 2018 Sep 30;7(10):E315.

21. Mitrou P, Raptis AE, Lambadiari V, Boutati E, Petsiou E, Spanoudi F, Papakonstantinou E, Maratou E, Economopoulos T, Dimitriadis G, et al. Vinegar Decreases Postprandial Hyperglycemia in Patients With Type 1 Diabetes. Diabetes Care. 2010 Feb 1;33(2):e27–e27.

22. Pagliai G, Dinu M, Fiorillo C, Becatti M, Turroni S, Emmi G, Francesco S. Modulation of gut microbiota through nutritional interventions in Behçet’s syndrome patients (the MAMBA study): study protocol for a randomized controlled trial. Trials. 2020 Dec;21(1):511.

23. Petersen KF, Impellizeri A, Cline GW, Shulman GI. The effects of increased acetate turnover on glucose-induced insulin secretion in lean and obese humans. J Clin Trans Sci. 2019 Feb;3(1):18–20.

24. Petsiou EI, Mitrou PI, Raptis SA, Dimitriadis GD. Effect and mechanisms of action of vinegar on glucose metabolism, lipid profile, and body weight. Nutrition Reviews. 2014 Oct 1;72(10):651–61.

25. Piccardo MG, Onori L, Di Addario A, Legramante A, Ciampalini L. Aspects of carbohydrate and lipid metabolism in the obese, studied with acetate-2-14C and glycerol-2-3H. Folia Endocrinologica. 1967;20:510–20.

26. Polyviou T, MacDougall K, Chambers ES, Viardot A, Psichas A, Jawaid S, Harris HC, Edwards CA, Simpson L, Murphy KG, et al. Randomised clinical study: inulin short-chain fatty acid esters for targeted delivery of short-chain fatty acids to the human colon. Aliment Pharmacol Ther. 2016 Oct;44(7):662–72.

27. Salbe AD, Johnston CS, Buyukbese MA, Tsitouras PD, Harman SM. Vinegar lacks antiglycemic action on enteral carbohydrate absorption in human subjects. Nutrition Research. 2009 Dec;29(12):846–9.

28. Sasaki M, Sugiyama T, Noguchi S, Kitahora H, Shimozata A, Yamaguchi Y, Kasugai K. Sa1984 Influence of Intestinal Microbiome and Short Chain Fatty Acid on Metabolism in Type 2 Diabetes Patients. Gastroenterology. 2016;150(4):S424.

29. Smith GI, Jeukendrup AE, Ball D. Sodium Acetate Induces a Metabolic Alkalosis but Not the Increase in Fatty Acid Oxidation Observed Following Bicarbonate Ingestion in Humans. The Journal of Nutrition. 2007 Jul 1;137(7):1750–6.

30. Sugiyama M, Tang AC, Wakaki Y, Koyama W. Glycemic index of single and mixed meal foods among common Japanese foods with white rice as a reference food. Eur J Clin Nutr. 2003 Jun;57(6):743–52.

31. Todesco T, Zamboni M, Armellini F, Bissoli L, Turcato E, Piemonte G, Rao AV, Jenkins DJ, Bosello O. Plasma acetate levels in a group of obese diabetic, obese normoglycemic, and control subjects and their relationships with other blood parameters. Am J Gastroenterol. 1993 May;88(5):751–5.

31. Vahid H, Bonakdaran S, Khorasani ZM, Jarahi L, Rakhshandeh H, Ghorbani A, Zaghi N, Yousefi M. Effect of Capparis spinosa Extract on Metabolic Parameters in Patients with Type-2 Diabetes: A Randomized Controlled Trial. Endocr Metab Immune Disord Drug Targets. 2019;19(1):100–7.

32. van der Beek CM, Canfora EE, Lenaerts K, Troost FJ, Holst JJ, Masclee AAM, Dejong CHC, Blaak EE. OP038: Colonic Acetate Infusions Promote Fat Oxidation and Improve Metabolic Parameters in Overweight Males. Clinical Nutrition. 2014 Sep;33:S16–7.

34. Yki-Jarvinen H, Koivisto VA, Ylikahri R, Taskinen MR. Acute effects of ethanol and acetate on glucose kinetics in normal subjects. American Journal of Physiology-Endocrinology and Metabolism. 1988 Feb 1;254(2):E175–80.

35. Scheppach W, Cummings JH, Branch WJ, Schrezenmeir J. Effect of gut-derived acetate on oral glucose tolerance in man. Clin Sci (Lond). 1988 Oct;75(4):355–61.

36. Laurent C, Simoneau C, Marks L, Braschi S, Champ M, Charbonnel B, Krempf M. Effect of acetate and propionate on fasting hepatic glucose production in humans. Eur J Clin Nutr. 1995 Jul;49(7):484–91.

37. Freeland KR, Wolever TMS. Acute effects of intravenous and rectal acetate on glucagon-like peptide-1, peptide YY, ghrelin, adiponectin and tumour necrosis factor-α. British Journal of Nutrition. 2010 Feb;103(3):460–6.

38. Johnston CS, Steplewska I, Long CA, Harris LN, Ryals RH. Examination of the Antiglycemic Properties of Vinegar in Healthy Adults. Ann Nutr Metab. 2010;56(1):74–9.

39. van der Beek CM, Canfora EE, Lenaerts K, Troost FJ, Damink SWMO, Holst JJ, Masclee AAM, Dejong C, Blaak EE. Distal, not proximal, colonic acetate infusions promote fat oxidation and improve metabolic markers in overweight/obese men. Clin Sci (Lond). 2016 Nov 1;130(22):2073–82.

40. Brighenti F, Castellani G, Benini L, Casiraghi MC, Leopardi E, Crovetti R, Testolin G. Effect of neutralized and native vinegar on blood glucose and acetate responses to a mixed meal in healthy subjects. Eur J Clin Nutr. 1995 Apr;49(4):242–7.

41. Johnston CS, Kim CM, Buller AJ. Vinegar Improves Insulin Sensitivity to a High-Carbohydrate Meal in Subjects With Insulin Resistance or Type 2 Diabetes. Diabetes Care. 2004 Jan 1;27(1):281–2.

42. Johnston CS, Buller AJ. Vinegar and Peanut Products as Complementary Foods to Reduce Postprandial Glycemia. Journal of the American Dietetic Association. 2005 Dec;105(12):1939–42.

43. Östman E, Granfeldt Y, Persson L, Björck I. Vinegar supplementation lowers glucose and insulin responses and increases satiety after a bread meal in healthy subjects. Eur J Clin Nutr. 2005 Sep;59(9):983–8.

44. Leeman M, Östman E, Björck I. Vinegar dressing and cold storage of potatoes lowers postprandial glycaemic and insulinaemic responses in healthy subjects. Eur J Clin Nutr. 2005 Nov;59(11):1266–71.

45. Hlebowicz J, Darwiche G, Björgell O, Almér L-O. Effect of apple cider vinegar on delayed gastric emptying in patients with type 1 diabetes mellitus: a pilot study. BMC Gastroenterology. 2007 Dec 20;7(1):46.

46. Mettler S, Schwarz I, Colombani PC. Additive postprandial blood glucose-attenuating and satiety-enhancing effect of cinnamon and acetic acid. Nutr Res. 2009 Oct;29(10):723–7.

47. Liatis S, Grammatikou S, Poulia K-A, Perrea D, Makrilakis K, Diakoumopoulou E, Katsilambros N. Vinegar reduces postprandial hyperglycaemia in patients with type II diabetes when added to a high, but not to a low, glycaemic index meal. Eur J Clin Nutr. 2010 Jul;64(7):727–32.

48. Darzi J, Frost GS, Montaser R, Yap J, Robertson MD. Influence of the tolerability of vinegar as an oral source of short-chain fatty acids on appetite control and food intake. Int J Obes. 2014 May;38(5):675–81.

49. Mitrou P, Petsiou E, Papakonstantinou E, Maratou E, Lambadiari V, Dimitriadis P, Spanoudi F, Raptis SA, Dimitriadis G. Vinegar Consumption Increases Insulin-Stimulated Glucose Uptake by the Forearm Muscle in Humans with Type 2 Diabetes. Journal of Diabetes Research. 2015;2015:1–7.

50. Mitrou P, Petsiou E, Papakonstantinou E, Maratou E, Lambadiari V, Dimitriadis P, Spanoudi F, Raptis SA, Dimitriadis G. The role of acetic acid on glucose uptake and blood flow rates in the skeletal muscle in humans with impaired glucose tolerance. Eur J Clin Nutr. 2015 Jun;69(6):734–9.

51. Zhao W, Wang L, Fan Z, Lu J, Zhu R, Wu Y, Lu X. Co-ingested vinegar-soaked or preloaded dried apple mitigated acute postprandial glycemia of rice meal in healthy subjects under equicarbohydrate conditions. Nutrition Research. 2020 Nov;83:108–18.

52. Feise NK, Johnston CS. Commercial Vinegar Tablets Do Not Display the Same Physiological Benefits for Managing Postprandial Glucose Concentrations as Liquid Vinegar. Suzuki T, editor. Journal of Nutrition and Metabolism. 2020 Dec 16;2020:1–5.

53. Ali Z, Ma H, Wali A, Ayim I, Rashid MT, Younas S. A double-blinded, randomized, placebo-controlled study evaluating the impact of dates vinegar consumption on blood biochemical and hematological parameters in patients with type 2 diabetes. Trop J Pharm Res. 2019 Mar 15;17(12):2463.

54. Gheflati A, Bashiri R, Ghadiri-Anari A, Reza JZ, Kord MT, Nadjarzadeh A. The effect of apple vinegar consumption on glycemic indices, blood pressure, oxidative stress, and homocysteine in patients with type 2 diabetes and dyslipidemia: A randomized controlled clinical trial. Clinical Nutrition ESPEN. 2019 Oct;33:132–8.

55. Hosseini ZSM, Hosseini J, Nabati S, Hasanshahi G, Mahmoodi M. Survey on the anti-diabetic effects of vinegar on some biochemical factors in type 2 diabetic patients. Clinical Biochemistry. 2011 Sep;44(13):S226.

56. Jasbi P, Baker O, Shi X, Gonzalez LA, Wang S, Anderson S, Xi B, Gu H, Johnston CS. Daily red wine vinegar ingestion for eight weeks improves glucose homeostasis and affects the metabolome but does not reduce adiposity in adults. Food Funct. 2019;10(11):7343–55.

57. White AM, Johnston CS. Vinegar Ingestion at Bedtime Moderates Waking Glucose Concentrations in Adults With Well-Controlled Type 2 Diabetes. Diabetes Care. 2007 Nov 1;30(11):2814–5.

58. Derakhshandeh‑Rishehri S, Heidari‑Beni M, Feizi A, Askari G-R, Entezari M. Effect of Honey Vinegar Syrup on Blood Sugar and Lipid Profile in Healthy. International Journal of Preventive Medicine. 2014;5(12):9.

59. Todesco T, Rao AV, Bosello O, Jenkins DJ. Propionate lowers blood glucose and alters lipid metabolism in healthy subjects. Am J Clin Nutr. 1991 Nov;54(5):860–5.

60. Darwiche G, Östman EM, Liljeberg HG, Kallinen N, Björgell O, Björck IM, Almér LO. Measurements of the gastric emptying rate by use of ultrasonography: studies in humans using bread with added sodium propionate. The American Journal of Clinical Nutrition. 2001 Aug 1;74(2):254–8.

61. Darzi J, Frost GS, Robertson MD. Effects of a novel propionate-rich sourdough bread on appetite and food intake. Eur J Clin Nutr. 2012 Jul;66(7):789–94.

62. Byrne CS, Chambers ES, Alhabeeb H, Chhina N, Morrison DJ, Preston T, Tedford C, Fitzpatrick J, Irani C, Busza A, et al. Increased colonic propionate reduces anticipatory reward responses in the human striatum to high-energy foods. Am J Clin Nutr. 2016 Jul 1;104(1):5–14.

63. Chambers ES, Byrne CS, Aspey K, Chen Y, Khan S, Morrison DJ, Frost G. Acute oral sodium propionate supplementation raises resting energy expenditure and lipid oxidation in fasted humans. Diabetes Obes Metab. 2018 Apr;20(4):1034–9.

64. Tirosh A, Calay ES, Tuncman G, Claiborn KC, Inouye KE, Eguchi K, Alcala M, Rathaus M, Hollander KS, Ron I, et al. The short-chain fatty acid propionate increases glucagon and FABP4 production, impairing insulin action in mice and humans. Sci Transl Med. 2019 Apr 24;11(489):eaav0120.

65. Adler GK, Hornik ES, Murray G, Bhandari S, Yadav Y, Heydarpour M, Basu R, Garg R, Tirosh A. Acute effects of the food preservative propionic acid on glucose metabolism in humans. BMJ Open Diab Res Care. 2021 Jul;9(1):e002336.

66. Venter CS, Vorster HH, Cummings JH. Effects of dietary propionate on carbohydrate and lipid metabolism in healthy volunteers. Am J Gastroenterol. 1990 May;85(5):549–53.

67. Chambers ES, Viardot A, Psichas A, Morrison DJ, Murphy KG, Zac-Varghese SEK, MacDougall K, Preston T, Tedford C, Finlayson GS, et al. Effects of targeted delivery of propionate to the human colon on appetite regulation, body weight maintenance and adiposity in overweight adults. Gut. 2015 Nov 1;64(11):1744–54.

68. Pingitore A, Chambers ES, Hill T, Maldonado IR, Liu B, Bewick G, Morrison DJ, Preston T, Wallis GA, Tedford C, et al. The diet-derived short chain fatty acid propionate improves beta-cell function in humans and stimulates insulin secretion from human islets in vitro: PINGITORE et al. Diabetes Obes Metab. 2017 Feb;19(2):257–65.

69. Byrne CS, Chambers ES, Preston T, Tedford C, Brignardello J, Garcia-Perez I, Holmes E, Wallis GA, Morrison DJ, Frost GS. Effects of Inulin Propionate Ester Incorporated into Palatable Food Products on Appetite and Resting Energy Expenditure: A Randomised Crossover Study. Nutrients. 2019 Apr 16;11(4):E861.

70. Chambers ES, Byrne CS, Morrison DJ, Murphy KG, Preston T, Tedford C, Garcia-Perez I,  Fountana S, Serrano-Contreras SJ, Holmes E, et al. Dietary supplementation with inulin-propionate ester or inulin improves insulin sensitivity in adults with overweight and obesity with distinct effects on the gut microbiota, plasma metabolome and systemic inflammatory responses: a randomised cross-over trial. Gut. 2019 Aug 1;68(8):1430–8.

71. Roshanravan N, Mahdavi R, Alizadeh E, Jafarabadi M, Hedayati M, Ghavami A, Alipour S, Alamdari N, Barati M, Ostadrahimi A. Effect of Butyrate and Inulin Supplementation on Glycemic Status, Lipid Profile and Glucagon-Like Peptide 1 Level in Patients with Type 2 Diabetes: A Randomized Double-Blind, Placebo-Controlled Trial. Horm Metab Res. 2017 Nov;49(11):886–91.

72. Roshanravan N, Mahdavi R, Jafarabadi MA, Alizadeh E, Ghavami A, Saadat YR, Alamdari NM, Dastouri MR, Alipour S, Ostadrahimi A. The effects of sodium butyrate and high-performance inulin supplementation on the promotion of gut bacterium Akkermansia muciniphila growth and alterations in miR-375 and KLF5 expression in type 2 diabetic patients: A randomized, double-blind, placebo-controlled trial. European Journal of Integrative Medicine. 2018 Feb;18:1–7.

73. Wolever TM, Jenkins DJ, Ocana AM, Rao VA, Collier GR. Second-meal effect: low-glycemic-index foods eaten at dinner improve subsequent breakfast glycemic response. The American Journal of Clinical Nutrition. 1988 Oct 1;48(4):1041–7.

74. Wolever TMS, Spadafora P, Eshuis H. Interaction between colonic acetate and propionate in humans. The American Journal of Clinical Nutrition. 1991 Mar 1;53(3):681–7.

75. Alamowitch C, Boillot J, Boussairi A, Ruskone-Fourmestraux A, Chevalier A, Rizkalla SW, Guyon F, Bornet FR, Slama G. Lack of effect of an acute ileal perfusion of short-chain fatty acids on glucose metabolism in healthy men. American Journal of Physiology-Endocrinology and Metabolism. 1996 Jul 1;271(1):E199–204.

76. Canfora EE, van der Beek CM, Jocken JWE, Goossens GH, Holst JJ, Olde Damink SWM, Lenaerts K, Dejong CHC, Blaak EE. Colonic infusions of short-chain fatty acid mixtures promote energy metabolism in overweight/obese men: a randomized crossover trial. Sci Rep. 2017 May 24;7(1):2360.
